# Novel artificial metalloenzymes by *in vivo* incorporation of metal-binding unnatural amino acids†
†Electronic supplementary information (ESI) available. See DOI: 10.1039/c4sc01525h



**DOI:** 10.1039/c4sc01525h

**Published:** 2014-10-09

**Authors:** Ivana Drienovská, Ana Rioz-Martínez, Apparao Draksharapu, Gerard Roelfes

**Affiliations:** a Stratingh Institute for Chemistry , University of Groningen , Nijenborgh 4 , 9747 AG Groningen , The Netherlands . Email: j.g.roelfes@rug.nl ; http://roelfesgroup.nl

## Abstract

Artificial metalloenzymes have emerged as an attractive new approach to enantioselective catalysis. Herein, we introduce a novel strategy for preparation of artificial metalloenzymes utilizing amber stop codon suppression methodology for the *in vivo* incorporation of metal-binding unnatural amino acids. The resulting artificial metalloenzymes were applied in catalytic asymmetric Friedel–Crafts alkylation reactions and up to 83% ee for the product was achieved.

## Abstract 2

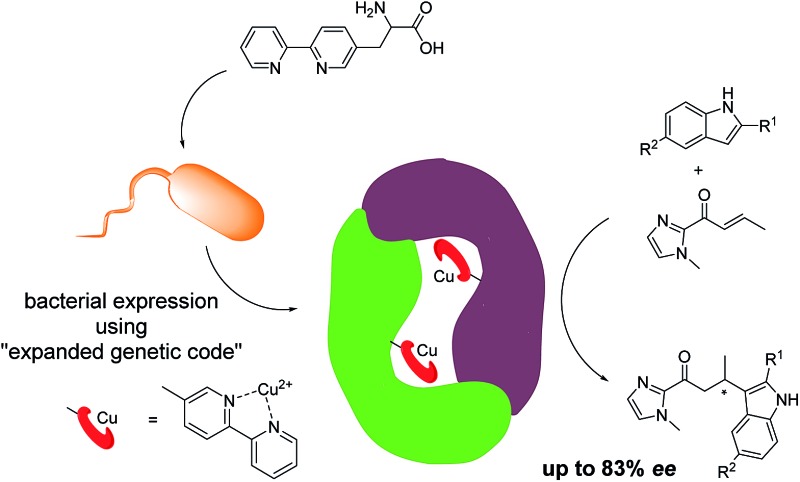
Artificial metalloenzymes for asymmetric catalysis were created by *in vivo* incorporation of unnatural metal binding amino acids into LmrR.

## Introduction

In the quest for more active and selective catalysts, artificial metalloenzymes are emerging as an attractive new concept that merges the catalytic versatility of transition metal catalysts with the high activity and selectivity of enzymes.^1–6^ Artificial metalloenzymes are created by incorporation of catalytically active transition metal complexes into a biomolecular scaffold, such as a protein. The chiral second coordination sphere provided by the scaffold is a key contributor to the rate acceleration and enantioselectivity achieved in a variety of catalytic asymmetric reactions.^7–9^ To date, incorporation of the transition metal complex in the biomolecular scaffold has been achieved using supramolecular, dative or covalent anchoring approaches, or a combination of these.^2,9–16^ While all these methods have their particular attractive features, they also all have limitations. Here we present a new approach to the construction of artificial metalloenzymes involving *in vivo* incorporation of a non-proteinogenic amino acid capable of binding a metal ion, using the amber stop codon suppression methodology, and their application in catalytic enantioselective Friedel–Crafts reactions in water.

*In vivo* incorporation of a metal binding moiety into the protein scaffold, *i.e.* incorporation during protein biosynthesis, is attractive for several reasons. It offers an exquisite degree of control over the position of the metal complex inside the protein, comparable to covalent and dative anchoring, but with the advantage that no chemical modification and/or subsequent purification steps are required. Also, in contrast to the supramolecular anchoring approach, no specific ligand binding interactions are required; assembly of the artificial metalloenzyme is readily achieved by addition of the transition metal salt. Finally, the use of unnatural metal binding amino acids allows for better control over the first coordination sphere compared to using canonical amino acids alone.^17^ Combined these attractive features of the *in vivo* incorporation of metal-binding unnatural amino acids approach greatly facilitate the design and optimization of novel artificial metalloenzymes.

The amber stop codon suppression methodology, also known as expanded genetic code methodology, was introduced by Schultz and coworkers.^18^ It relies on orthogonal tRNA/aminoacyl-tRNA synthetase (aaRS) pairs for the site-specific incorporation of non-proteinogenic amino acids in response to the amber stop codon. Since then, a wide variety of non-proteinogenic amino acids have been genetically encoded in *E. coli*, yeast or mammalian cells, giving rise to novel protein structure, function and applications.^19^ *In vivo* incorporation of metal binding amino acids in proteins has been used for applications including as an artificial nuclease, biophysical or electron-transfer probes, as purification tag and others.^20–28^ Recently, Lewis and co-workers have reported a novel design of artificial metalloenzyme containing the unnatural amino acid *p*-azido-l-phenylalanine in its scaffold, which was used for covalent anchoring of metal binding ligands using a strain-promoted azide–alkyne cycloaddition.^29^ The resulting artificial metalloenzyme was evaluated in dirhodium-catalyzed carbene insertion reaction, however only low conversions and negligible enantioselectivities were observed. To the best of our knowledge, *in vivo* incorporation of metal binding amino acids for the construction of artificial metalloenzymes for enantioselective catalysis has not been reported to date.

We have recently introduced a new design of an artificial metalloenzyme based on the creation of a novel active site on the dimer interface of the transcription factor Lactoccocal multidrug resistance Regulator (LmrR).^30,31^ The original design involved covalent anchoring of Cu(ii)-phenanthroline and Cu(ii)-2,2′-bipyridine complexes to the protein *via* a genetically introduced cysteine residue. These artificial metalloenzymes were successfully used in the catalytic enantioselective Diels–Alder (up to 97% ee) and hydration reactions (up to 84% ee).^31,32^ These results encouraged us to explore LmrR as biomolecular scaffold for artificial metalloenzymes created by *in vivo* incorporation of the non-proteinogenic metal-binding amino acid (2,2′-bipyridin-5yl)alanine (BpyAla).

## Results and discussion

**Fig. 1:**
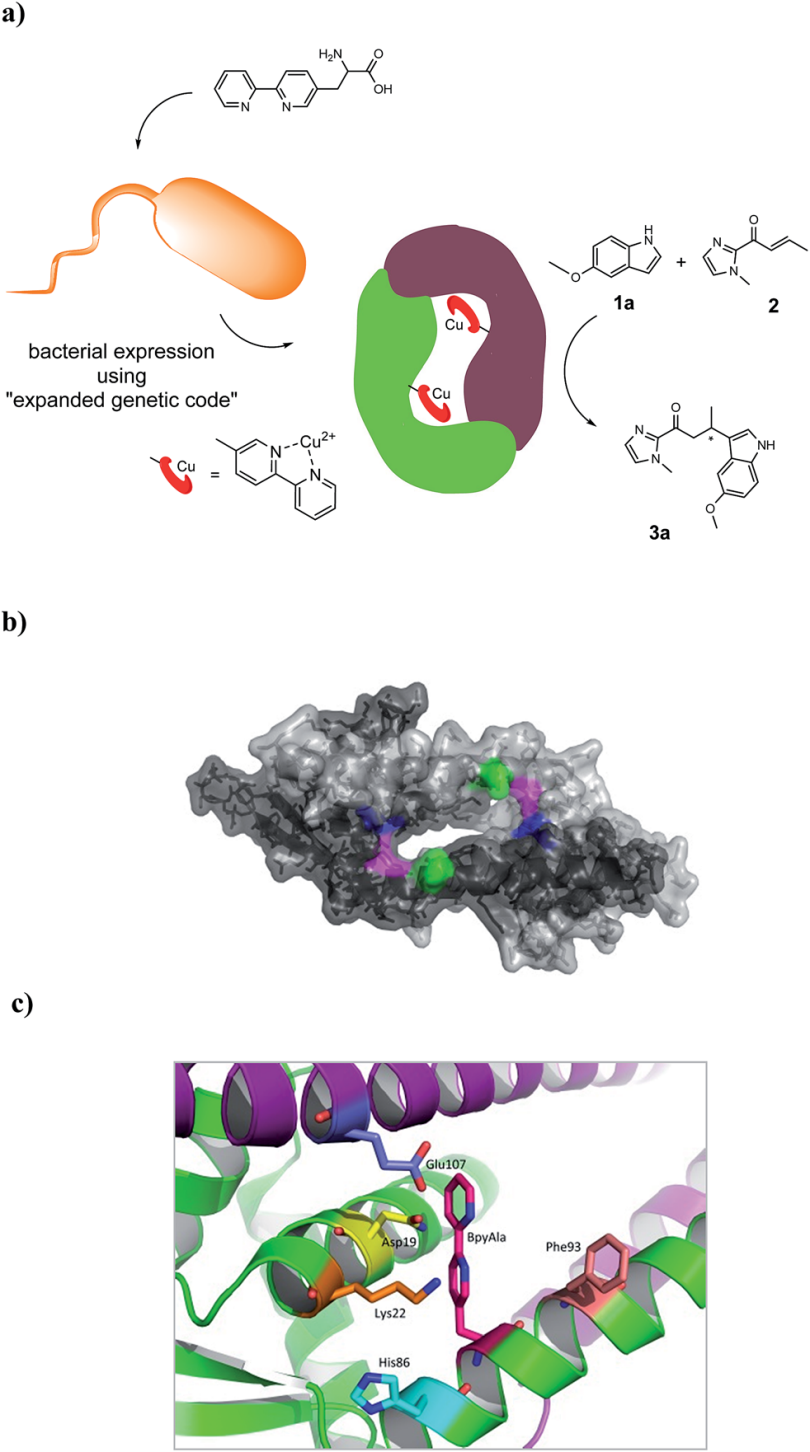
(a) Cartoon representation of the novel artificial metalloenzyme with an *in vivo* incorporated ligand BpyAla and reaction scheme of the benchmark catalytic Friedel–Crafts reaction. (b) Pymol representation of dimeric LmrR (PDB entry ; 3F8B) in space-filling model.^30^ Positions used for incorporation of BpyAla are highlighted in blue (N19), pink (M89) and green (F93). (c) Cartoon representation of LmrR with manually docked BpyAla at position M89. Highlighted residues were used in the mutagenesis study.

The artificial metalloenzyme presented in this study was created by the amber stop codon suppression method using an evolved mutant tRNA/aaRS pair from *Methanococcus jannaschii* (Fig. 1a).^33^ The metal-binding amino acid BpyAla was synthesized according to previously reported methods with small modifications (see ESI†).^34,35^ The design of the artificial metalloenzyme was based on a codon optimized gene of LmrR that included a C-terminal Strep-tag for purification purposes and contained two additional mutations in the DNA binding domain, that are, K55D and K59Q (further referred to as LmrR_LM). These mutations prevent the binding of LmrR_LM to DNA which greatly facilitates purification and, thus, gives rise to higher isolated yields of the protein.^32^ Based on the X-ray structure^30^ and our previous experience, positions N19, M89 and F93 were selected for incorporation of BpyAla. Positions 19 and 89 are located in the hydrophobic pore at the far ends, while position 93 is located on the outside of the front entrance of the pocket (Fig. 1b). An amber stop codon was introduced in the gene at the corresponding positions. The mutations were introduced using standard site-directed mutagenesis techniques (Quick-Change). *E. coli* BL21(DE3)C43 cells were co-transformed with pEVOL-BipyA, the plasmid containing the aaRS and tRNA genes, and the pET17b plasmid containing LmrR gene, and grown in LB media in the presence of 0.5 mM BpyAla.^36^ The proteins were purified by affinity chromatography using a Strep-Tactin sepharose column. Typical purification yields were in the range of 6–18 mg L^–1^, which is only slightly lower than the expression yields of LmrR without BpyAla.^32^ The expression efficiencies were 75–80% for mutants N19BpyAla and F93BpyAla (further referred to as N19X and F93X) and 35–40% for M89BpyAla (further referred to as M89X). The lower expression efficiency of M89X mutant is explained by a higher fraction of the truncated LmrR (1–88), *i.e.* when the TAG codon at position 89 is read as a stop codon. The incorporation of BpyAla in the proteins was confirmed with electrospray ionization mass spectrometry (ESI-MS); in the MS spectrum, no peaks corresponding to alternative amino acid incorporation were observed (Fig. S3†). The quaternary structure of the proteins was evaluated by analytical size-exclusion chromatography on a Superdex-75 10/300 column. All three mutants eluted as a single peak with a molecular weight of approximately 30 kDa, which confirms that the dimeric structure is retained (Fig. S2†).^31,32^ The thermal stability of the LmrR mutant variants was investigated by melting temperature measurement utilizing the thermal shift assay with the Sypro Orange dye.^37^ The melting points of the mutants were determined to be in the range of 47.5–51.3 °C, compared to the 57.5 °C of LmrR_LM. This suggests that the incorporation of BpyAla causes a small destabilization of the LmrR structure, albeit that the combined data shows unambiguously that the dimeric structure of LmrR_LM_X is retained under the conditions of catalysis.

**Fig. 2:**
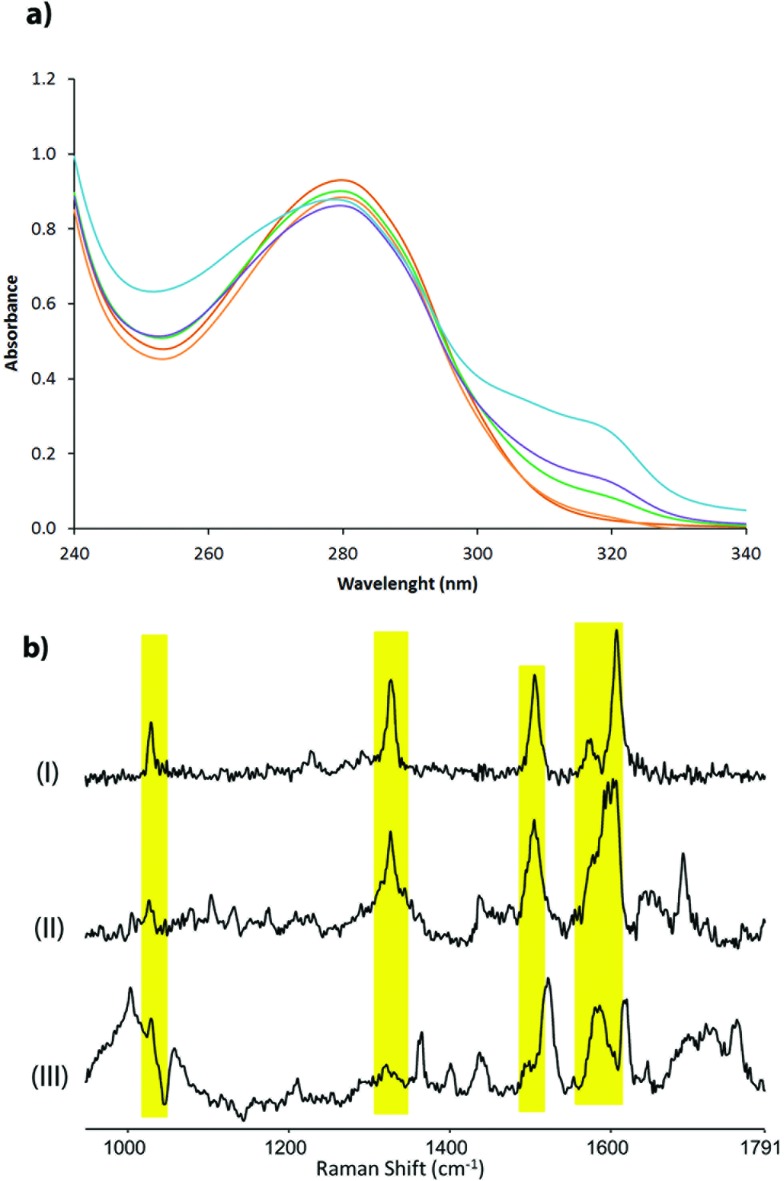
(a) Absorption spectra of LmrR_M89BpyAla (20 μM in monomer) after addition of different concentrations of Cu(NO_3_)_2_: 0 μM (red), 2.5 μM (orange), 5 μM (green), 10 μM (magenta) and 20 μM (blue); (b) resonance Raman spectra of (I) Cu^II^-BpyAla (75 μM), (II) LmrR_M89BpyAla_Cu^II^ (60 μM of Cu^II^) and (III) LmrR_M89BpyAla in 20 mM MOPS buffer, 150 mM NaCl at pH 7 at *λ*_exc_ 355 nm.

The corresponding artificial metalloenzymes were created by addition of 1 equivalent of Cu(NO_3_)_2_ per bipyridine moiety. The binding of Cu^II^ to LmrR_LM_M89X was investigated in more detail and compared to the *in situ* prepared Cu^II^ complex of BpyAla, *i.e.* in the absence of protein. Upon complexation of Cu^II^ to LmrR_LM_M89X, 2 new bands at *λ*_max_ = 307 and 318 nm were observed in the UV/Vis absorption spectrum (Fig. 2a). Addition of more than stoichiometric amounts of Cu^II^ salt with respect to bipyridine moieties caused precipitation of the protein. The complex formed by BpyAla alone with Cu^II^ exhibit very similar behavior with two new absorption bands at 308 and 318 nm (Fig. S4a†). Similar shifts in the UV/Vis spectrum have been reported before for other BpyAla containing proteins.^20^ These UV/Vis absorption bands are attributed to the red shifted π–π* transition of the bipyridine moiety of the incorporated BpyAla upon binding of Cu^II^. Indeed, upon addition of Cu(NO_3_)_2_ to LmrR_LM, *i.e.* the protein without BpyAla incorporated in the structure, no such changes were observed in the UV/Vis absorption spectrum (Fig. S4b†). Comparison of the intensity of these absorption bands of LmrR_LM_M89X_Cu^II^ with those of the isolated complex [Cu(2,2′-bipyridine)(NO_3_)_2_] (Fig. S4c†) suggests that all Cu^II^ ions are bound to the protein. Coordination of Cu^II^ to the LmrR_LM_M89X was further studied by EPR spectroscopy. Cu^II^-BpyAla shows a characteristic EPR spectrum with one perpendicular signal (*g*_⊥_) and four parallel signals (*g*_||_).^38^ The EPR spectrum of LmrR_LM_M89X in the presence of Cu^II^ ions is similar to that of Cu^II^-BpyAla (Fig. S5†). However, all signals were shifted to higher field and a 50% decrease in the intensity of perpendicular signal was observed. Notably, Cu^II^ ions did not show EPR signals at this concentration in the absence of BpyAla or LmrR_LM_M89X.

Characterization of the coordination of Cu^II^ ions to the protein by Raman spectroscopy is facilitated by excitation into the π–π* absorption band (*i.e.* at *λ*_exc_ 355 nm). Addition of Cu^II^ to a solution of BpyAla in MOPS buffer results in the appearance of bands at 1607, 1572, 1505, 1327 and 1029 cm^–1^, which are typical of pyridyl based ligands complexed to metal ions (Fig. 2b(I)).^39^ The resonance Raman spectrum of LmrR_LM_M89X in MOPS buffer shows bands between 1600–1750 cm^–1^ and at *ca.* 1470 cm^–1^ originating from amino acids and the uncomplexed bipyridyl moiety in the protein. Addition of Cu^II^ to LmrR_LM_M89X results in the appearance of additional bands. The position of these bands corresponds with those of Cu^II^-BpyAla (Fig. 2b).

Combined, these spectroscopic studies demonstrate unequivocally binding of Cu^II^ to the bipyridyl moiety of BpyAla in the LmrR_LM_M89X artificial metalloenzyme.

The catalytic activity of the BpyAla containing metalloenzymes was evaluated in the Cu^II^ catalyzed vinylogous Friedel–Crafts alkylation reaction of 5-methoxy-1*H*-indole (**1a**) with 1-(1-methyl-1*H*-imidazol-2-yl)but-2-en-1-one (**2**), resulting in the product **3a**, as the benchmark reaction (Fig. 1a).^40–42^ Notably, this reaction is not catalyzed by the previously reported LmrR-based artificial metalloenzymes, because these did not accept the 2-acyl-1-methylimidazole substrates.^32^ The reaction was carried out using 9 mol% of Cu(NO_3_)_2_ (90 μM), a small excess of 1.25 equivalents of LmrR_LM_X (112.5 μM in monomers) to ensure that all Cu^II^ ions are bound, 1 mM of **1a** and 2.5 equivalents (2.5 mM) of **2** in 3-(N-morpholine)propanesulfonic acid (MOPS) buffer (20 mM, 150 mM NaCl, pH = 7.0) for 3 days at 4 °C. Using Cu(NO_3_)_2_ or Cu(NO_3_)_2_ in combination with LmrR_LM, *i.e.* the protein that does not contain BpyAla, gave rise to conversions of 98% and 64%, respectively, with no significant ee in the latter case (Table 1, entry 1, 2). Also no ee was obtained when Cu(NO_3_)_2_ in combination with l-BpyAla (92% ee)^34^ was used as catalyst (Table S2†), showing that the chiral amino acid itself, in absence of protein scaffold, cannot induce enantioselectivity in the catalyzed reaction. In the absence of Cu^II^, *i.e*. only BpyAla containing protein, no catalytic activity was observed (Table S2†).

**Scheme 1:**
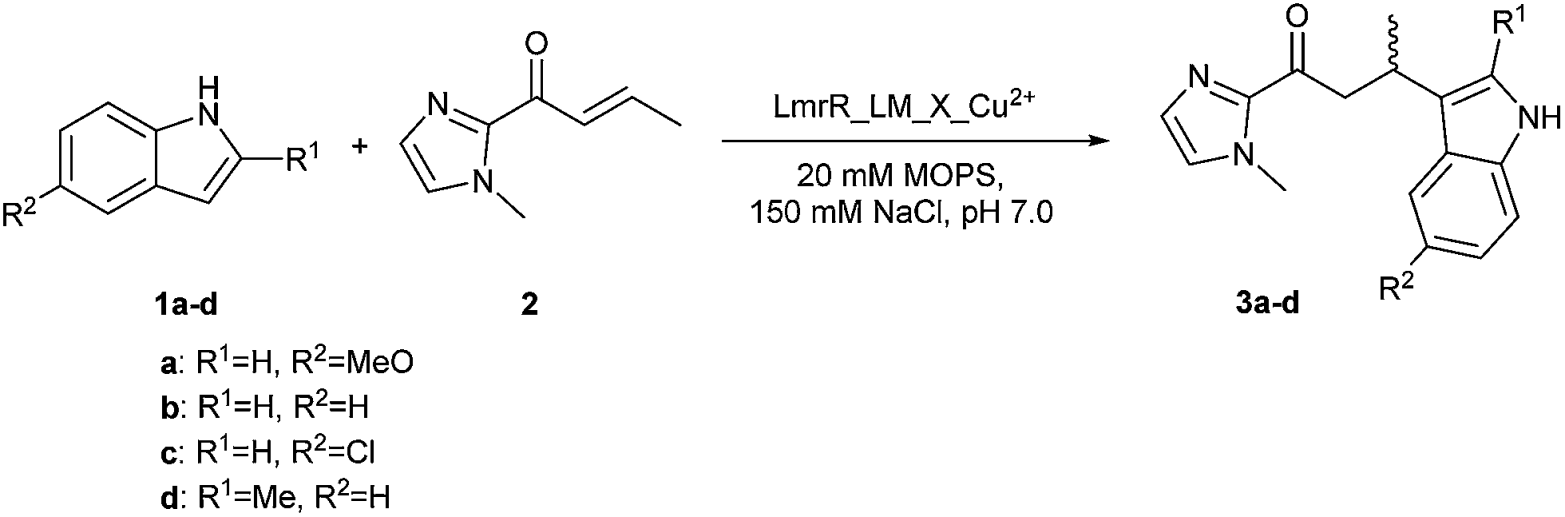
Scope of the catalyzed Friedel–Crafts alkylation reactions.

**Table 1:** Results of the vinylogous Friedel–Crafts reaction of **1a** and **2** resulting in **3a**, catalyzed by LmrR_LM_X_Cu^II^ ^*a*^

Entry	Catalyst	Conversion (%)	ee (%)
1	Cu(NO_3_)_2_	98 ± 2	—
2	LmrR_LM + Cu(NO_3_)_2_	64 ± 9	<5
3	LmrR_LM_N19X_Cu^II^	18 ± 2	29 ± 2 (+)
4	LmrR_LM_M89X_Cu^II^	27 ± 6	49 ± 4 (+)
5	LmrR_LM_F93X_Cu^II^	36 ± 3	22 ± 1 (–)

**Mutagenesis study**			
6	LmrR_LM_M89X_N19A_Cu^II^	49 ± 6	27 ± 6 (+)
7	LmrR_LM_M89X_K22A_Cu^II^	28 ± 3	37 ± 3 (+)
8	LmrR_LM_M89X_H86A_Cu^II^	49 ± 4	51 ± 3 (+)
9	LmrR_LM_M89X_F93A_Cu^II^	20 ± 3	6 ± 3 (+)
10	LmrR_LM_M89X_E107A_Cu^II^	22 ± 1	66 ± 1 (+)
11	LmrR_LM_M89X_H86I_Cu^II^	51 ± 3	43 ± 1 (+)
12	LmrR_LM_M89X_H86W_Cu^II^	36 ± 2	55 ± 1 (+)
13	LmrR_LM_M89X_H86S_Cu^II^	31 ± 4	23 ± 3 (+)
14	LmrR_LM_M89X_H86D_Cu^II^	26 ± 3	39 ± 3 (+)
15	LmrR_LM_M89X_F93I_Cu^II^	7 ± 1	31 ± 1 (+)
16	LmrR_LM_M89X_F93H_Cu^II^	5 ± 1	27 ± 3 (+)
17	LmrR_LM_M89X_F93W_Cu^II^	25 ± 4	53 ± 5 (+)
18	LmrR_LM_M89X_F93D_Cu^II^	43 ± 4	29 ± 6 (–)
19	LmrR_LM_M89X_N19A_E107A_Cu^II^	58 ± 10	14 ± 5 (+)
20	LmrR_LM_M89X_H86A_E107A_Cu^II^	37 ± 1	48 ± 2 (+)

^*a*^Typical conditions: 9 mol% Cu(H_2_O)_6_(NO_3_)_2_ (90 μM) loading with 1.25 eq LmrR_LM_X (in monomer) in 20 mM MOPS buffer (pH 7.0), 150 mM NaCl, for 3 days at 4 °C. All data are the average of 2 independent experiments, each carried out in duplicate.

The three BpyAla containing artificial metalloenzymes gave rise to lower conversions of 18–36% (Table 1, entry 3–5). However, the product **3a** was obtained with encouraging enantioselectivities ranging from 22%, in case of LmrR_LM_F93X (Table 1, entry 5), to 49% with the LmrR_LM_M89X mutant (Table 1, entry 4).

Interestingly, the artificial metalloenzymes containing the BpyAla residue in the interior of the protein structure, *i.e.* LmrR_LM_M89X and LmrR_LM_N19X, showed preference for the formation of the (+) enantiomer of **3a** (Table 1, entry 3, 4), while the (–) enantiomer of **3a** was obtained in excess with LmrR_LM_F93X, which has the BpyAla located on the outside of the hydrophobic pocket of the protein (Table 1, entry 5). Thus, simply by changing the position of the metal binding residue in the scaffold, the chiral microenvironment in which the catalysis takes place is altered such that the preferred stereochemical path of the reaction is inversed. Hence, both enantiomers of the product can be obtained using the same biomolecular scaffold. Reducing the catalyst concentration to 30 μM Cu(NO_3_)_2_/37.5 μM LmrR-LM_X gave rise to similar conversions but a significantly decreased ee (Table S3†).

A mutagenesis study was carried out to explore the role of the biomolecular scaffold in catalysis and to optimize the catalytic performance of the artificial metalloenzyme. LmrR_LM_M89X, with BpyAla incorporated at position 89, was used as a starting point, since this gave the highest enantioselectivities in the reaction of **1a** with **2**.

Based on the X-ray structure of LmrR,^30^ amino acids N19, K22, H86, F93 and E107, which are in spatial proximity to the incorporated BpyAla, were selected (Fig. 1c). In the first round of mutagenesis, an alanine scan was done, that is, the original amino acids at these positions were substituted with alanine to determine the contribution of each of these amino acids in catalysis. All mutants were prepared using standard Quick-Change mutagenesis methods and characterized as described above (ESI†). The catalytic efficiency was evaluated in the Friedel–Crafts alkylation of **2** with **1a** under standard conditions (Table 1, entry 6–10). Indeed, the results showed that all but one of the mutations had a significant effect on the catalytic performance of the artificial metalloenzyme. The only exception was the K22A mutation, which only caused small decrease in ee (Table 1, entry 7). An increase in conversion to 49% was observed with the N19A mutant, albeit that this was accompanied by strong decrease in the ee of **3a** (Table 1, entry 6). The mutants E107A and H86A both gave rise to an increased ee, *i.e.* to 51% and 66%, respectively (Table 1, entry 10, 8). In case of H86A also the conversion was increased significantly compared to LmrR_LM_M89X. Finally, the alanine mutation at position 93 (F93A) caused a dramatic decrease in both enantioselectivity and conversion (Table 1, entry 9).

Based on these results, two residues were selected for a second round of mutagenesis: H86, which resulted in improved catalytic performance after mutation to alanine, and F93, mutagenesis of which had a detrimental effect on catalysis. For both positions, four amino acids that cover a diverse range of chemical properties of the side chains were selected. Histidine 86 was replaced by isoleucine, serine, tryptophan and aspartate. The mutations for the apolar residues isoleucine (H86I) and tryptophan (H86W) were well accepted and resulted in comparable or slightly higher ee and conversion values compared to LmrR_LM_M89X (Table 1, entry 4, 11, 12). In contrast, substitution for the polar residue serine or negatively charged aspartate (H86S, H86D) resulted in a decrease in ee of **3a**, while conversion remained similar (Table 1, entry 13, 14).

Phenylalanine 93 was mutated to isoleucine, histidine, tryptophan and aspartate. The mutation for the apolar and bulky isoleucine (F93I) or the positively charged histidine (F93H) led to a slight decrease in enantioselectivity, *i.e.* 27 and 31%, respectively. However, a dramatic decrease in conversion to 5–7% was found (Table 1, entry 15, 16). Mutation to tryptophan (F93W) had no significant effect on catalysis (Table 1, entry 17). Surprisingly, mutation of F93 for negatively charged aspartate (F93D) resulted in preferential formation of the opposite enantiomer of **3a**, *i.e.* 29% ee of the (–) enantiomer. Finally, two double mutants, *i.e.* LmrR_LM_M89X_N19A_E107A and LmrR_LM_M89X_H86A_E107A were prepared. Unfortunately, the combination of two mutations that individually had a positive effect on catalysis proved not to be synergistic and no significant improvement of the results of catalysis was observed (Table 1, entry 19, 20).

Combined, the data from mutagenesis studies clearly show the importance for catalysis of the second coordination sphere provided by the biomolecular scaffold. Furthermore, the results from the alanine scanning strongly support that the catalysis takes place inside the hydrophobic pore provided by LmrR in the immediate vicinity of the metal binding residue BpyAla. In particular H86 and F93 appeared to be important positions, since mutation to alanine had a positive and strongly negative effect, respectively, on both conversion and enantioselectivity. The second round of mutagenesis of position 86 suggests that, although the differences in conversion and enantioselectivity are not large, apolar amino acids are preferred over polar residues at this position for the benchmark Friedel–Crafts reaction. The bulkiness of the side-chain appeared to be not particularly important since similar results were obtained with alanine, isoleucine and tryptophan at this position.

The results of mutagenesis at position 93 strongly suggest the necessity of an aromatic side-chain in order to achieve both good conversions and ee's. The reason for this strong preference is not clear, a possible hypothesis is that the π-stacking interactions are important in binding and orienting the substrates. Alternatively, this residue may play a more general role in retaining the open structure of the hydrophobic pore of the protein, which serves as the active site where catalysis takes place. Further structural studies are needed to establish the role of F93 in catalysis.

The substrate scope of the LmrR_LM_M89X catalyzed Friedel–Crafts reaction was investigated using indole derivatives **1b–1d** as substrate (Scheme 1, Table 2). For this study three LmrR mutants were selected: LmrR_LM_M89X, LmrR_LM_M89X_H86A and LmrR_LM_M89X_F93W, which gave rise to some of the best results in the benchmark reaction of **1a** with **2**. Using substrate **1b**, lower conversions and moderate ee's in the range of 49–55% were observed with all mutants (Table 2, entry 1–3). 5-Chloro indole (**1c**) proved to be a poor substrate for the present artificial metalloenzymes: only low ee's and hardly any conversion was obtained (Table 2, entry 4–6). Surprisingly, using 2-methyl indole (**1d**), high conversions and ee's ranging from 68–83%, were obtained (Table 2, entry 7–9). Both conversion and ee in this case are significantly higher than those achieved with other indoles. Apparently, the 2-methylindole substrate is best compatible with the chiral microenvironment provided by the hydrophobic pocket of LmrR. While such substrate specificity is not attractive from a catalysis perspective, where broad scope is desired, it is reminiscent of natural enzymes that have been evolved for one specific substrate only. Finally, a noticeable trend is observed in the conversion and ee's achieved with various mutants. Of the three mutants, LmrR_LM_M89X_F93W consistently gave rise to the highest ee's in the catalyzed reaction.

**Table 2:** Scope of the vinylogous Friedel–Crafts alkylation reaction catalyzed by LmrR_LM_X_Cu^II^ ^*a*^

Entry	Catalyst	Substrate	Product	Conversion (%)	ee (%)
1	LmrR_LM_M89X_Cu^II^	**1b**	**3b**	16 ± 6	52 ± 3 (+)
2	LmrR_LM_M89X_H86A_Cu^II^	**1b**	**3b**	11 ± 0	49 ± 3 (+)
3	LmrR_LM_M89X_F93W_Cu^II^	**1b**	**3b**	16 ± 6	55 ± 3 (+)
4	LmrR_LM_M89X_Cu^II^	**1c**	**3c**	2 ± 1	21 ± 1 (+)
5	LmrR_LM_M89X_H86A_Cu^II^	**1c**	**3c**	5 ± 0	29 ± 0 (+)
6	LmrR_LM_M89X_F93W_Cu^II^	**1c**	**3c**	3 ± 1	50 ± 2 (+)
7	LmrR_LM_M89X_Cu^II^	**1d**	**3d**	92 ± 4	80 ± 2 (+)
8	LmrR_LM_M89X_H86A_Cu^II^	**1d**	**3d**	79 ± 2	68 ± 2 (+)
9	LmrR_LM_M89X_F93W_Cu^II^	**1d**	**3d**	94 ± 8	83 ± 0 (+)

^*a*^Typical conditions: 9 mol% Cu(H_2_O)_6_(NO_3_)_2_ (90 μM) loading with 1.25 eq. LmrR_LM_X (in monomer) in 20 mM MOPS buffer (pH 7.0), 150 mM NaCl, for 3 days at 4 °C. All data are the average of 2 independent experiments, each carried out in duplicate.

## Conclusions

In conclusion, we have introduced a new strategy for preparation of artificial metalloenzymes comprising *in vivo* incorporation of a metal-binding non-proteinogenic amino acid BpyAla into the protein LmrR, using the amber stop codon suppression methodology. To the best of our knowledge, this represents the first example of an artificial metalloenzyme with an *in vivo* incorporated unnatural amino acid capable of binding a transition metal ion catalyzing an enantioselective reaction. A particularly attractive feature of this approach is that it allows for rapid optimization of artificial metalloenzymes by genetic methods, which was demonstrated by the preparation of several mutants of this novel artificial metalloenzyme. Up to 83% ee for the product was achieved in the catalytic asymmetric vinylogous Friedel–Crafts alkylation reaction. Interestingly, this is a reaction that was not catalyzed by the previously reported LmrR-based artificial metalloenzymes involving covalent anchoring of the metal complex *via* an introduced cysteine. This suggests that the structure of the active site of the artificial metalloenzymes presented here is significantly different, resulting in novel catalytic activity. Due to its versatility, it is envisioned that this novel design approach to allow for directed evolution of artificial metalloenzymes, in particular for novel reactions that require 2^nd^ coordination sphere interactions,^2^ *e.g.* catalytic asymmetric hydration reactions,^32^ and/or their application in living cells, ultimately making it possible to augment cellular biosynthesis with unnatural catalytic transformations.
