# Supplementary material for: Novel artificial metalloenzymes by in vivo incorporation of metal-binding unnatural amino acids
Source: Chem Sci. 2014 Oct 9;6(1):770–6. doi: 10.1039/c4sc01525h (PMC5590542; doi:10.1039/c4sc01525h)
Supplement: Supplementary file 1 [file SC-006-C4SC01525H-s001.pdf]

Electronic Supplementary Material (ESI) for Chemical Science.
This journal is © The Royal Society of Chemistry 2014

Novel artificial metalloenzymes by *in-vivo* incorporation of metal-binding unnatural amino acids

Ivana Drienovská, Ana Rioz-Martínez, Apparao Draksharapu and Gerard Roelfes*

[a]*Stratingh Institute for Chemistry, University of Groningen, Nijenborgh 4, 9747 AG Groningen, The Netherlands.*

e-mail: *j.g.roelfes@rug.nl*

Supporting Information

Table of Contents

1. General remarks *S2*

2. Experimental procedures and characterization data of compounds *S3*

3. Molecular Biology *S7*

4. Expression and purification *S9*

5. SDS-PAGE *S10*

6. Analytical size-exclusion chromatography *S13*

7. ESI-mass spectra *S21*

8. Characterization of the complex Cu^{II}- BpyAla formation *S39*

9. Catalysis – Friedel-Crafts reaction *S42*

10. References *S47*

1. General remarks

Chemicals were purchased from Sigma Aldrich or Acros and used without further purification. H-NMR and C-NMR spectra were recorded on a Varian 400 (400 and 100 MHz) in $CDCl_3$ or DMSO-d6. Mass spectra (HR-MS) were recorded on an Orbitrap XL (Thermo Fisher Scientific; ESI pos. mode). Enantiomeric excess determinations were performed by HPLC analysis (Chiralpak-AD column) using UV-detection (Shimadzu SCL-10Avp).

E.*coli* strains XL-1-Blue and BL21 (DE3)_C43 (Stratagene) were used for cloning and expression. DNA sequencing was carried out by GATC-Biotech (Berlin, Germany). Primers were synthesized by Eurofins MWG Operon (Ebersberg, Germany). Restriction endonucleases were purchased from New England Biolabs. T4 DNA ligase, DNA Gel Extraction Kit and Plasmid Purifying Kit were purchased from Roche. *Pfu* Turbo polymerase was purchased from Stratagene. FPLC columns were purchased from GE Healthcare.

2. Experimental procedures and characterization data of compounds.

2.1. Synthesis of methyl 2,2′- bipyridine-5-carboxylate.

1.5 M *t*-BuLi in *n*-pentane (40.5 mmol, 2.15 eq.) was added to THF (100 mL) at -78 °C under N_2 atmosphere. To this pale-yellow solution, 2-bromopyridine (22.9 mmol, 1.20 eq.) was slowly added via syringe and stirred for 30 min at the same temperature. After that time a solution of $ZnCl_2$ (10.5 mmol, 2.75 eq.) in THF (80 mL) was added. The resulting solution was allowed to warm to room temperature and stirred for 2.5 h (solution 1).

Methyl 6-chloronicotinate (19.1 mmol, 1.0 eq.) and $Pd(PPh_3)_4$ (574 nmol, 3 mol%) were dissolved in THF (20 mL) under N_2 atmosphere (solution 2). The corresponding solution was slowly added to solution 1 and the resulting mixture was stirred at reflux for 18 h. The reaction was followed via TLC. After total consumption of the starting material, the mixture was quenched with a saturated aqueous solution of EDTA (120 mL) and stirred at room temperature for 15 min. Once finished, a saturated aqueous solution of Na_2CO_3 was added until pH 8. The product was extracted with CH_2Cl_2 (3 × 50 mL) and the organic layer was dried over Na_2SO_4, filtered and the solvent evaporated under reduced pressure. The crude residue was purified by flash chromatography on silica gel with heptane/ethyl acetate (3:1) + 5% Et_3N to afford methyl 2,2′- bipyridine-5-carboxylate (3.84 g, 94% yield) as a white solid. Analytical data were in accordance with those previously published.[1]

^{1}H-NMR ($CDCl_3$-d_1, 400 MHz): δ 3.98 (s, 3H), 7.26-7.38 (m, 1H), 7.85 (t, 1H, $^3J_{HH}$ 8.0 Hz), 8.41 (d, 1H, $^3J_{HH}$ 8.0 Hz), 8.48-8.51 (m, 2H), 8.70 (d, 1H, $^3J_{HH}$ 4.0 Hz) and 9.27 (s, 1H).

2.2. Synthesis of 5-(bromomethyl) 2,2′- bipyridine.

To a solution of methyl 2,2′- bipyridine-5-carboxylate (17.9 mmol, 1.0 eq.) in anhydrous THF (83 mL) at 0 ºC was added lithium borohydride (89.5 mmol, 5.0 eq.). The reaction mixture was stirred at this temperature for 16 hours and then quenched slowly with 166 mL of water. After the evaporation of THF under reduced pressure, the product was extracted with CH_2Cl_2 (3 × 50 mL) and the organic layer was dried over Na_2SO_4 and filtered. The solution was then concentrated under reduced pressure to afford 3.27 g of 5-(hydroxymethyl)-2,2'-bipyridine as a thick orange oil which was used in the subsequent reaction without purification.

The product from the preceding reaction was dissolved in CH_2Cl_2 (40 mL) and cooled to 0 ºC. To this solution, tetrabromomethane (20.3 mmol, 1.13 eq.) and triphenylphosphine (20.3 mmol, 1.13 eq.) were slowly added. After stirring for 16 hours, the reaction mixture was concentrated to approximately one quarter of the original volume and applied directly to a flash silica gel column (eluent: heptane/Et_2O (1:l)). Concentration of the pure fractions under reduced pressure provided 2.7 g (50% yield over two steps) of 5-(bromomethyl)-2,2'-bipyridine as a pale yellow solid. Analytical data were in accordance with those previously published.[2]

^{1}H-NMR ($CDCl_3$-d_1, 400 MHz): δ 4.54 (s, 2H), 7.26-7.34 (m, 1H), 7.80-7.84 (m, 2H), 8.40 (d, 2H, $^3J_{HH}$ 8.0 Hz) and 8.60 (s, 2H).

2.3. Synthesis of (2,2′-bipyridin-5-yl)alanine.

To a solution of diethyl acetaminomalonate (13.4 mmol, 1.5 eq.) and NaOEt (13.4 mmol, 1.5 eq.) in EtOH (80 mL, anhydrous), 5-(bromomethyl) 2,2′- bipyridine (9.0 mmol, 1.0 eq.) was added under an N_2 atmosphere. The reaction mixture was heated to reflux overnight. The solvent was evaporated under reduced pressure and the residue was purified by silica gel flash column chromatography heptane/EtOAc 1:1 (until isolation of the compound that corresponds to the first spot in the TLC) and then CH_2Cl_2/MeOH 9:1 to give diethyl 2-(2,2′-bipyridin-5-ylmethyl)-2-acetamidomalonate as a white solid (3.5 g, 70%).[3]

^{1}H-NMR ($CDCl_3$-d_1, 400 MHz): δ 1.30 (t, 6H, $^3J_{HH}$ 8.0 Hz), 2.06 (s, 3H), 3.73 (s, 2H), 4.19-4.21 (c, 4H, $^3J_{HH}$ 8.0 Hz), 6.60 (s, 1H), 7.26-7.30 (m, 1H), 7.47 (d, 1H, $^3J_{HH}$ 8.0 Hz), 7.78 (t, 1H, $^3J_{HH}$ 8.0 Hz), 8.28-8.34 (m, 3H), 8.66 (d, 1H, $^3J_{HH}$ 8.0 Hz).

Finally, a suspension of diethyl 2-(2,2′-bipyridin-5-ylmethyl)-2-acetamidomalonate (9.0 mmol) in aqueous HCl (60 mL, 37% in water) was heated to reflux overnight. The solvent was evaporated under reduced pressure to give bipyridylalanine as a white HCl salt (2.6 g, 82%), which was used in experiments without further purification. Analytical data were in accordance with those previously published.[3]

^{1}H-NMR (MeOD-d_1, 400 MHz): δ 3.49 (dd, 1H, $^3J_{HH}$ 8.0 Hz, $^2J_{HH}$ 16.0 Hz), 3.57 (dd, 1H, $^3J_{HH}$ 8.0 Hz, $^2J_{HH}$ 16.0 Hz), 4.52 (t, 1H, $^3J_{HH}$ 8.0 Hz), 8.09 (t, 1H, $^3J_{HH}$ 8.0 Hz), 8.32 (dd, 1H, $^3J_{HH}$ 8.0 Hz, $^4J_{HH}$ 4.0 Hz), 8.62 (d, 1H, $^3J_{HH}$ 8.0 Hz), 8.67 (dt, 1H, $^3J_{HH}$ 8.0 Hz, $^4J_{HH}$ 4.0 Hz), 8.79 (d, 1H, $^3J_{HH}$ 8.0 Hz) and 8.94-8.97 (m, 2H).

^{13}C-NMR (CD_3OD-d_4, 75.4 MHz): δ 34.1, 54.2, 124.4 , 125.2, 128.4, 136.6, 143.3, 145.5, 146.6, 147.3, 148.8, 150.2 and 170.6. HRMS (ESI^+) calcd for $C_{13}H_{14}N_3O_2$ ($M+H^+$): 244.108; found: 244.107.

Scheme S1

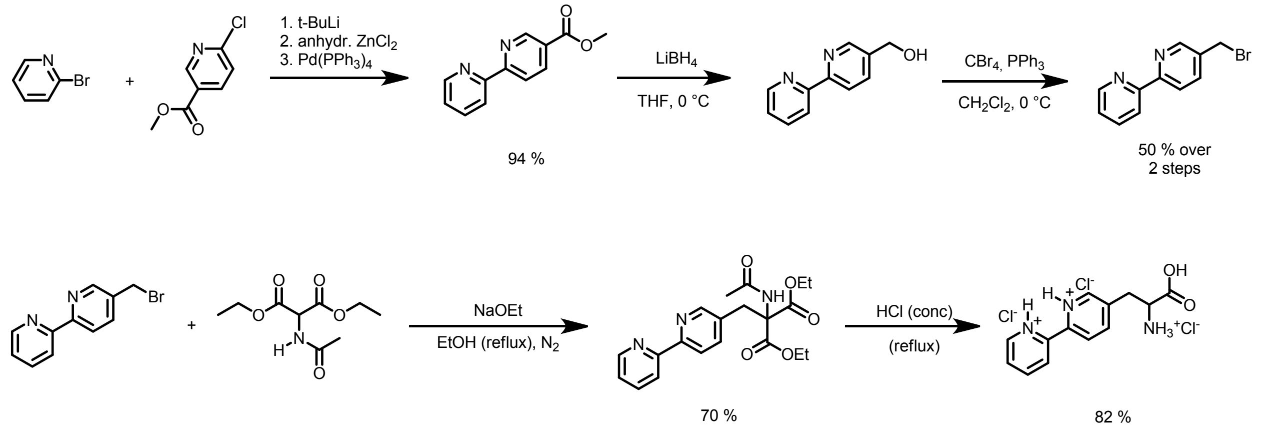

^{1}H-NMR

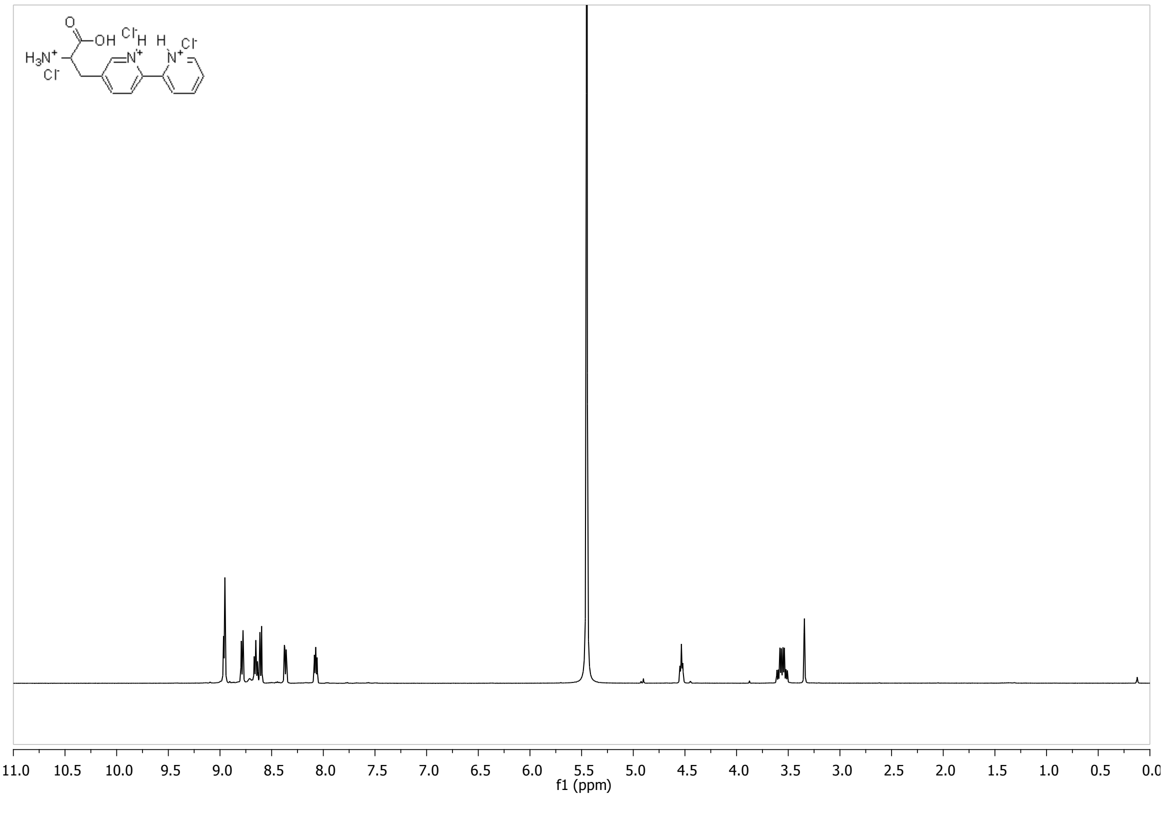

3. Molecular Biology

Gene optimization:

The synthesized gene of LmrR was ordered from GenScript (USA). The codon usage was adapted to the codon bias of *E. coli* genes. The gene was delivered in the cloning vector pUC57, containing a C-terminal strep-tag and K55D, K55Q mutations as previously described.[4] The gene was subsequently recloned to the pET17b expression plasmid using the restriction sites *NdeI* and *HindIII.*

Optimized sequence:

ATGGGTGCCGAAATCCCGAAAGAAATGCTGCGTGCTCAAACCAATGTCATCCTGCTGAATGTCCTGAAACAAGGCGATAACTATGT
GTATGGCATTATCAAACAGGTGAAAGAAGCGAGCAACGGTGAAATGGAACTGAATGAAGCCACCCTGTATACGATTTTTGATCGTC
TGGAACAGGACGGCATTATCAGCTCTTACTGGGGTGATGAAAGTCAAGGCGGTCGTCGCAAATATTACCGTCTGACCGAAATCGGC
CATGAAAACATGCGCCTGGCGTTCGAATCCTGGAGTCGTGTGGACAAAATCATTGAAAATCTGGAAGCAAACAAAAAATCTGAAGC
GATCAAATCTAGAGGTGGCAGCGGTGGCTGGAGCCACCCGCAGTTCGAAAAATAA

Site directed mutagenesis:

Site-directed mutagenesis was used for preparation of all LmrR mutants. The primers required for the mutagenesis are summarized in **Table S1**. The following PCR cycles were used: initial denaturation at 95 ^{0}C for 1 min, denaturation at 95 ^{0}C for 30 s, annealing at 58-63 ^{0}C for 1 min (depending on the T_m of the particular mutant) and extension at 68 ^{0}C for 5 min. The thermal cycle was repeated 16 times. The resulting PCR product was digested with restriction endonuclease DpnI for 1h at 37 ^{0}C and transformed into the *E.Coli* XL1-blue cells.

Table S1: Primers used for site-directed mutagenesis

**Primer**	**Sequence (5' → 3')**
LmrR_LM_**N19X_fw**	GTC ATC CTG CTG TAG GTC CTG AAA CAA G
LmrR_LM_**N19X_rv**	CTT GTT TCA GGA CCT ACA GCA GGA TGA C
LmrR_LM_**M89X_ fw**	GGC CAT GAA AAC TAG CGC CTG GCG TTC
LmrR_LM_**M89X_ rv**	GAA CGC CAG GCG CTA GTT TTC ATG GCC
LmrR_LM_**F93X_ fw**	ATG CGC CTG GCG TAG GAA TCC TGG AGT
LmrR_LM_**F93X_ rv**	ACT CCA GGA TTC CTA CGC CAG GCG CAT
LmrR_LM_**M89X_N19A_ fw**	GTC ATC CTG CTG GCG GTC CTG AAA CAA
LmrR_LM_**M89X_N19A_ rv**	TTG TTT CAG GAC CGC CAG CAG GAT GAC
LmrR_LM_**M89X_K22A_ fw**	CTG AAT GTC CTG GCG CAA GGC GAT AAC
LmrR_LM_**M89X_K22A_ rv**	GTT ATC GCC TTG CGC CAG GAC ATT CAG
LmrR_LM_**M89X_H86A_ fw**	ACC GAA ATC GGC GCG GAA AAC TAG CGC
LmrR_LM_**M89X_H86A_ rv**	GCG CTA GTT TTC CGC GCC GAT TTC GGT
LmrR_LM_**M89X_F93A_ fw**	TAG CGC CTG GCG GCA GAA TCC TGG AGT
LmrR_LM_**M89X_F93A_ rv**	ACT CCA GGA TTC TGC CGC CAG GCG CTA
LmrR_LM_**M89X_E107A_ fw**	ATT GAA AAT CTG GCG GCA AAC AAA AAA
LmrR_LM_**M89X_E107A_ rv**	TTT TTT GTT TGC CGC CAG ATT TTC AAT
LmrR_LM_**M89X_H86I_ fw**	ACC GAA ATC GGC ATC GAA AAC TAG CGC
LmrR_LM_**M89X_H86I_ rv**	GCG CTA GTT TTC GAT GCC GAT TTC GGT
LmrR_LM_**M89X_H86W_ fw**	ACC GAA ATC GGC TGG GAA AAC TAG CGC
LmrR_LM_**M89X_H86W_ rv**	GCG CTA GTT TTC CCA GCC GAT TTC GGT
LmrR_LM_**M89X_H86S_ fw**	ACC GAA ATC GGC GCG GAA AAC TAG CGC
LmrR_LM_**M89X_H86S_ rv**	GCG CTA GTT TTC CGC GCC GAT TTC GGT
LmrR_LM_**M89X_H86D_ fw**	ACC GAA ATC GGC GCG GAA AAC TAG CGC
LmrR_LM_**M89X_H86D_ rv**	GCG CTA GTT TTC CGC GCC GAT TTC GGT
LmrR_LM_**M89X_F93I_ fw**	TAG CGC CTG GCG ATC GAA TCC TGG AGT
LmrR_LM_**M89X_F93I_ rv**	ACT CCA GGA TTC GAT CGC CAG GCG CTA
LmrR_LM_**M89X_F93H_ fw**	TAG CGC CTG GCG CAT GAA TCC TGG AGT
LmrR_LM_**M89X_F93H_ rv**	ACT CCA GGA TTC ATG CGC CAG GCG CTA
LmrR_LM_**M89X_F93W_ fw**	TAG CGC CTG GCG TGG GAA TCC TGG AGT
LmrR_LM_**M89X_F93W_ rv**	ACT CCA GGA TTC CCA CGC CAG GCG CTA
LmrR_LM_**M89X_F93D_ fw**	TAG CGC CTG GCG GAT GAA TCC TGG AGT
LmrR_LM_**M89X_F93D_ rv**	ACT CCA GGA TTC ATC CGC CAG GCG CTA

4. Expression and purification

The plasmids pEVOL-BpyA and pET17b_LmrR_LM_X were cotransformed into *E. coli* BL21 (DE3)_C43 and a single colony was used to inoculate an overnight culture of 10 mL of fresh LB medium containing 100 µg/mL of ampicillin and 34 µg/ml of chloramphenicol. 2 mL (500x dilutions) of overnight culture was used to inoculate 1 L of fresh LB medium containing 100 µg/mL of ampicillin 34 µg/ml of chloramphenicol. When the culture reached an optical density at 600 nm of 0.8–0.9, the expression was induced with isopropyl β-D-1-thiogalactopyranoside (IPTG) (final concentration 1 mM) and L-Arabinose (final concentration 0.02%). Expression was done overnight at 30 °C. Cells were harvested by centrifugation (6000 rpm, JA10, 20 min, 4 °C, Beckman), resuspended in washing buffer (50 mM NaH_2PO_4, 150 mM NaCl, 10% glycerol. pH 8.0) and sonicated (75% (200W) for 5 min (10 sec on, 15 sec off). The lysed cells were incubated with DNAseI (final concentration 0.1 mg/mL with 10 mM $MgCl_2$) and PMSF solution (final concentration 0.1 mM) for 1 hour at 30 °C. After centrifugation (15000 rpm, JA-17, 1h, 4 °C, Beckman), the supernatant was loaded on a Strep-Tactin column and incubated for 1 h. The column was washed with 3 x 1 CV (column volume) of resuspension buffer (same as wash buffer used before), and eluted with 6 x 0.5 CV of resuspension buffer containing 2.5 mM desthiobiotin. The fractions were analyzed on a 12% polyacrylamide SDS-Tris Tricine gel followed by Coomassie staining. The concentration of the proteins was determined by using the calculated extinction coefficient ε_{280} = 25440 M^{-1} cm^{-1} (F93W mutant: ε_{280} = 30940 M^{-1} cm^{-1}) and corrected for the absorbance of the BpyAla. The correction factor for protein with BpyAla was determined by a standard Bradford assay using 'wildtype' LmrR as standard. Expression yields were 6-18 mg/L. In order to use proteins in the catalysis, they were dialysed against MOPS buffer (20 mM MOPS, 150 NaCl, pH=7.0) overnight at 4 °C. Two mutants, i.e. LmrR_LM_F93X and LmrR_LM_M89X_F93D, were incubated with 500 mM EDTA for 4 hours prior to dialysis.

5. SDS-PAGE

Figure S1: SDS-PAGE gels of the LmrR mutants after Strep-Tag purification.

LmrR_LM_**N19X**

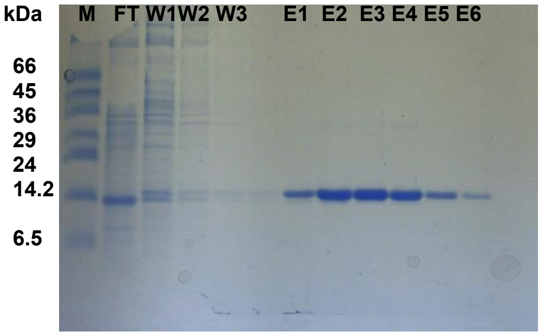

LmrR_LM_**M89X**

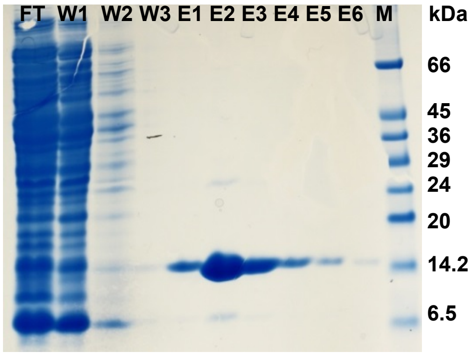

LmrR_LM_**F93X**

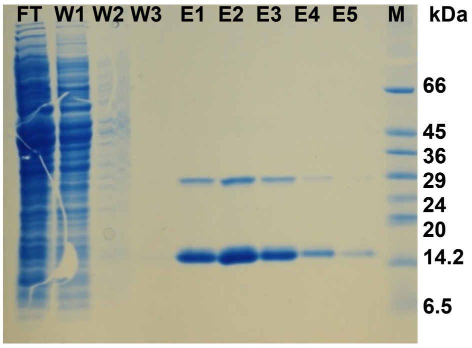

LmrR_LM_**M89X_N19A**

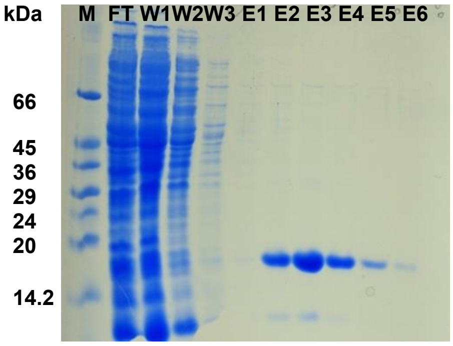

LmrR_LM_M89X _K22A

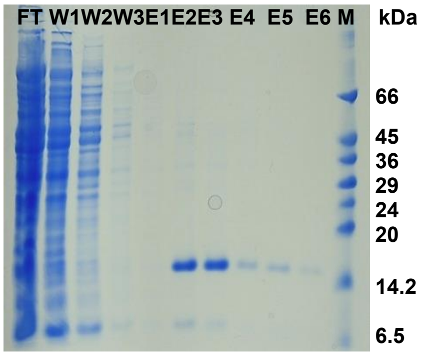

LmrR_LM_**M89X _H86A**

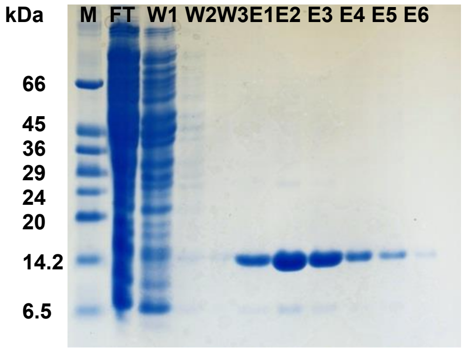

LmrR_LM_M89X _F93A

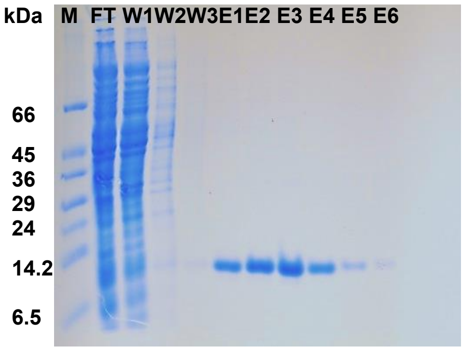

LmrR_LM_**M89X _E107A**

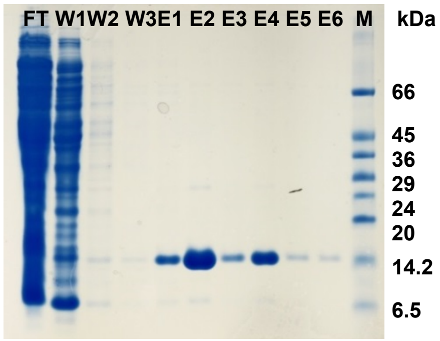

LmrR_LM_**M89X_H86I**

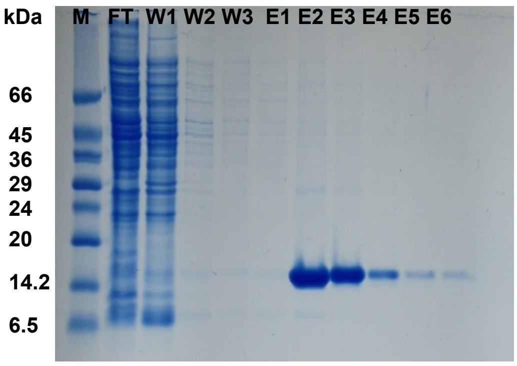

LmrR_LM_**M89X_H86S**

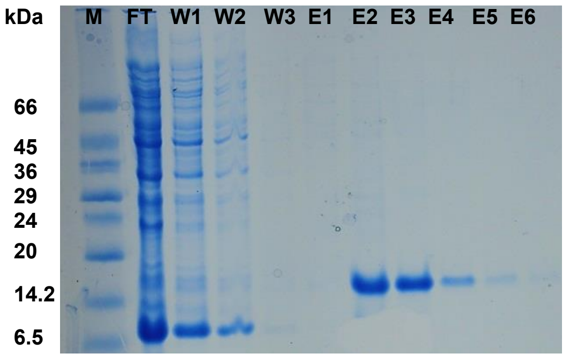

LmrR_LM_**M89X_H86W**

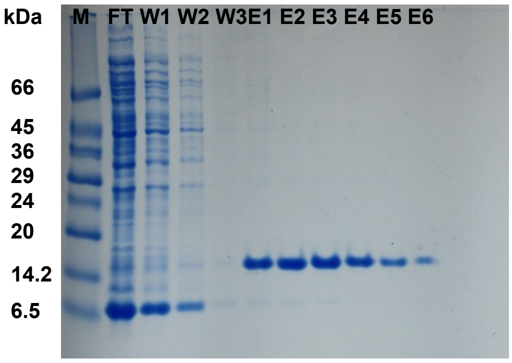

LmrR_LM_**M89X_H86D**

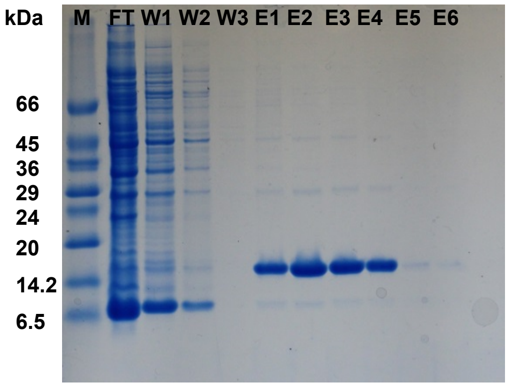

LmrR_LM_**M89X_F93I**

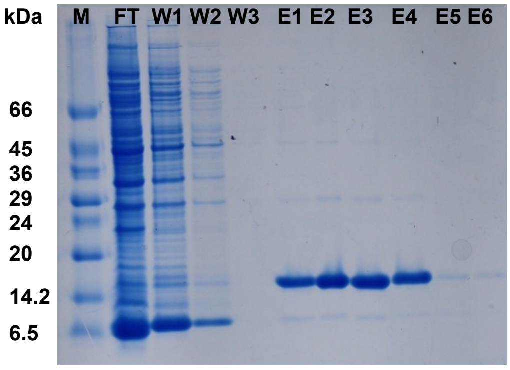

LmrR_LM_**M89X_F93H**

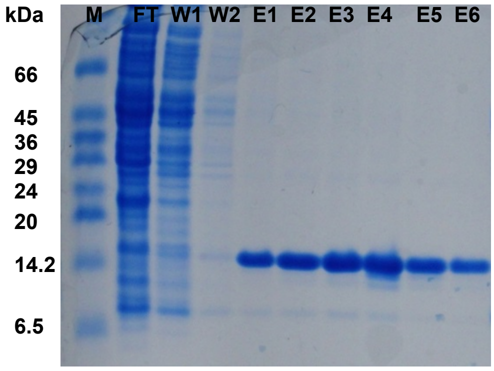

LmrR_LM_**M89X_F93D**

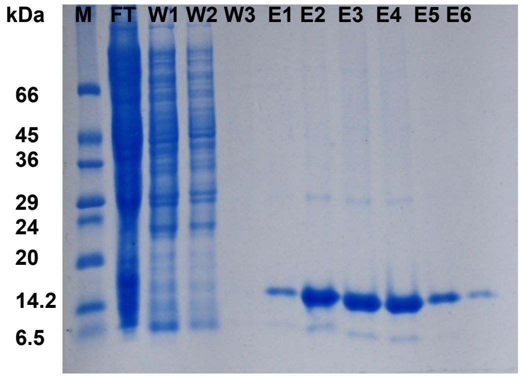

LmrR_LM_**M89X_F93W**

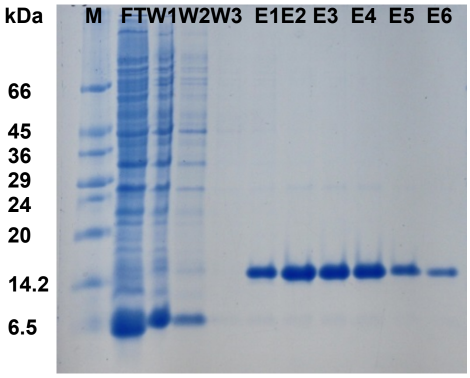

LmrR_LM_**M89X_N19A_E107A**

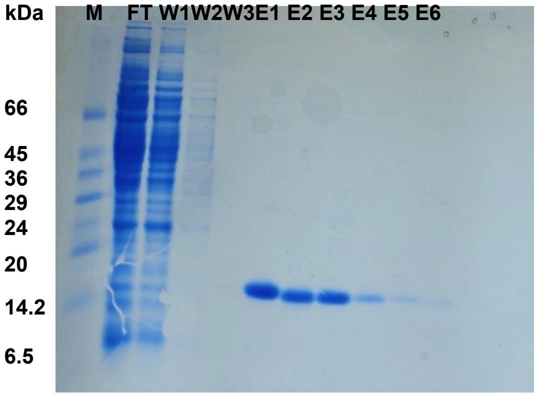

LmrR_LM_**H86A_E107A**

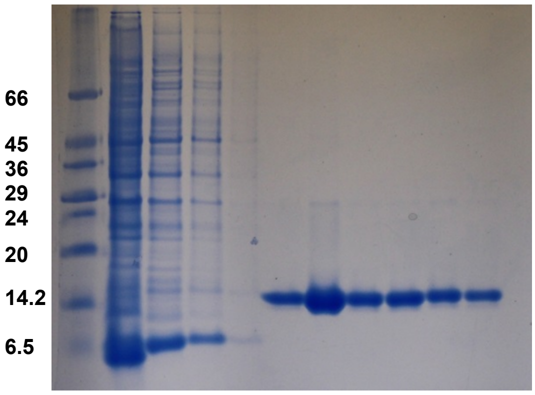

Legend:

M- Marker (SigmaMarker™ low range, mol wt 6500-66000 Da), FT - Flowthrough column, W - wash fraction, E - elution fraction.

Gels were stained with InstantBlue™ (Expedeon).

6. Analytical size-exclusion chromatography

General procedure

Analytical size exclusion chromatography was performed on a Superdex 75 10/300 GL (GE Healthcare). 100 µL of the sample was injected using 20 mM MOPS, 150 mM NaCl pH 7.0, as buffer (flow 0.5 mL/min). The column was calibrated using the standard Gel Filtration LMW Calibration Kit of GE Healthcare. The mutants LmrR_LM_N19X/M89X/F93X were also tested in presence of $Cu(NO_3)_2$ (90 µM concentration – i.e. conditions of catalysis, 1:1.25 ratio)

Figure S2: Analytical size-exclusion chromatography (Superdex-75 10/300 GL) of LmrR and LmrR mutants.

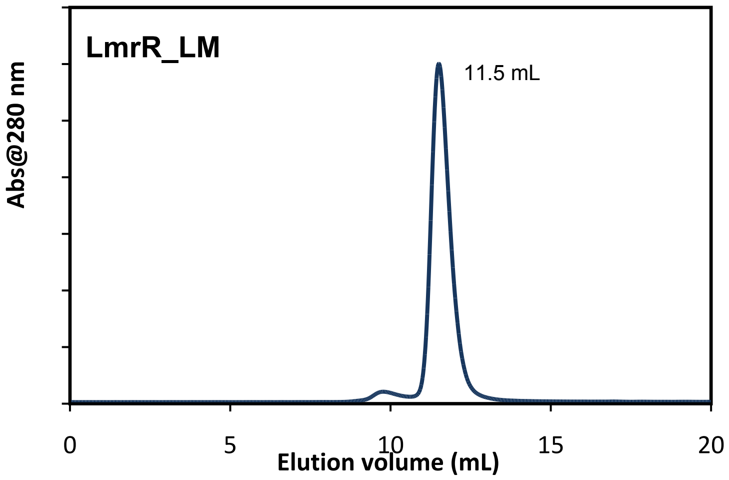

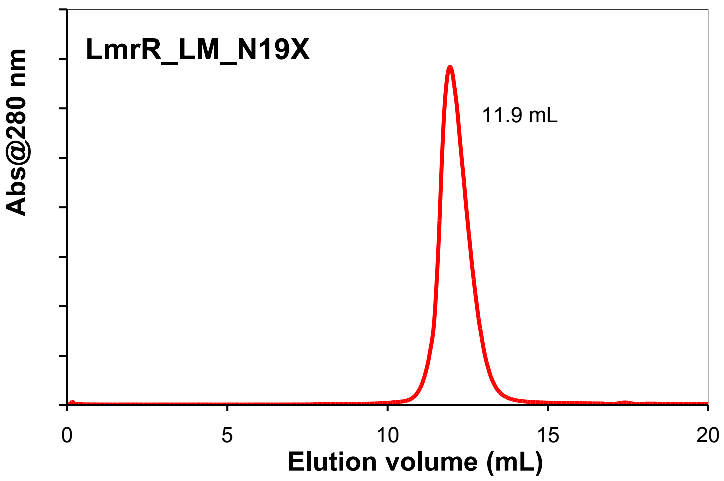

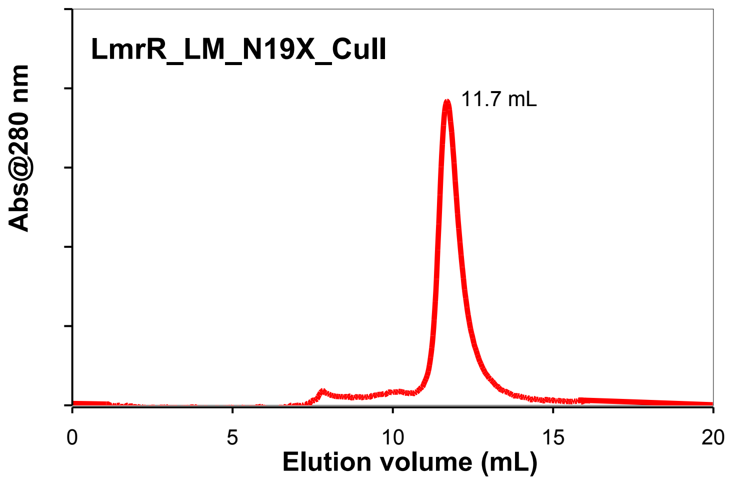
LmrR_LM_N19X_CuII
11.7 mL
Abs@280 nm
0
5
10
15
20
Elution volume (mL)

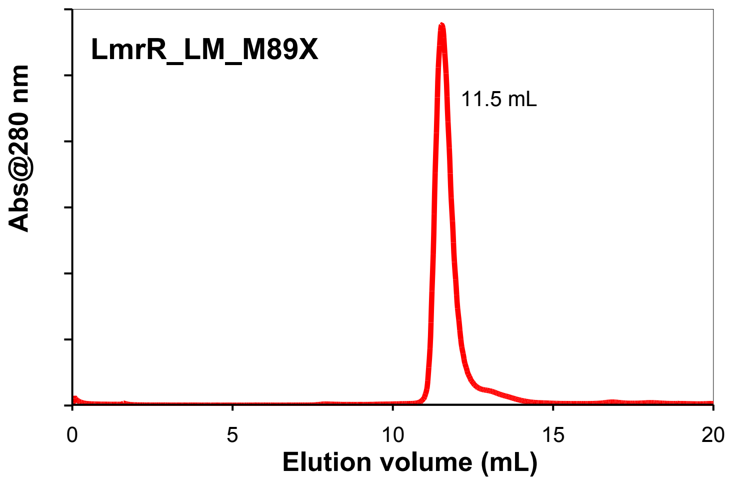
LmrR_LM_M89X
11.5 mL
Abs@280 nm
0
5
10
15
20
Elution volume (mL)

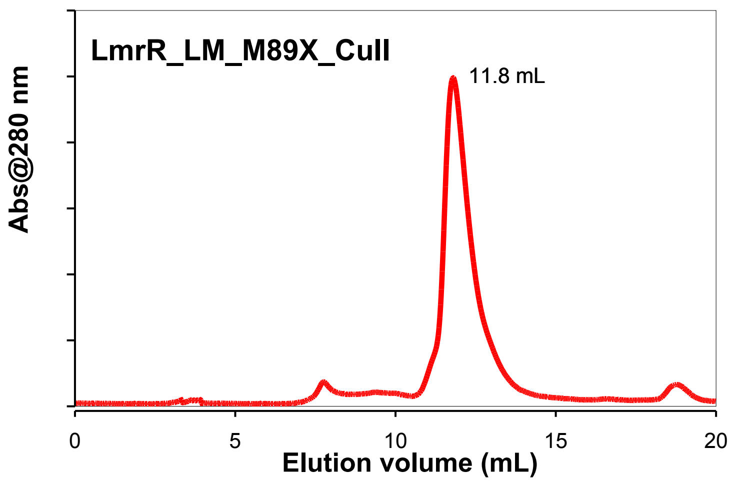
LmrR_LM_M89X_CuII
11.8 mL
Abs@280 nm
0
5
10
15
20
Elution volume (mL)

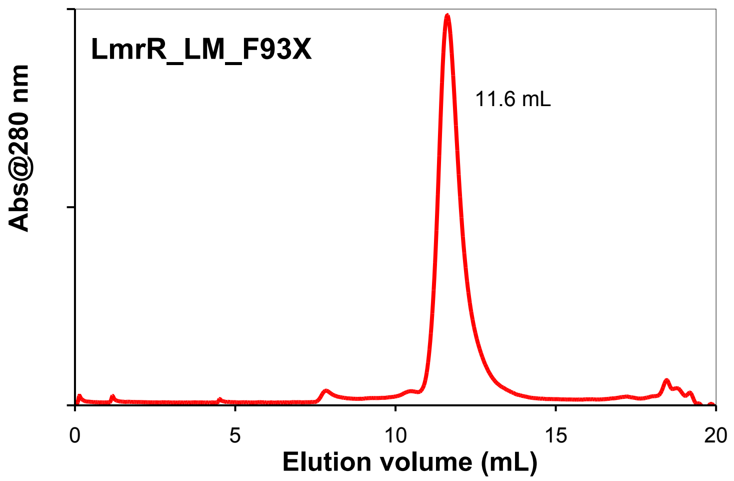
LmrR_LM_F93X
11.6 mL
Abs@280 nm
0
5
10
15
20
Elution volume (mL)

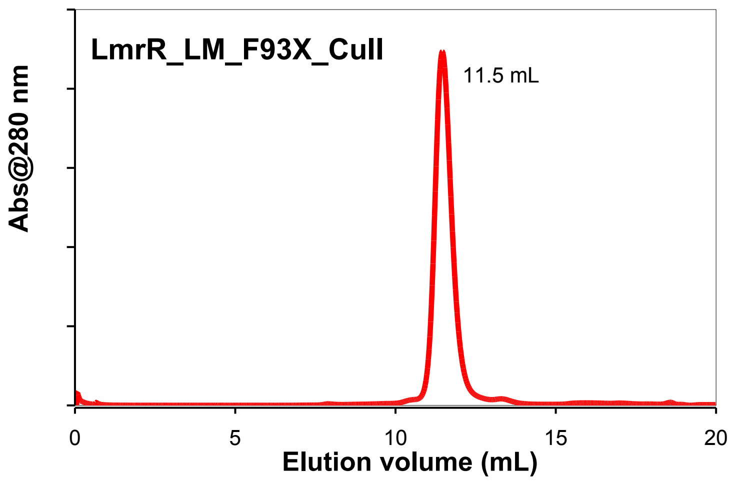
LmrR_LM_F93X_CuII
11.5 mL
Abs@280 nm
0
5
10
15
20
Elution volume (mL)

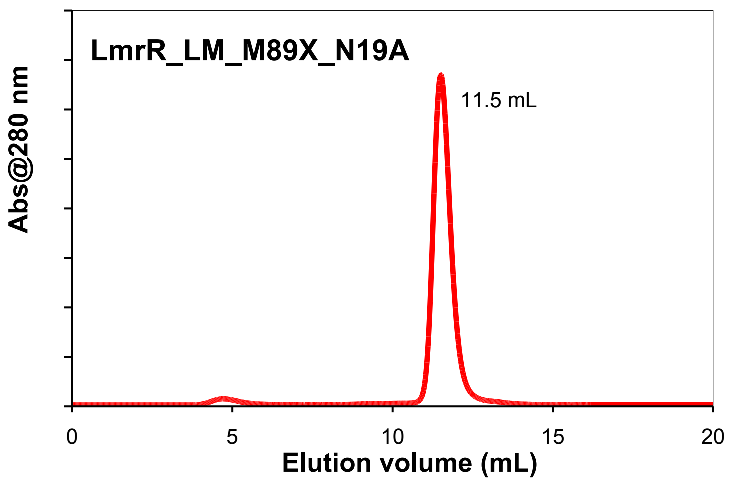
LmrR_LM_M89X_N19A
11.5 mL
Abs@280 nm
0
5
10
15
20
Elution volume (mL)

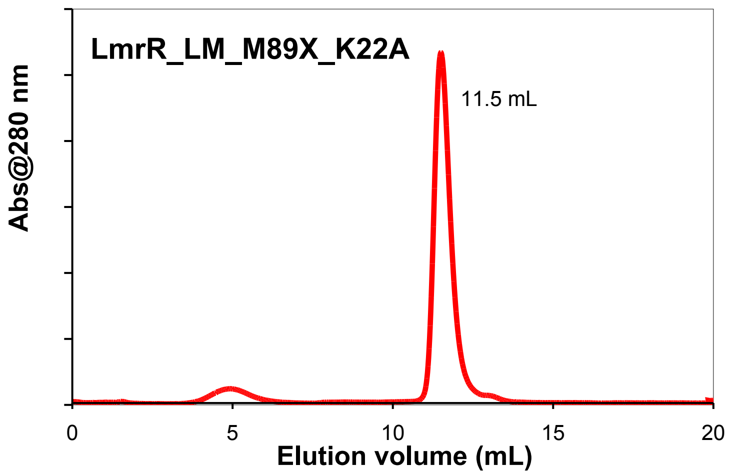
LmrR_LM_M89X_K22A
11.5 mL
Abs@280 nm
0
5
10
15
20
Elution volume (mL)

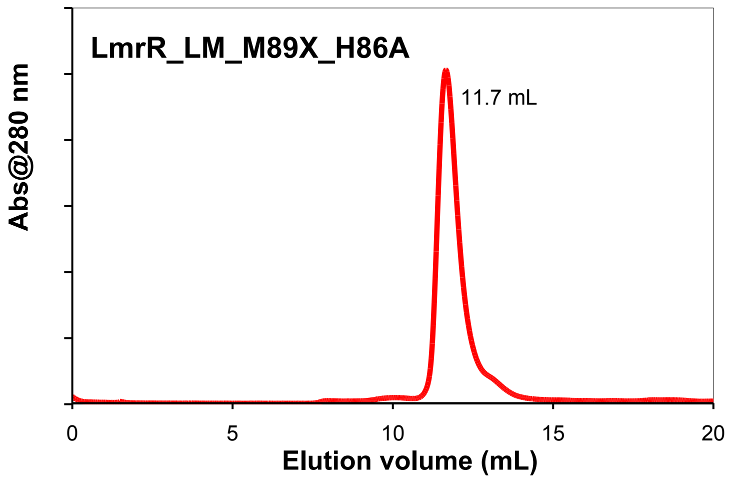
LmrR_LM_M89X_H86A
11.7 mL
Abs@280 nm
0
5
10
15
20
Elution volume (mL)

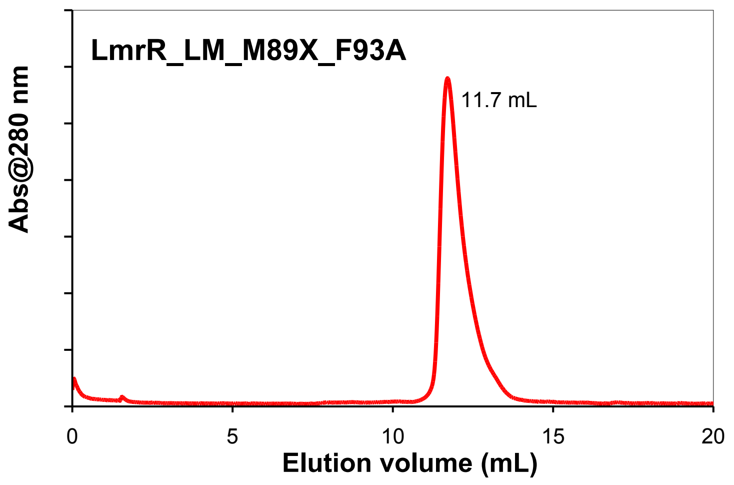
LmrR_LM_M89X_F93A
11.7 mL
Abs@280 nm
0
5
10
15
20
Elution volume (mL)

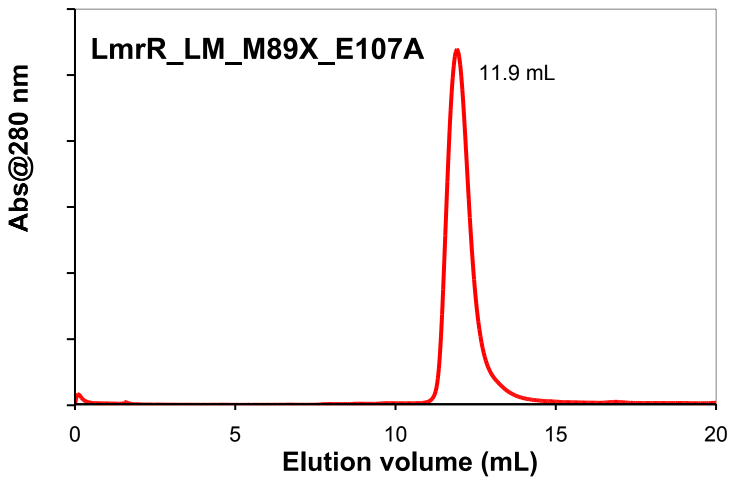
LmrR_LM_M89X_E107A
11.9 mL
Abs@280 nm
0
5
10
15
20
Elution volume (mL)

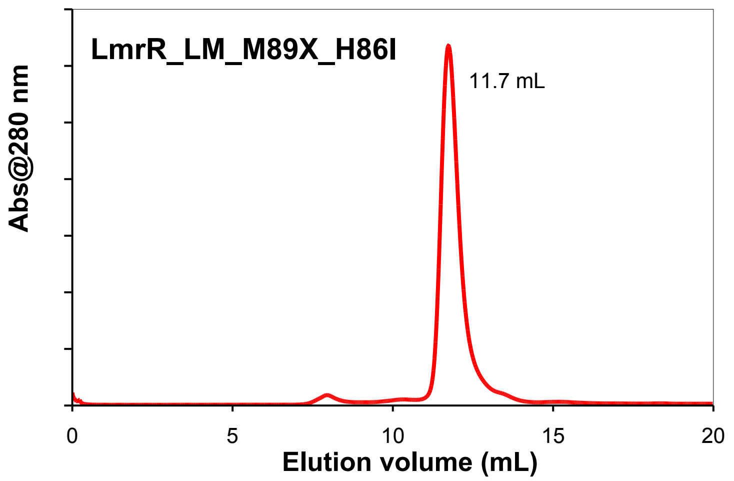
LmrR_LM_M89X_H86I
11.7 mL
Abs@280 nm
0
5
10
15
20
Elution volume (mL)

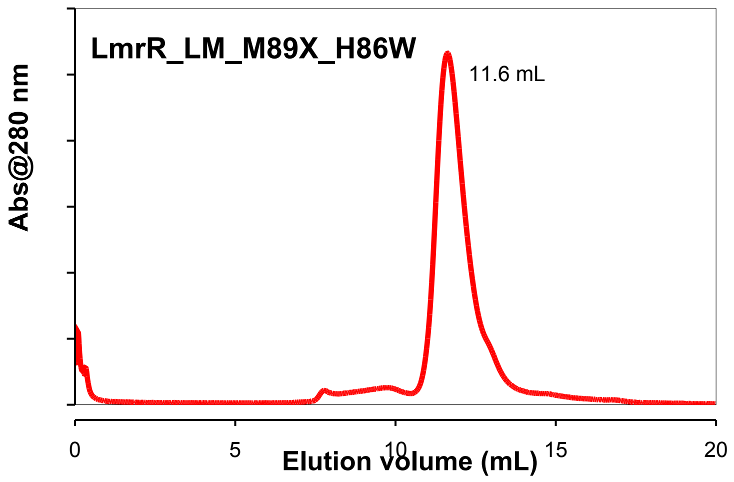
LmrR_LM_M89X_H86W
11.6 mL
Abs@280 nm
0
5
10
15
20
Elution volume (mL)

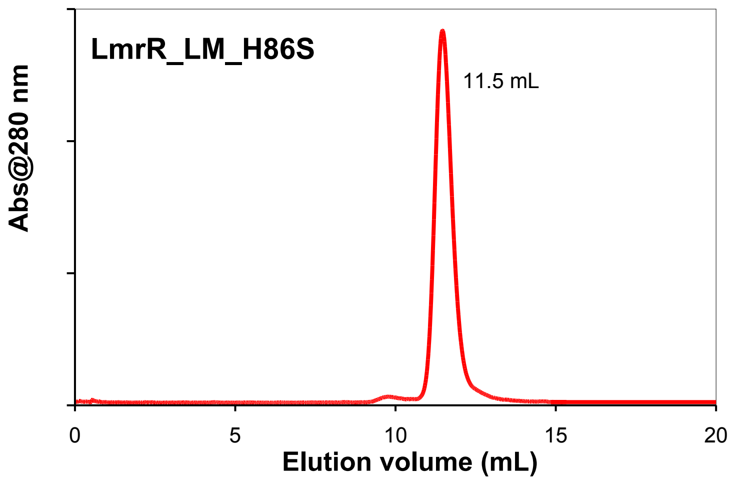

LmrR_LM_H86S
11.5 mL
Abs@280 nm
0
5
10
15
20
Elution volume (mL)

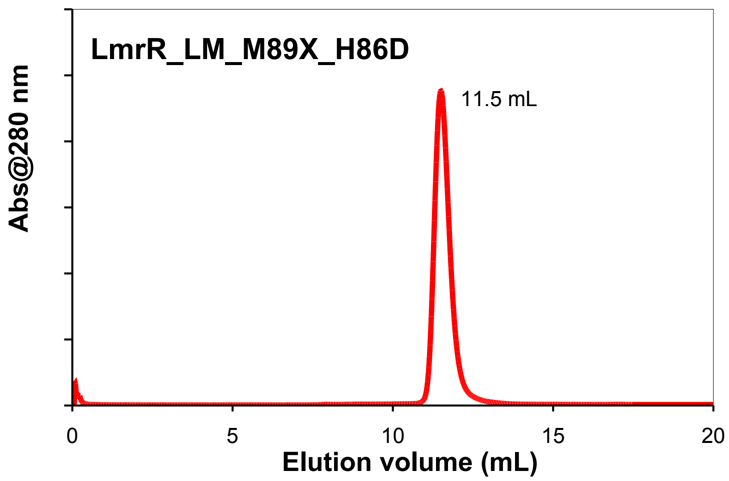

LmrR_LM_M89X_H86D
11.5 mL
Abs@280 nm
0
5
10
15
20
Elution volume (mL)

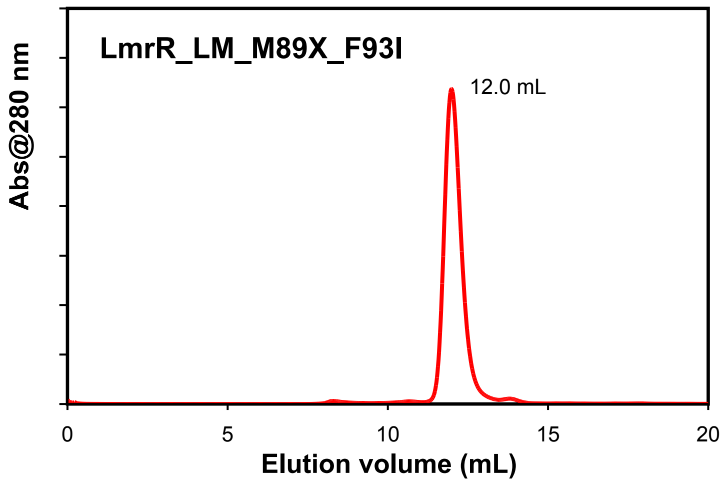

LmrR_LM_M89X_F93I
12.0 mL
Abs@280 nm
0
5
10
15
20
Elution volume (mL)

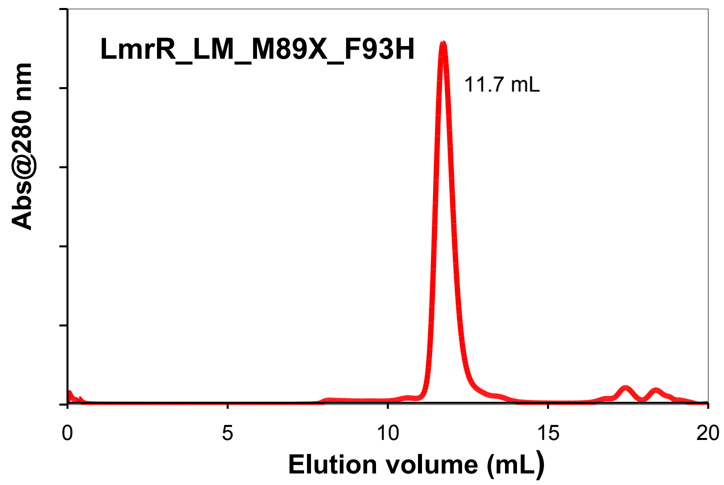
LmrR_LM_M89X_F93H
11.7 mL
Abs@280 nm
0
5
10
15
20
Elution volume (mL)

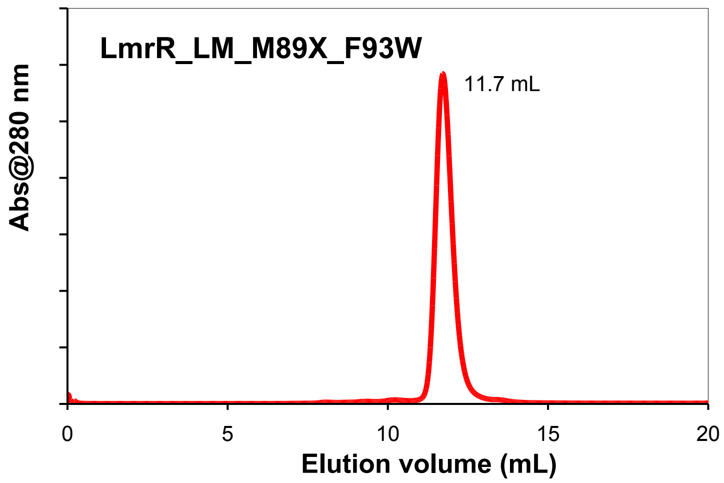
LmrR_LM_M89X_F93W
11.7 mL
Abs@280 nm
0
5
10
15
20
Elution volume (mL)

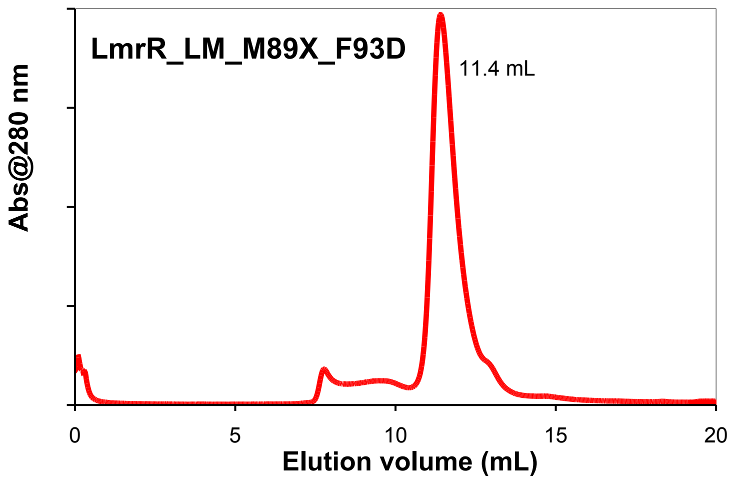
LmrR_LM_M89X_F93D
11.4 mL
Abs@280 nm
0
5
10
15
20
Elution volume (mL)

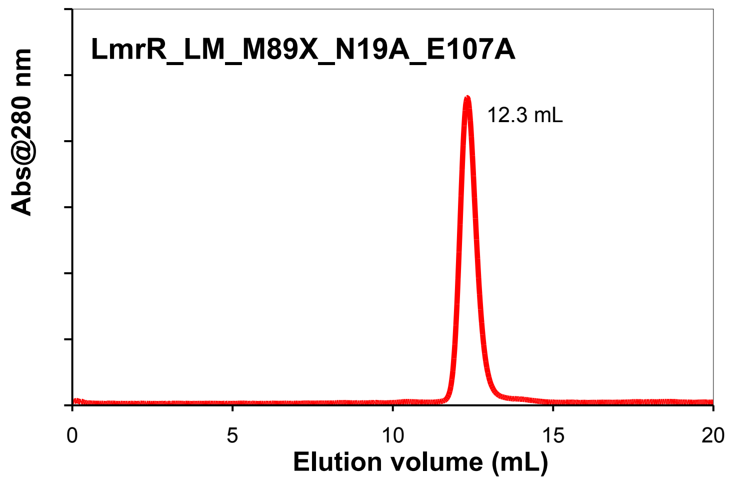
LmrR_LM_M89X_N19A_E107A
12.3 mL
Abs@280 nm
0
5
10
15
20
Elution volume (mL)

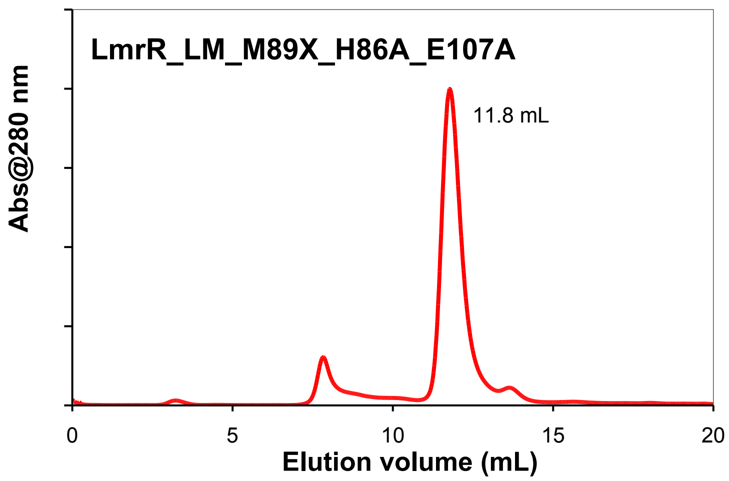
LmrR_LM_M89X_H86A_E107A
11.8 mL
Abs@280 nm
0
5
10
15
20
Elution volume (mL)

7. ESI-mass spectra

Figure S3. Electrospray ionization (ESI) mass spectra LmrR_X mutants.

LmrR_LM_N19X calculated mass (-Met) 15082.79

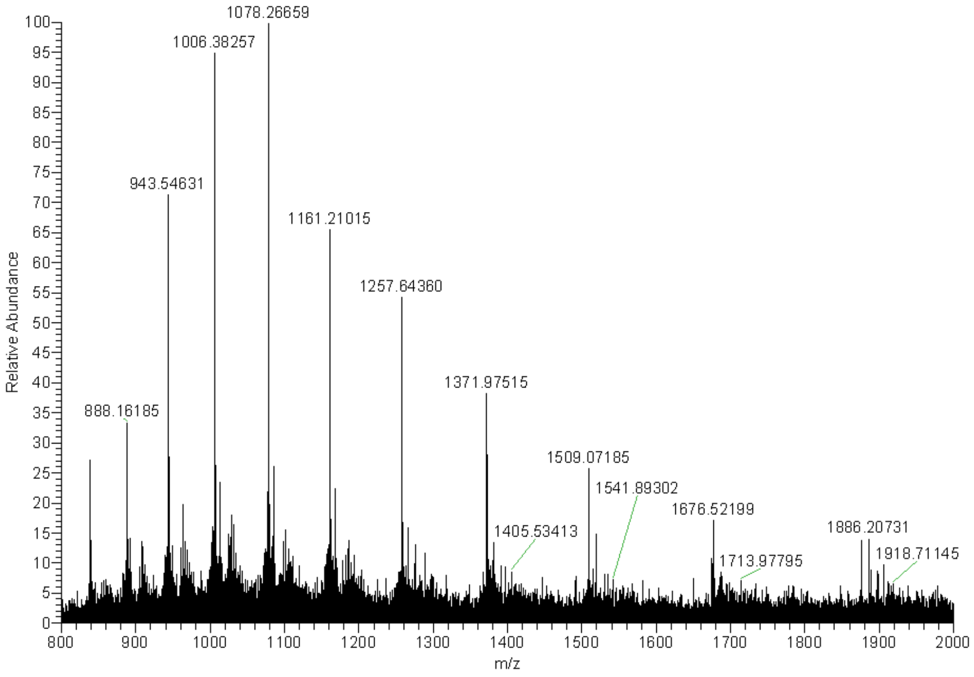

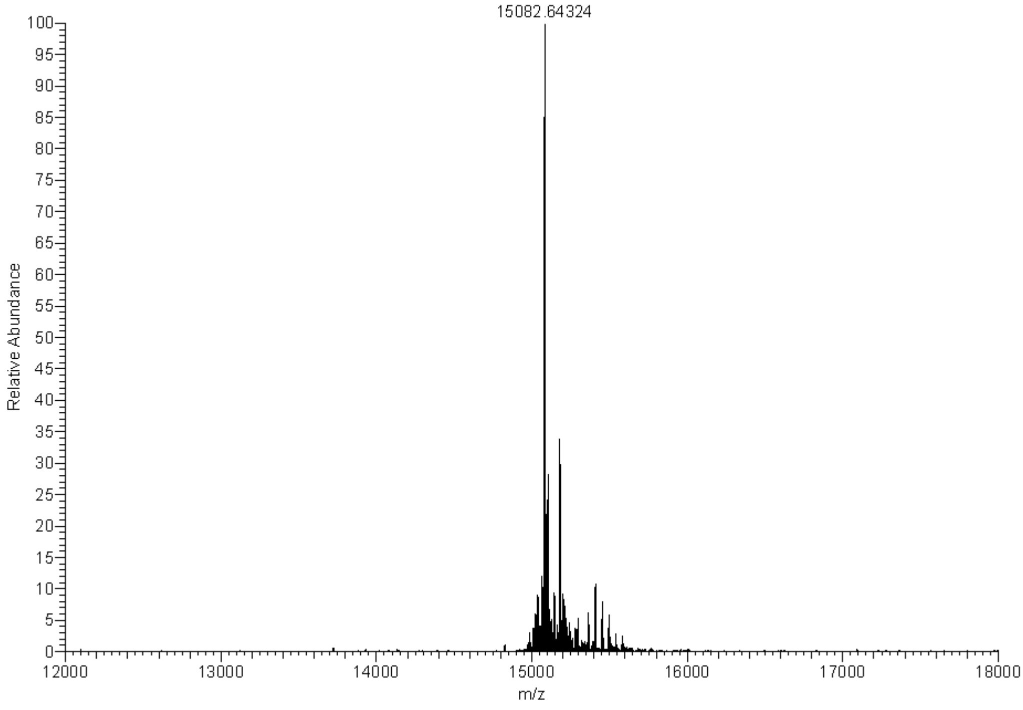

LmrR_LM_M89X calculated mass (-Met) 15065.70

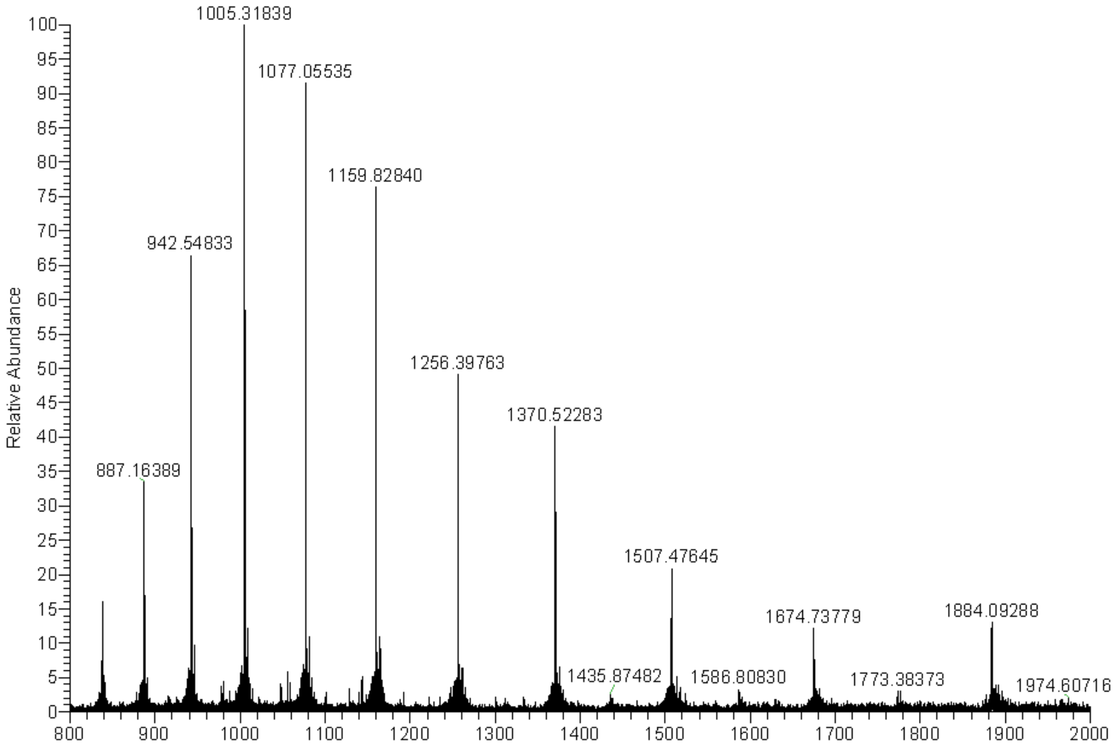

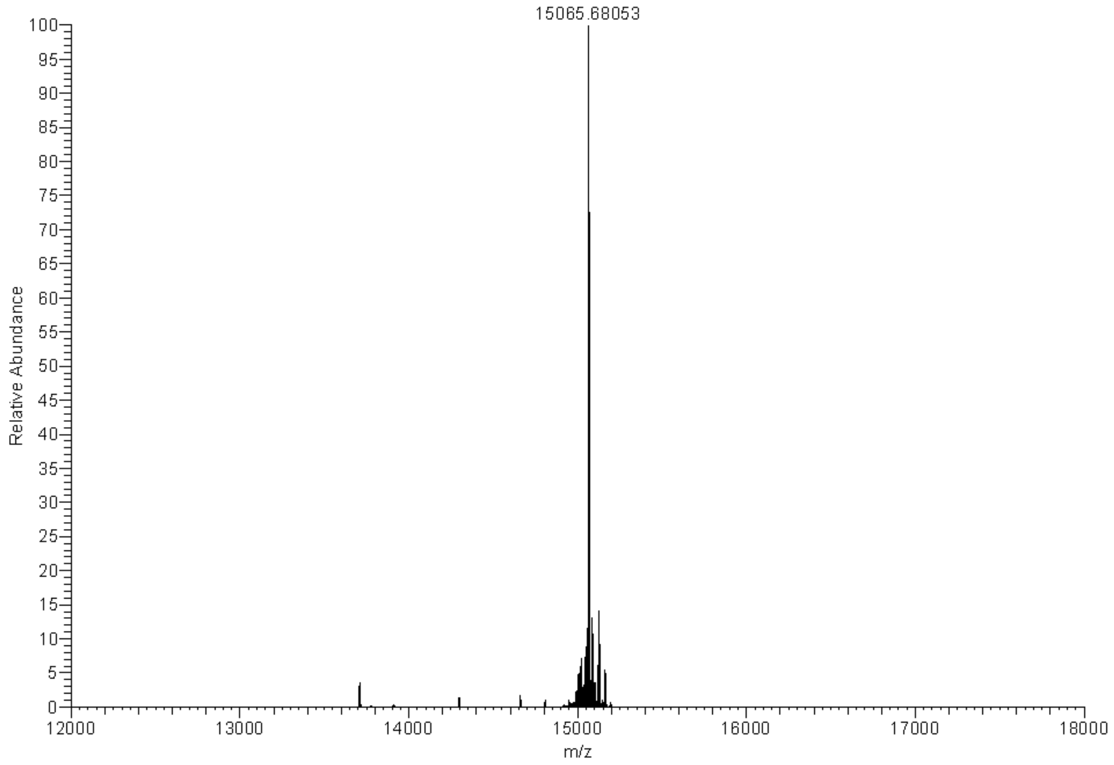

LmrR_LM_F93X calculated mass (-Met) 15049.72

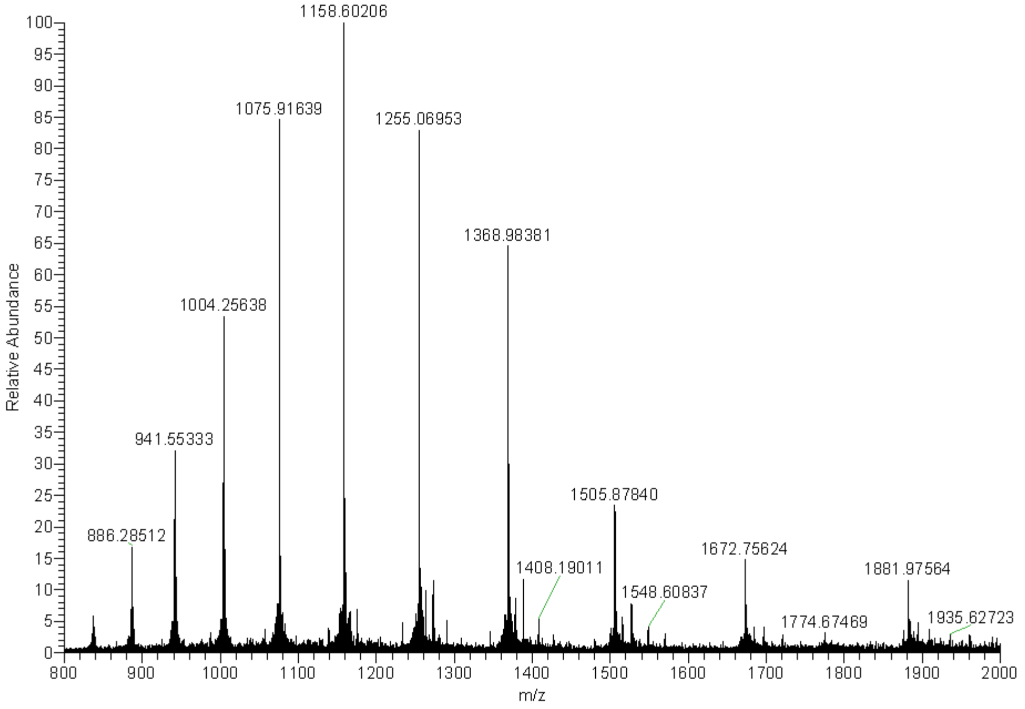

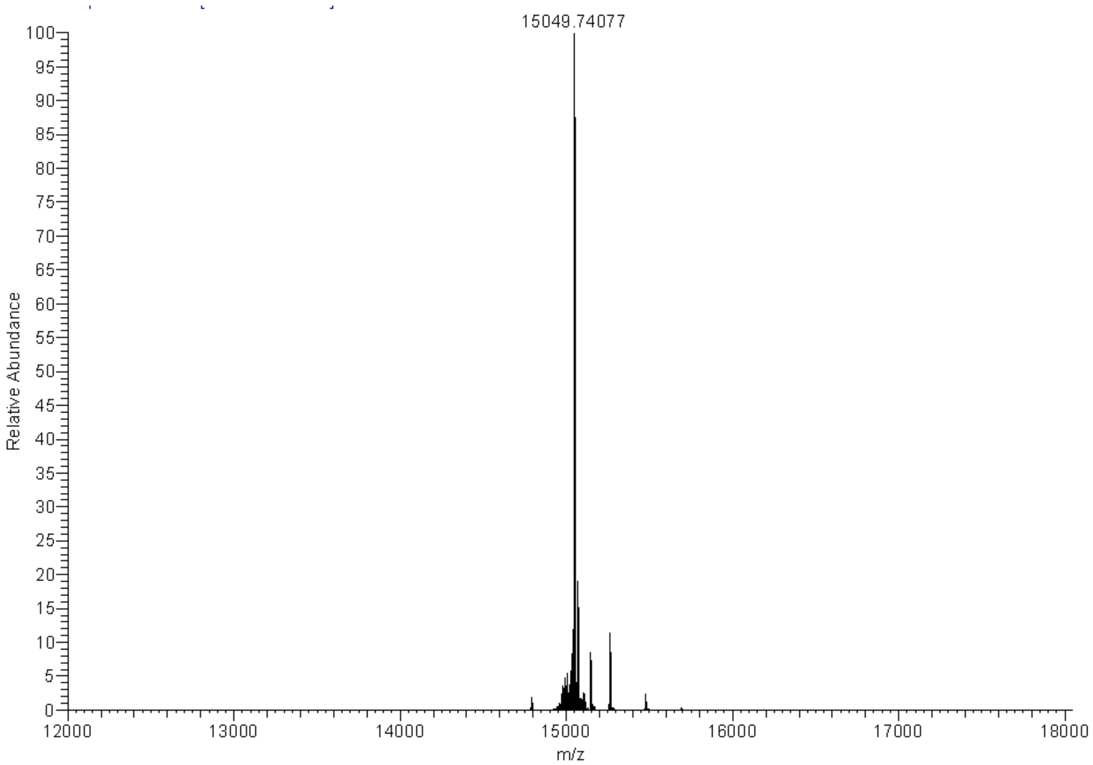

LmrR_LM_M89X_N19A calculated mass (-Met) 15022.68

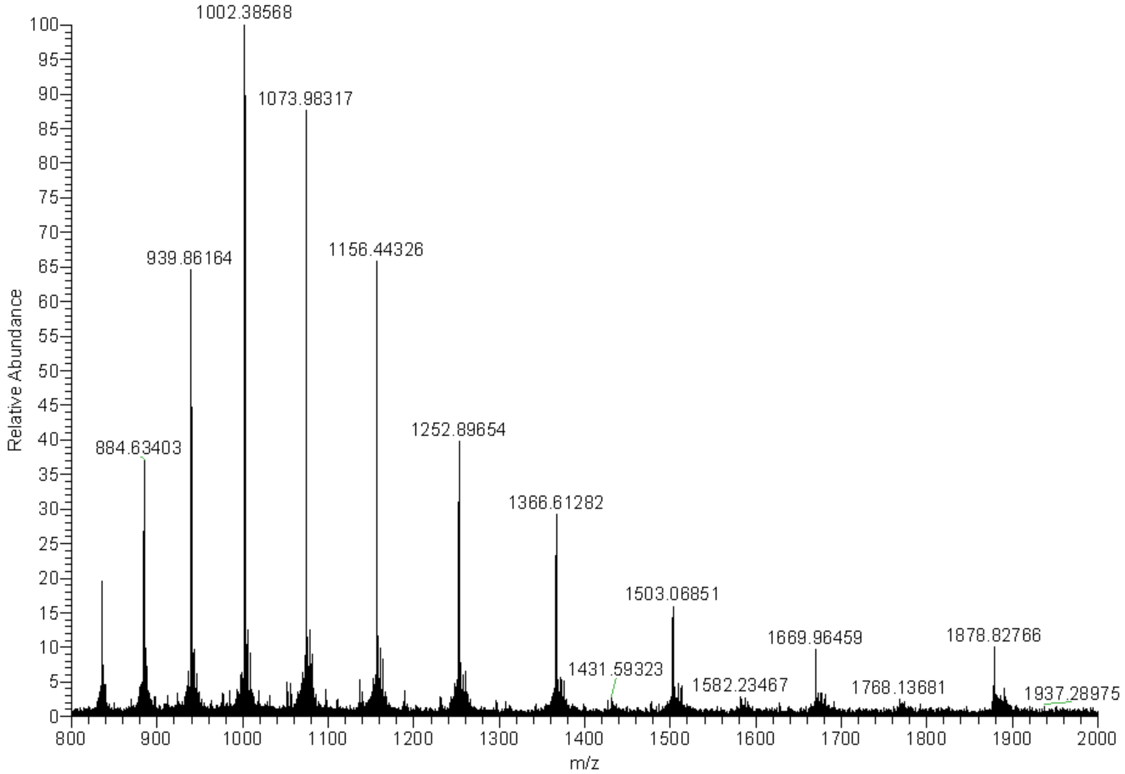

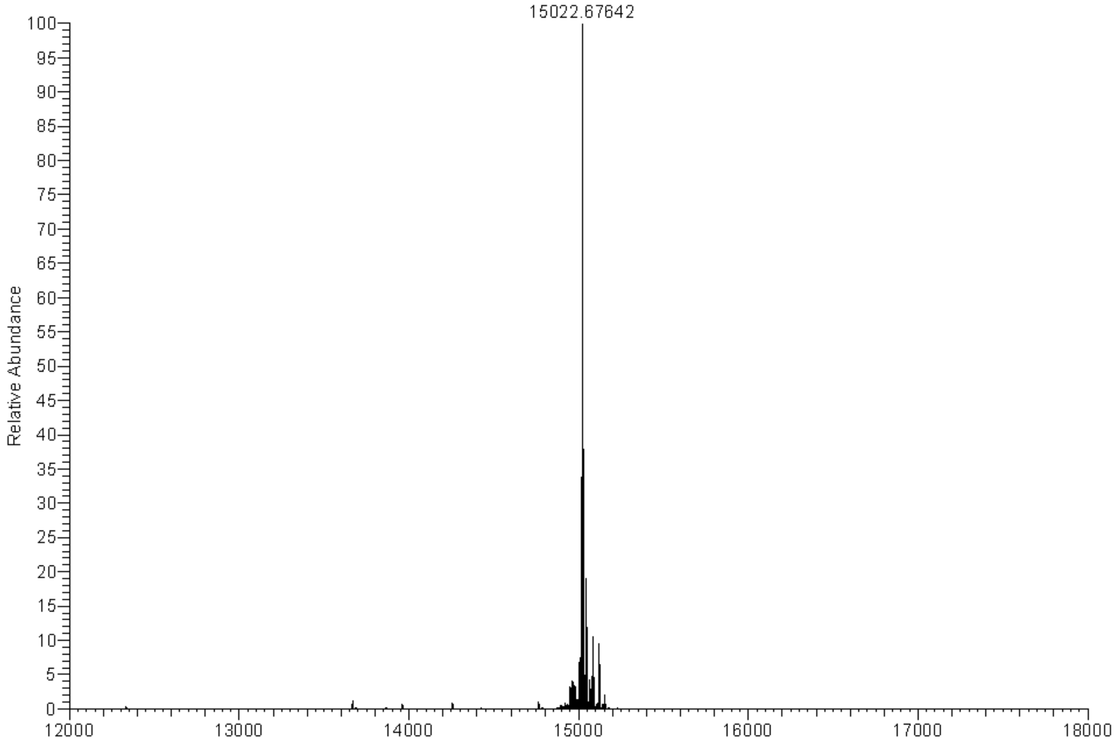

LmrR_LM_M89X_K22A calculated mass (-Met) 15008.61

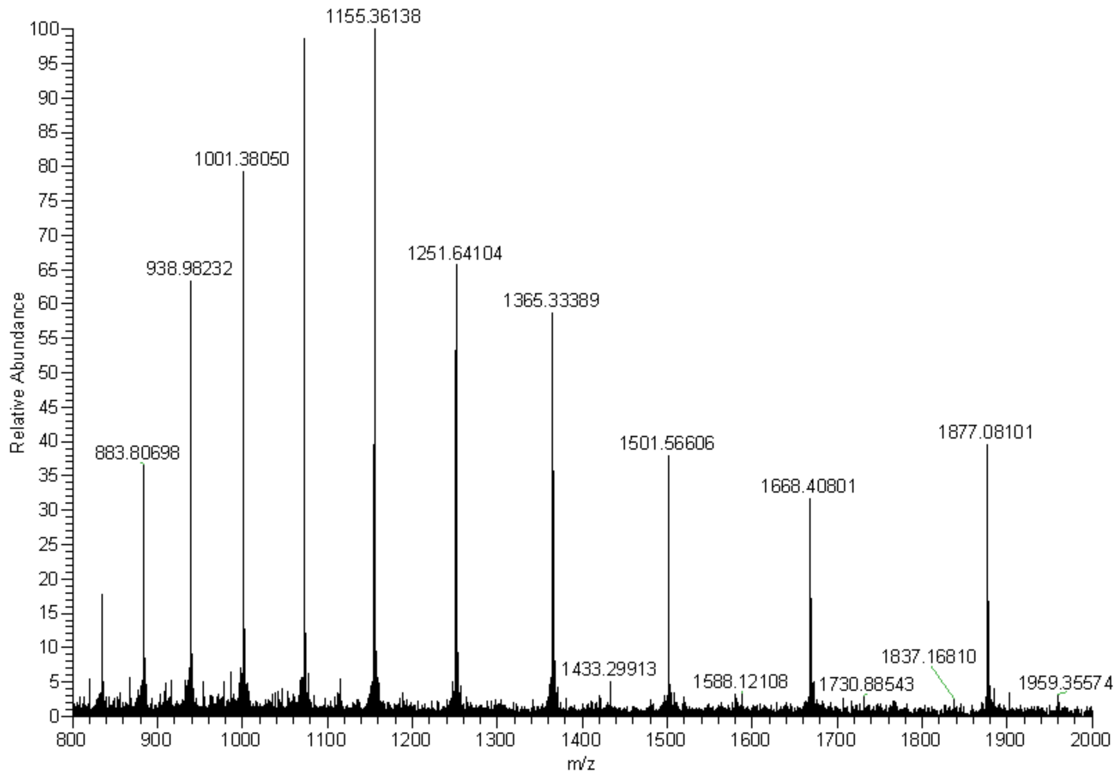

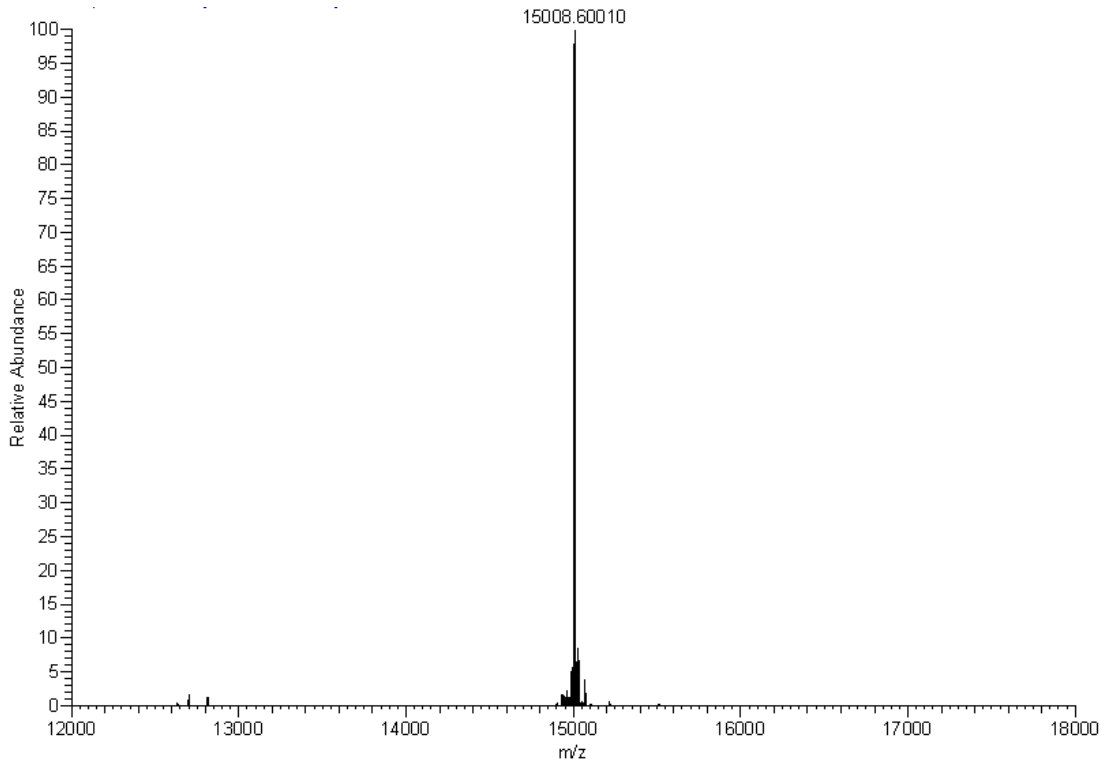

LmrR_LM_M89X_H86A calculated mass (-Met) 14999.64

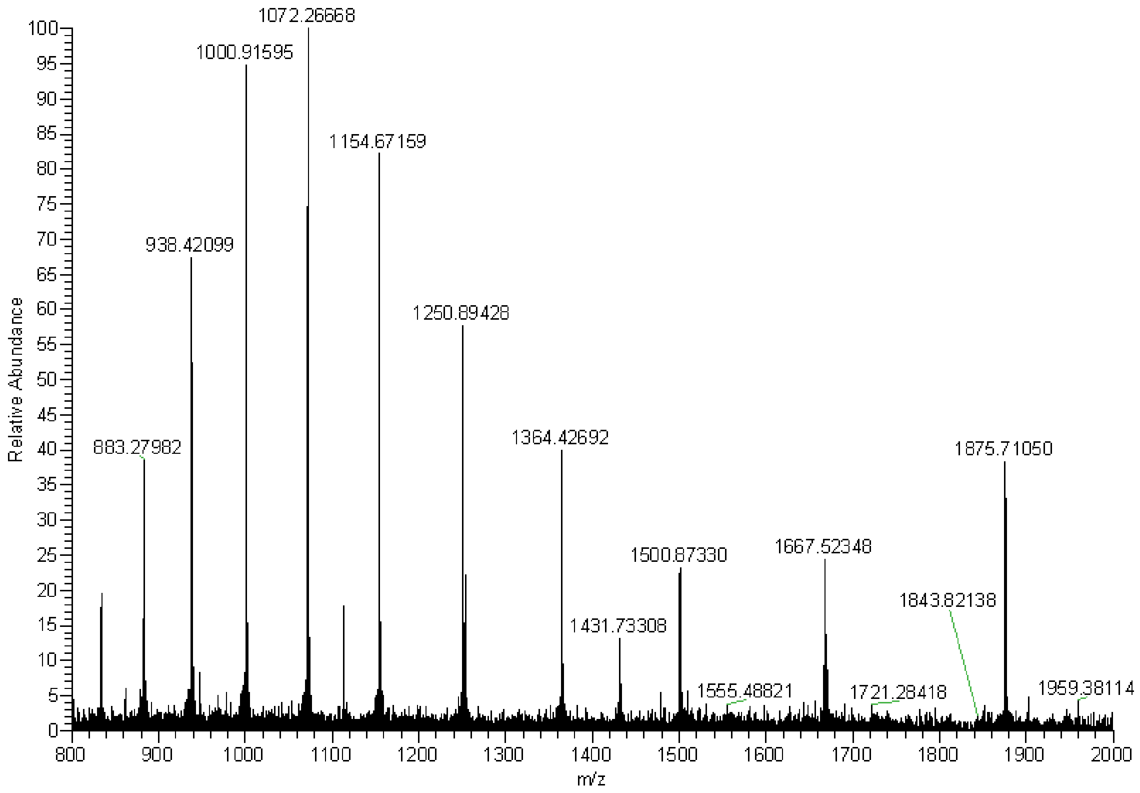

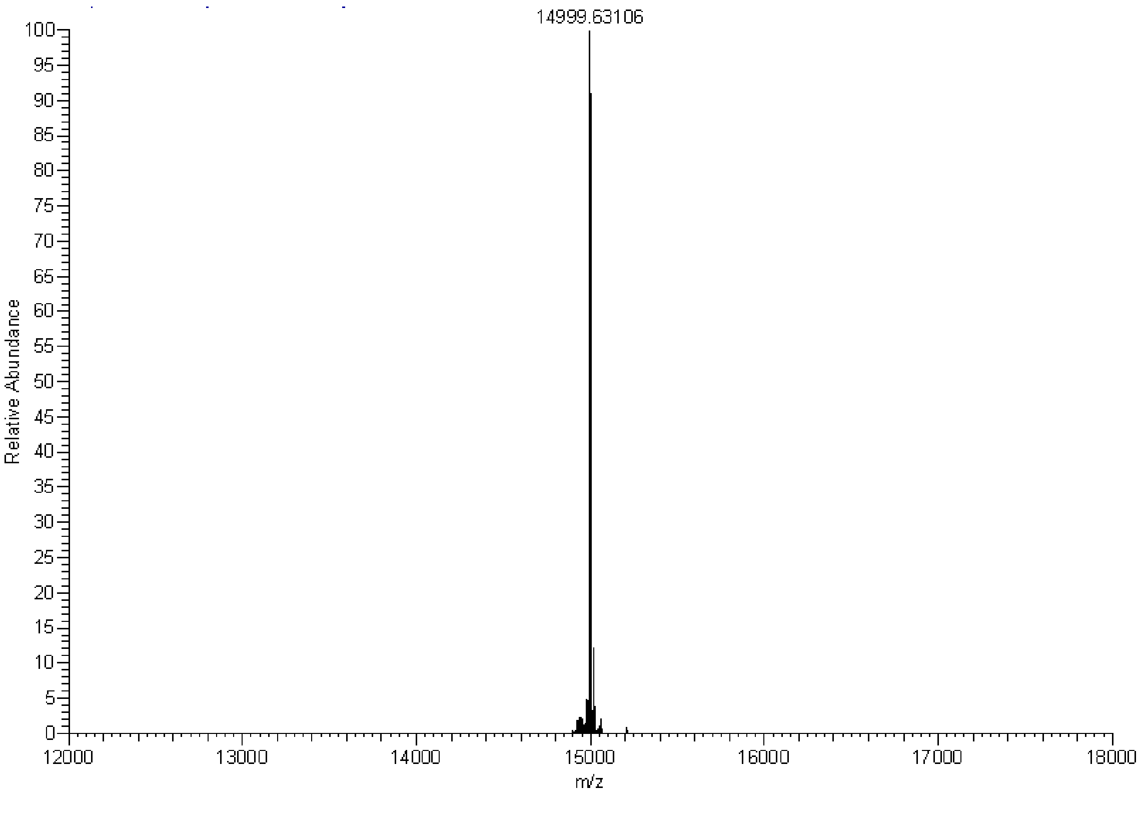

LmrR_LM_M89X_F93A calculated mass (-Met) 14989.60

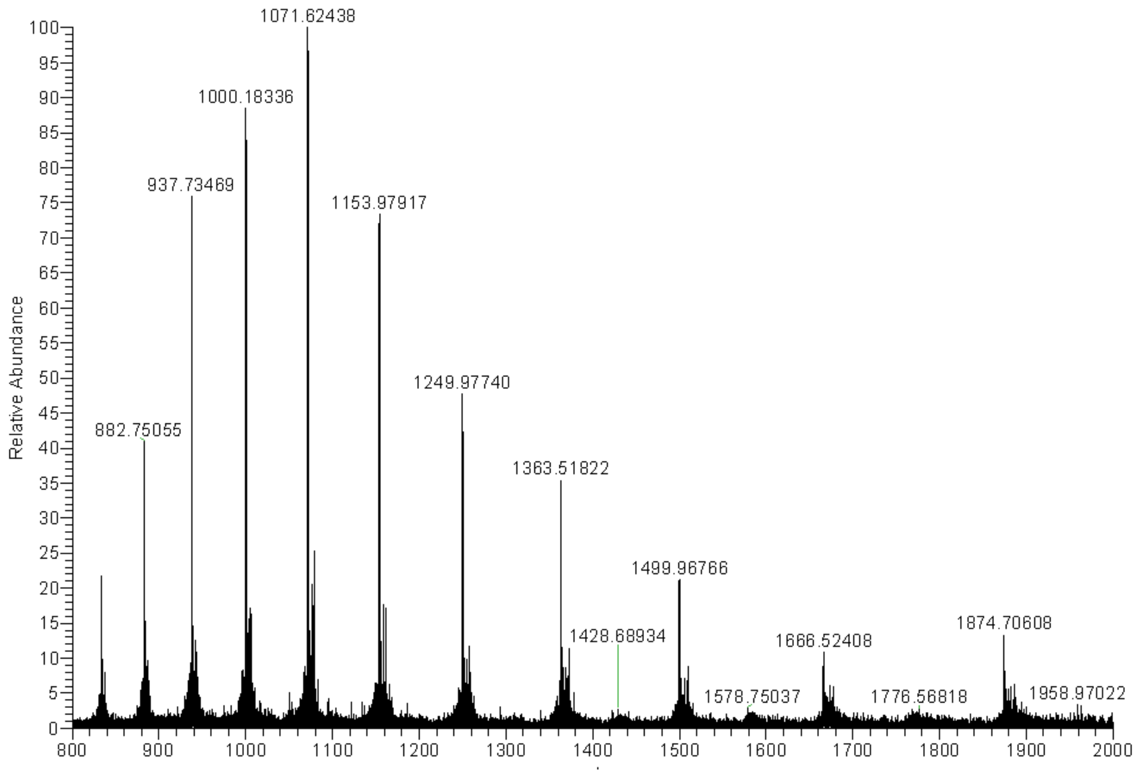

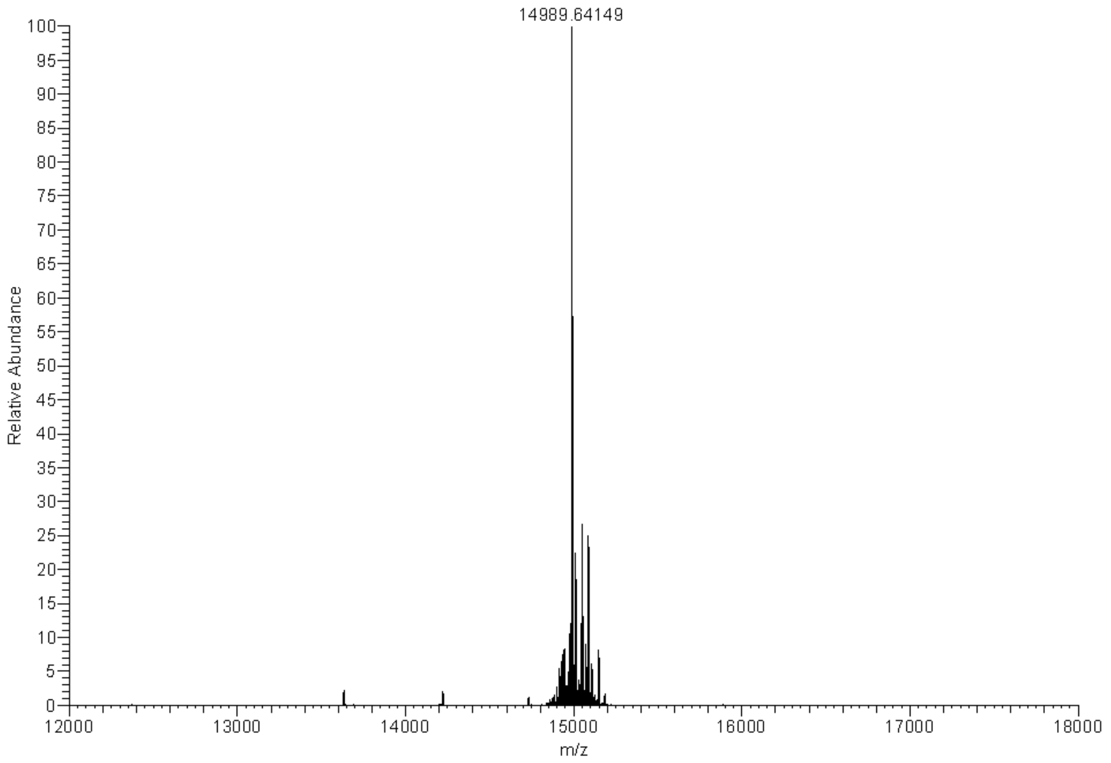

LmrR_LM_M89X_E107A calculated mass (-Met) 15007.57

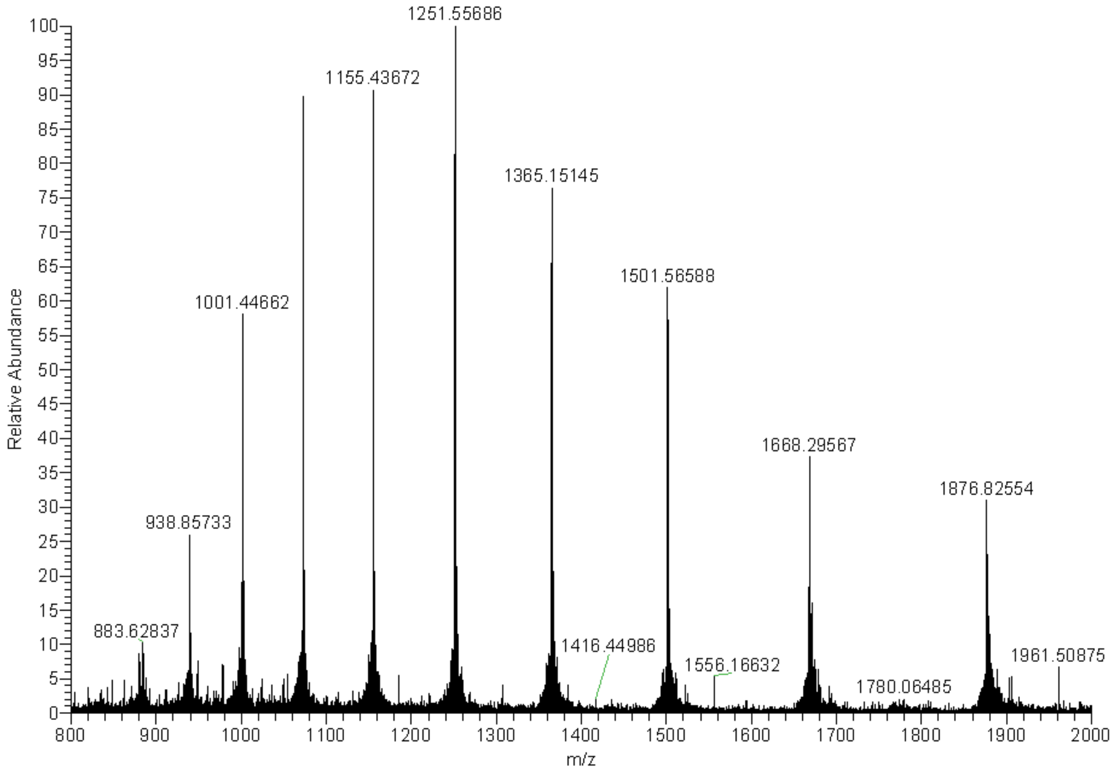

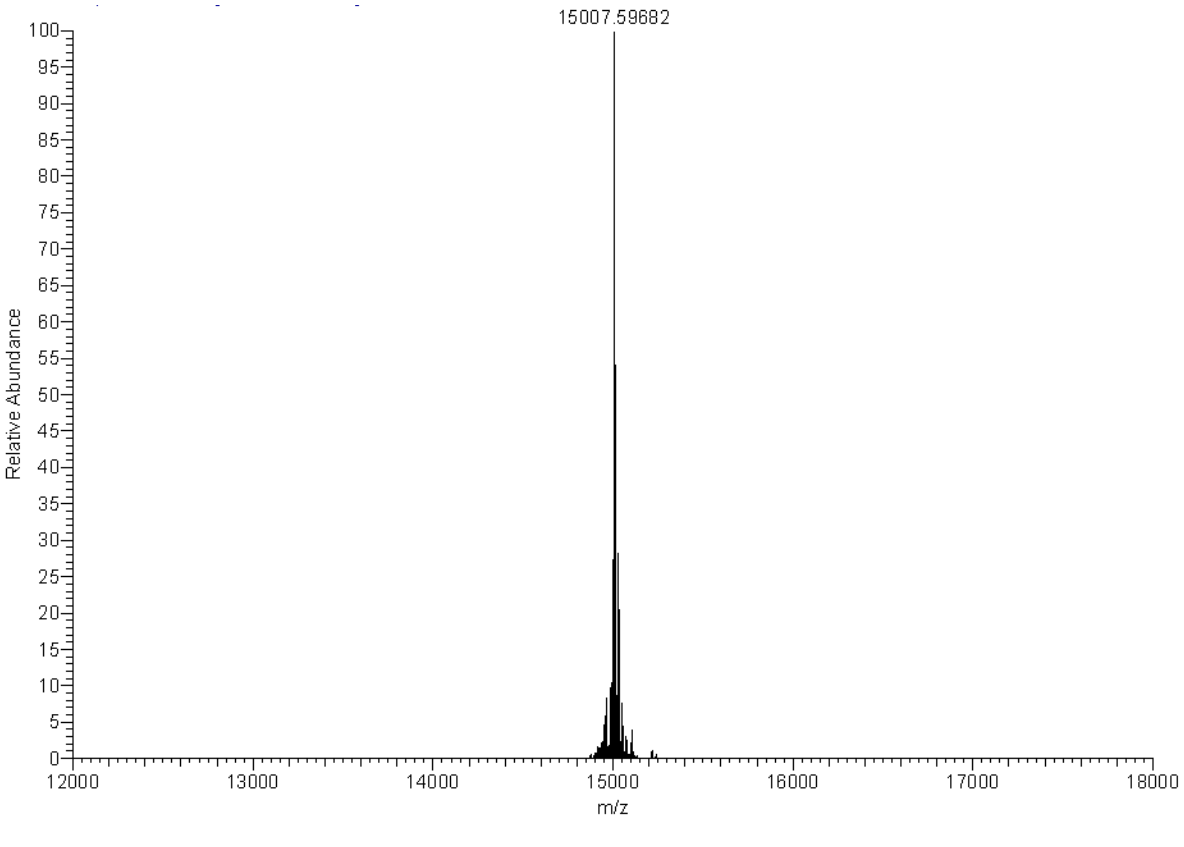

LmrR_LM_ M89X_H86I calculated mass (-Met) 15041.72

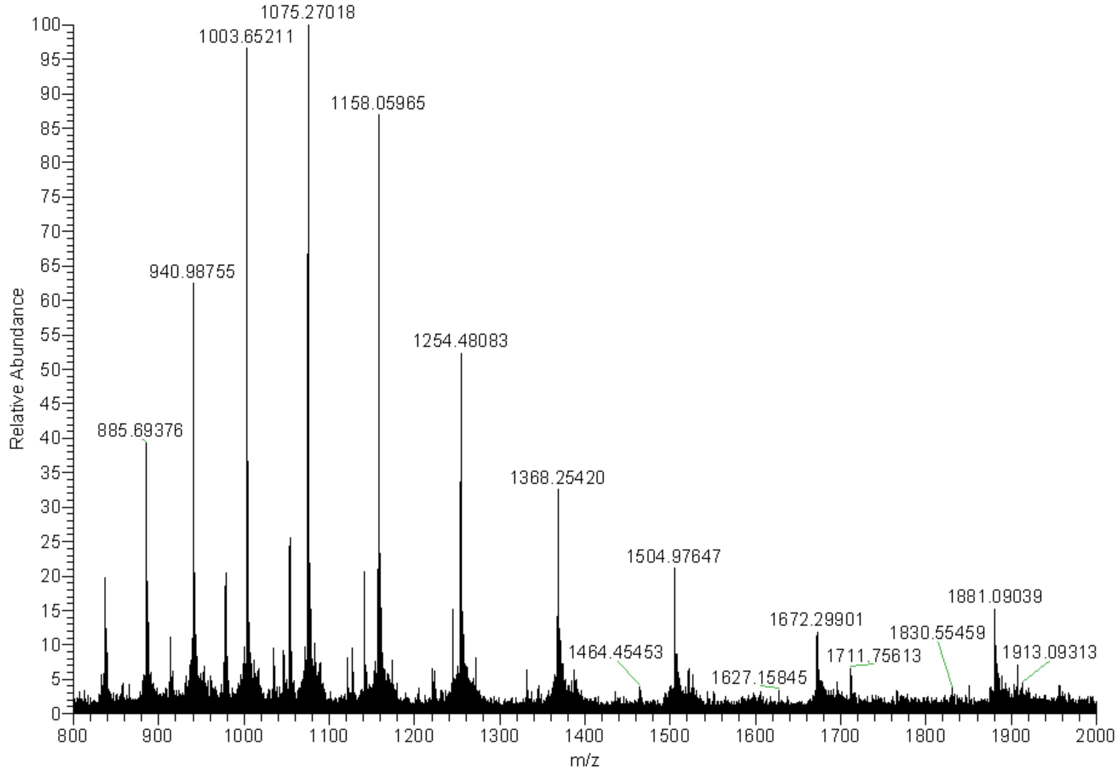

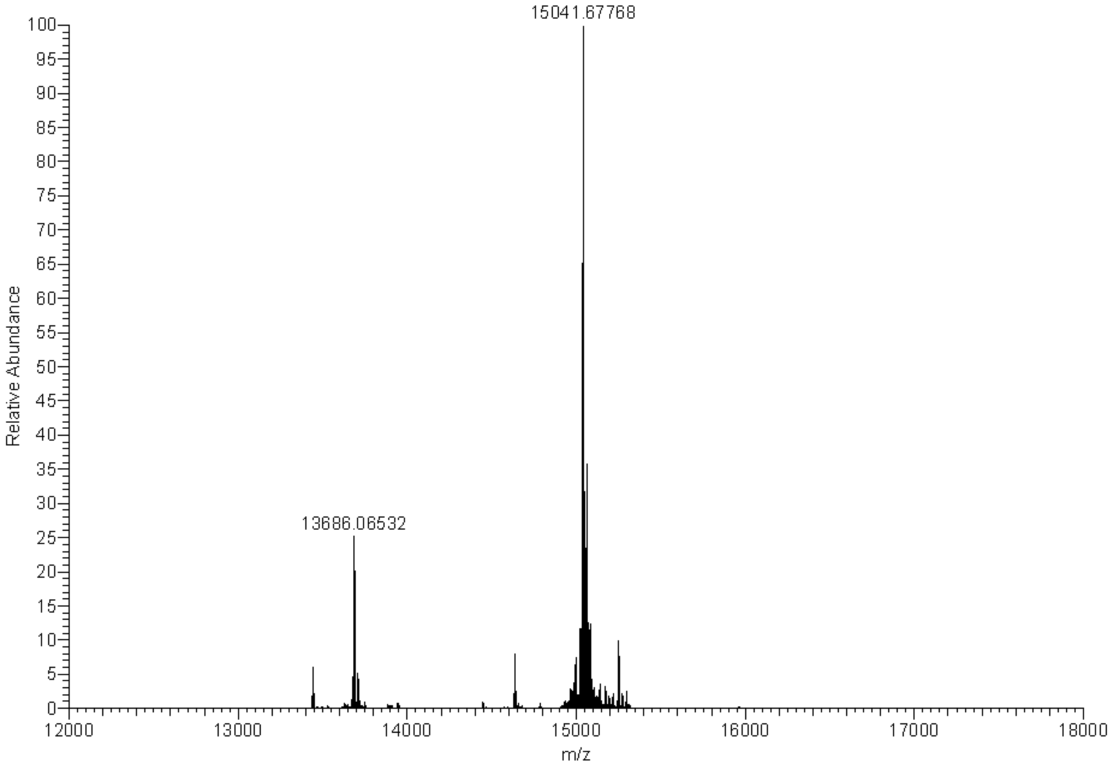

13686.06 – protein without terminal 13 amino acids (GGSGG + Streptag)

LmrR_LM_M89X_H86W calculated mass (-Met) 15114.77

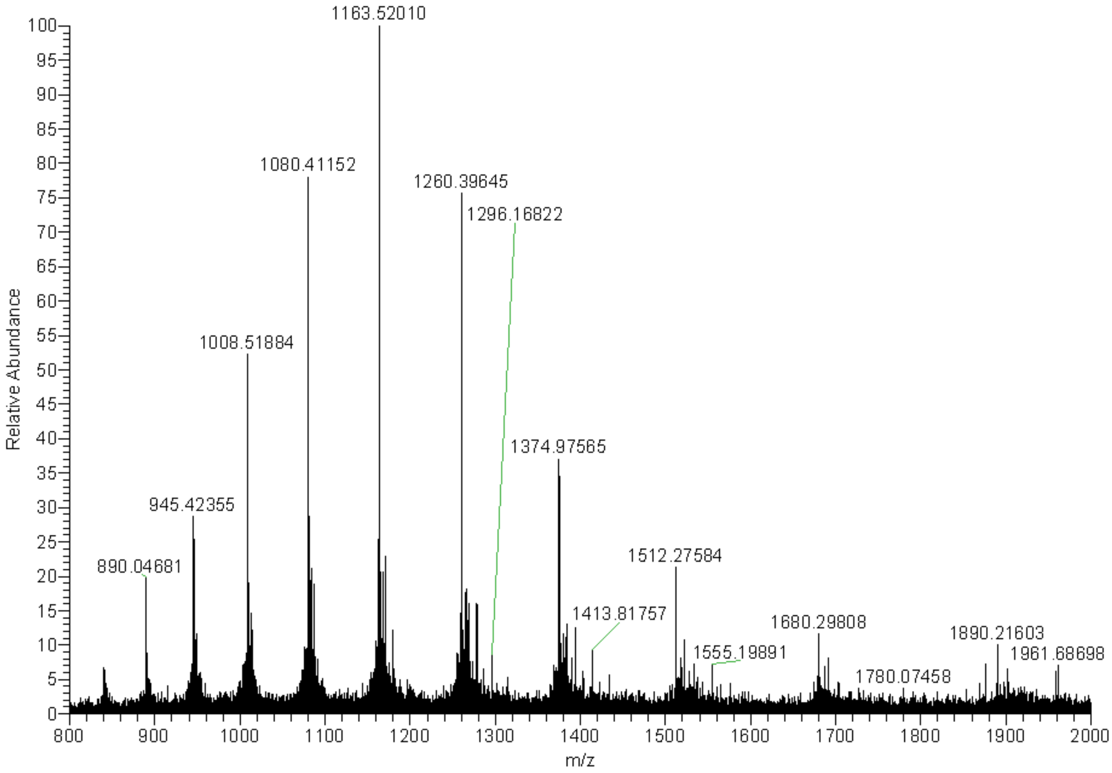

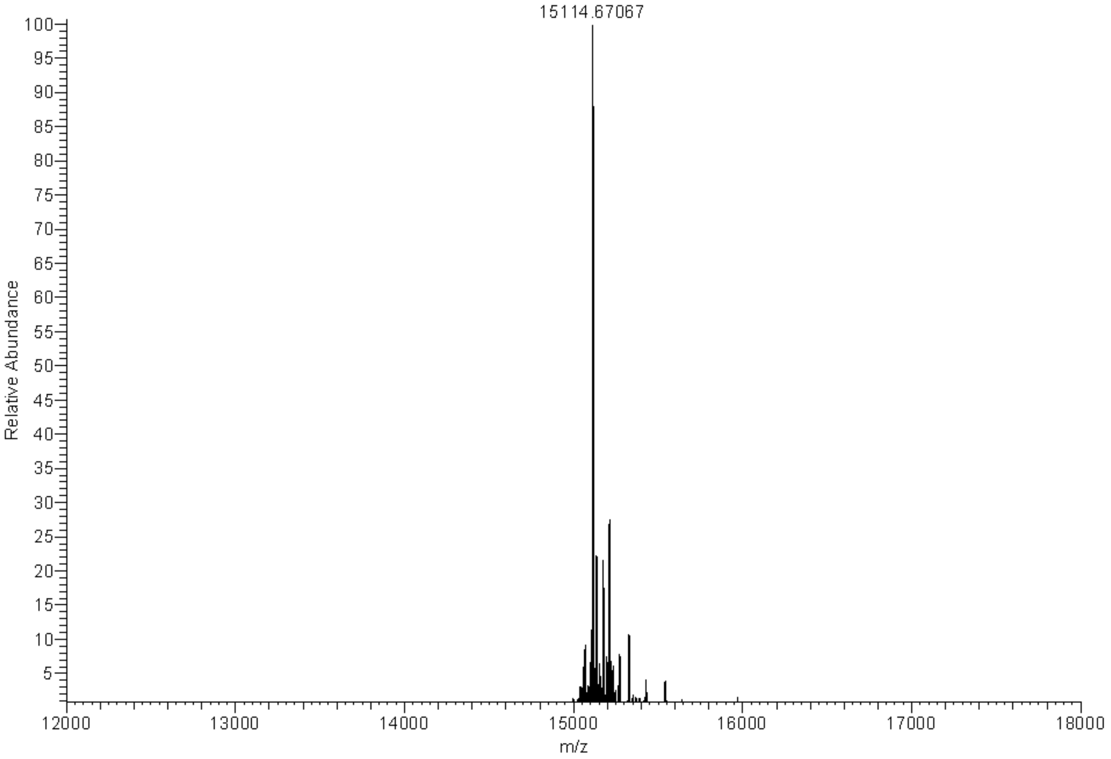

LmrR_LM_M89X_H86S calculated mass (-Met) 15015.64

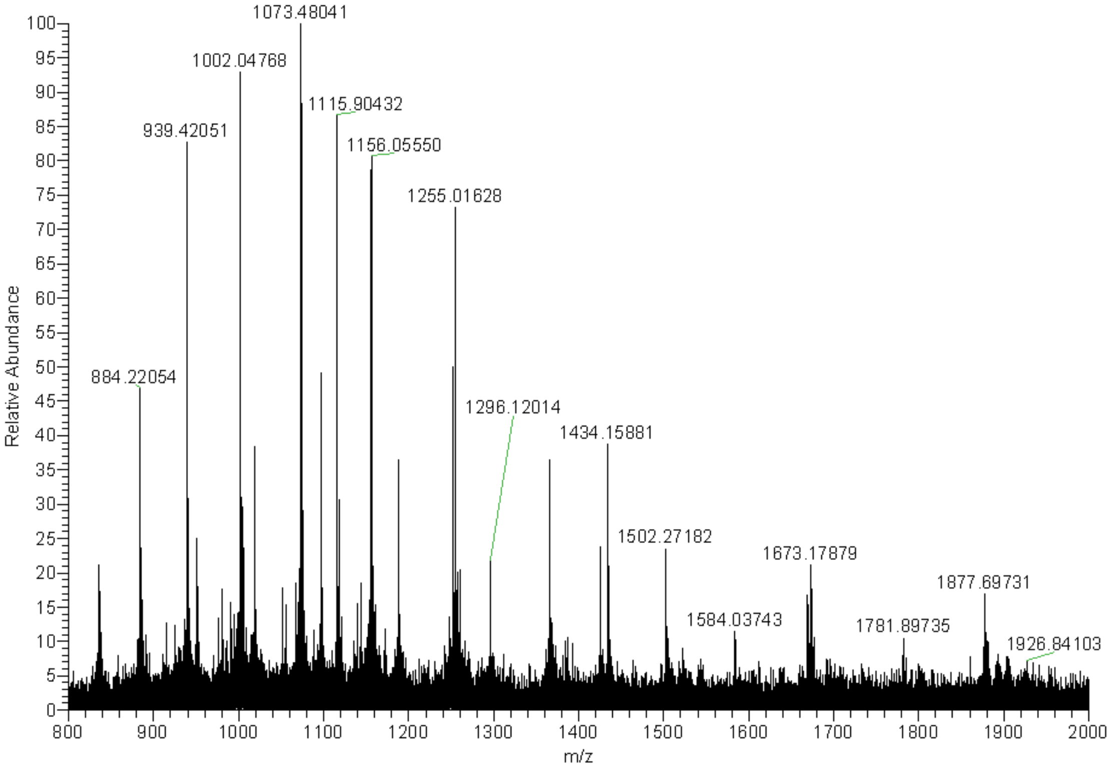

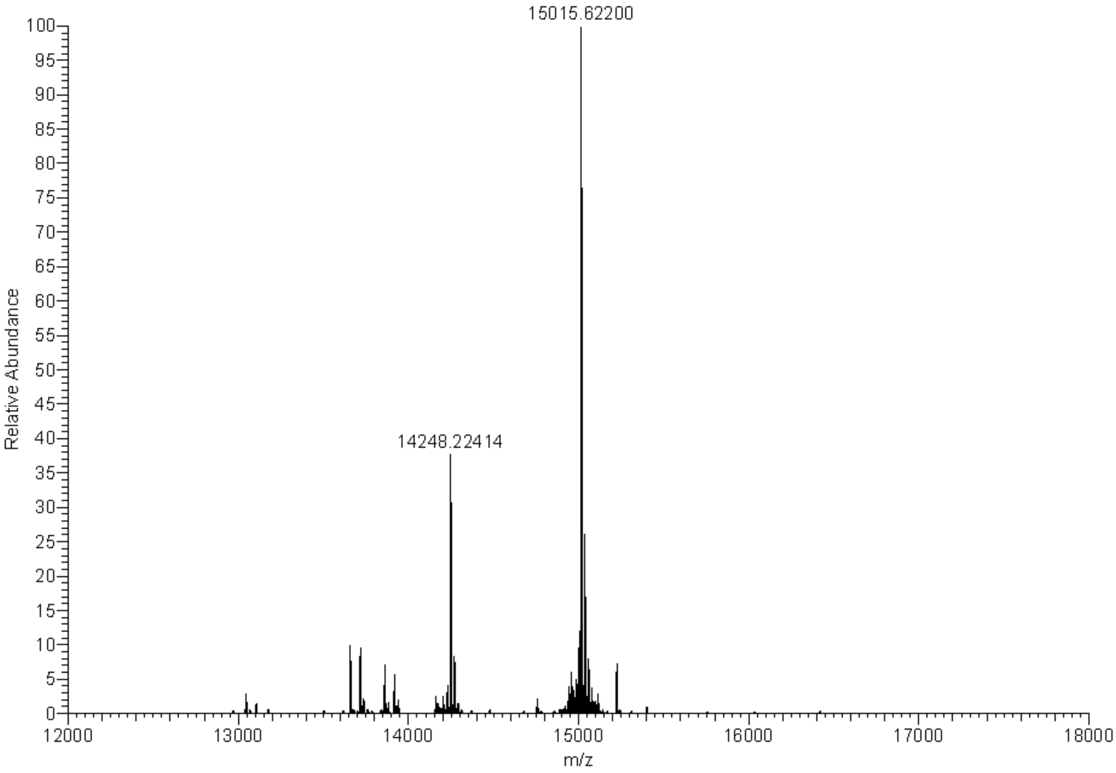

14248.22 – protein without terminal last 6 amino acids (6 out of 8 amino acids of Strep-tag)

LmrR_LM_M89X_H86D calculated mass (-Met) 15043.65

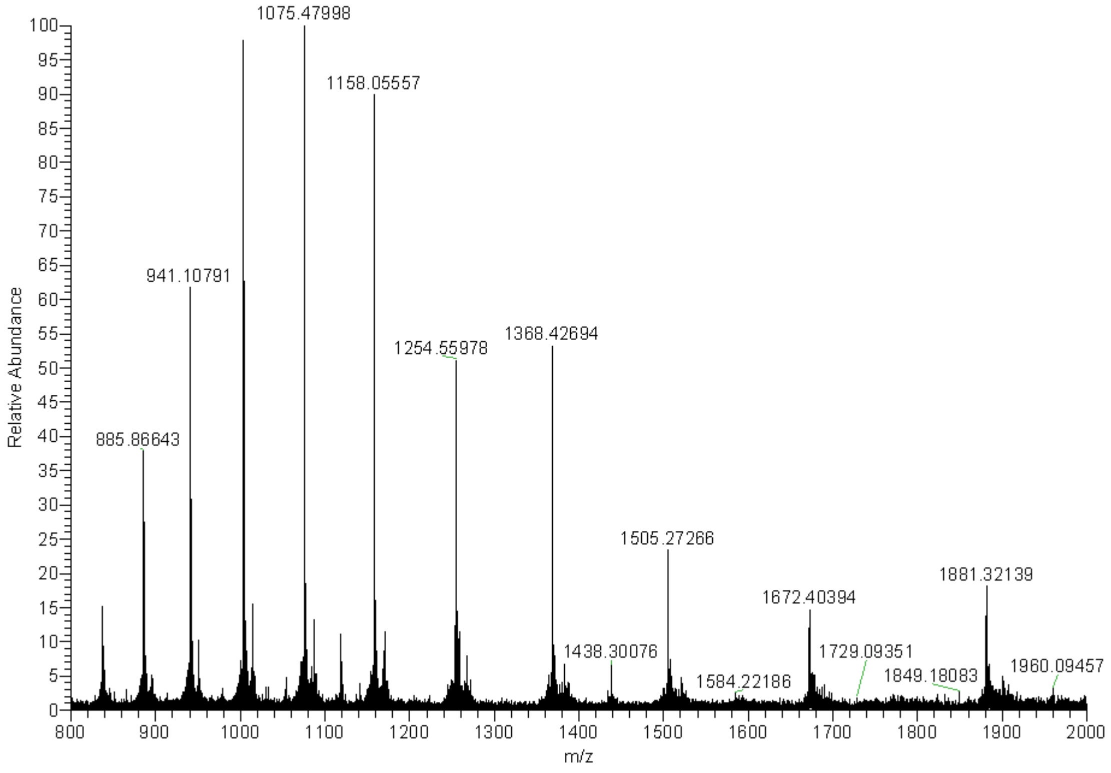

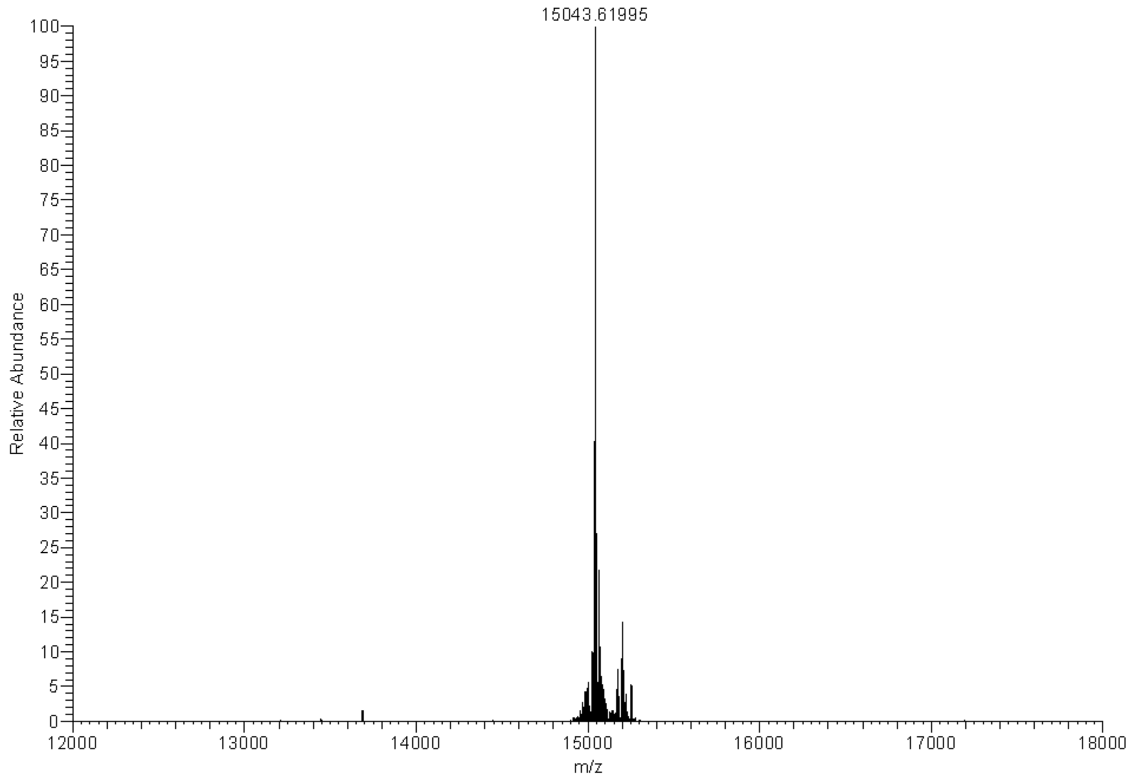

LmrR_LM_ M89X_F93I calculated mass (-Met) 15031.67

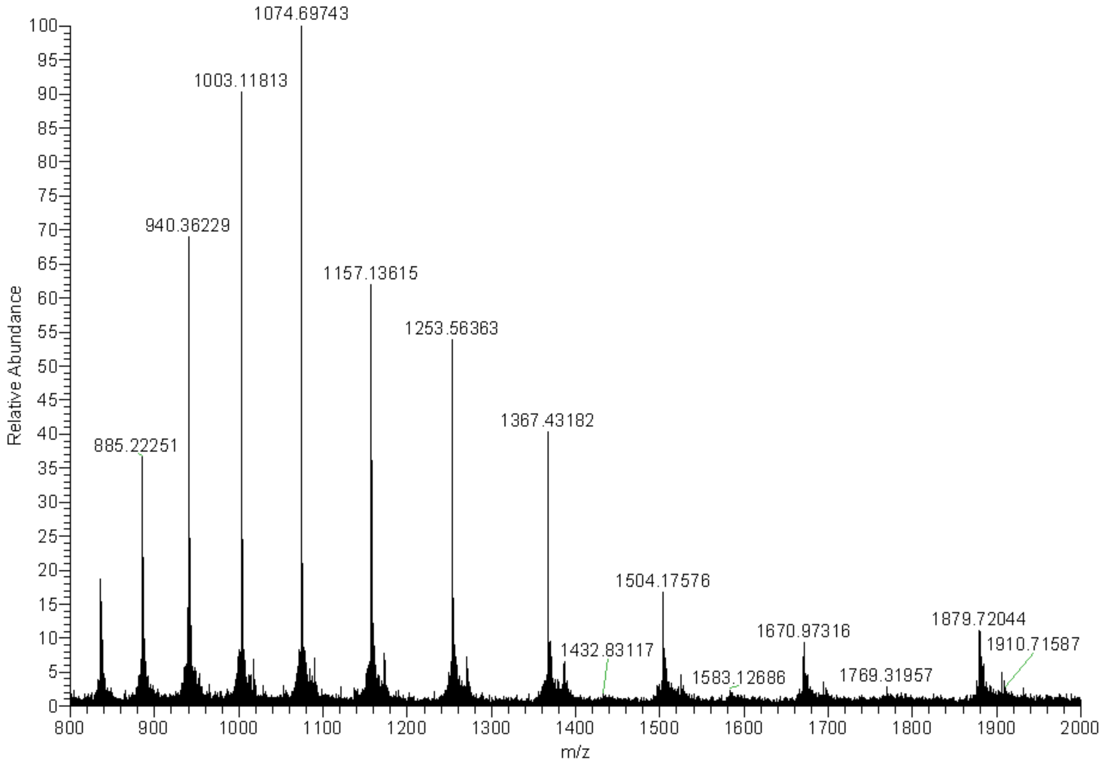

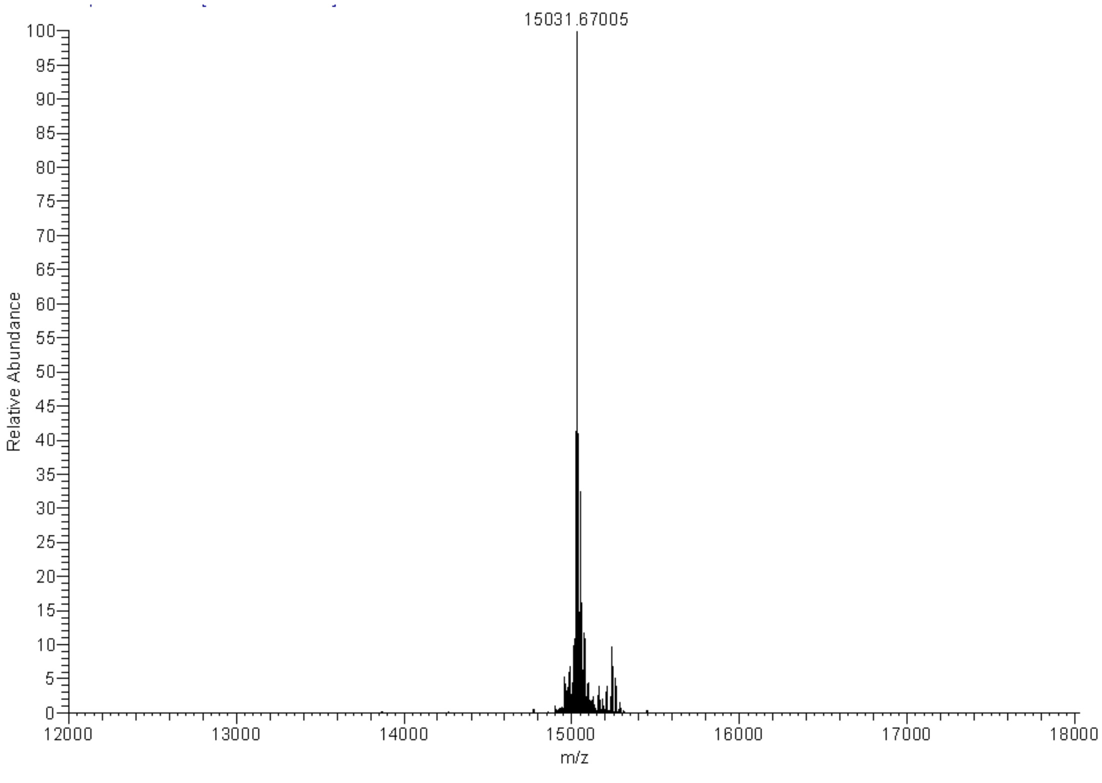

LmrR_LM_M89X_F93H calculated mass (-Met) 15055.67

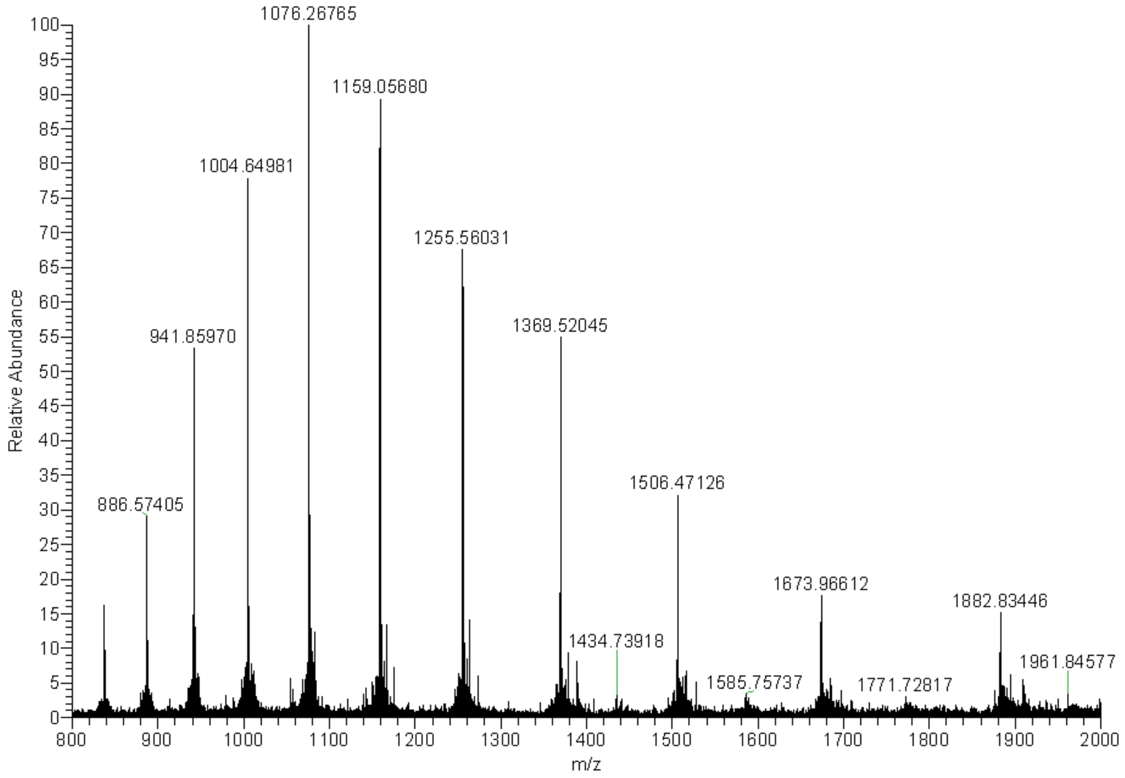

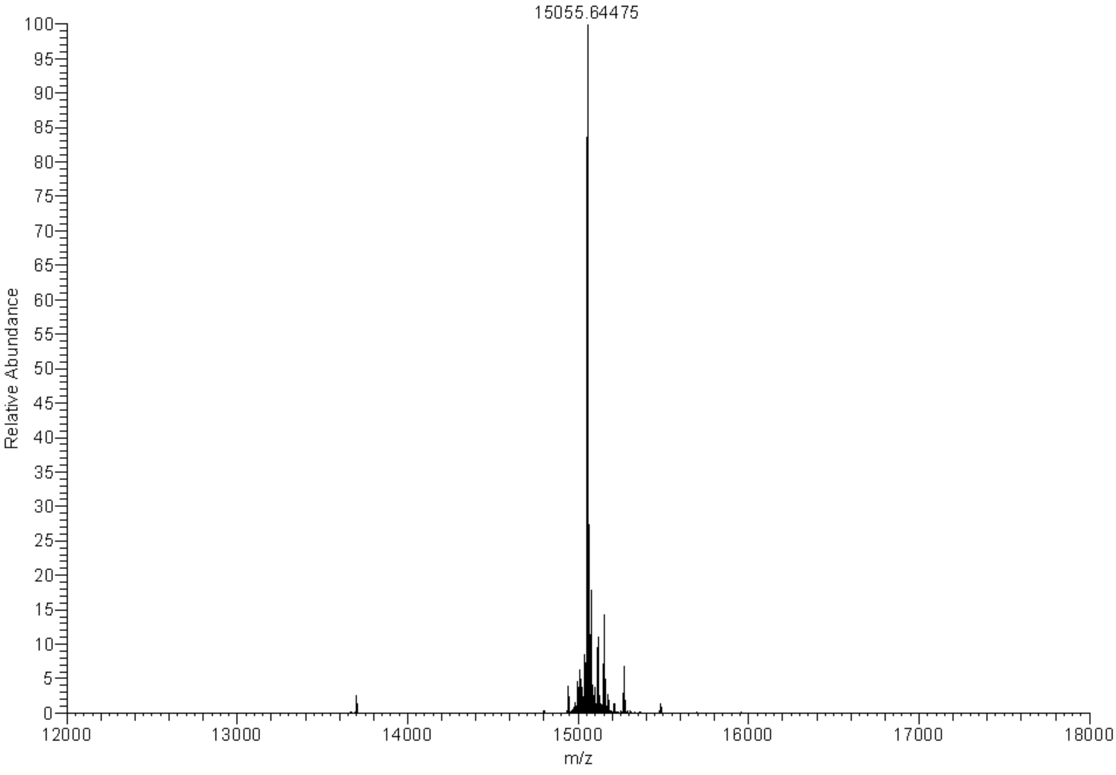

LmrR_LM_M89X_F93W calculated mass (-Met) 15104.74

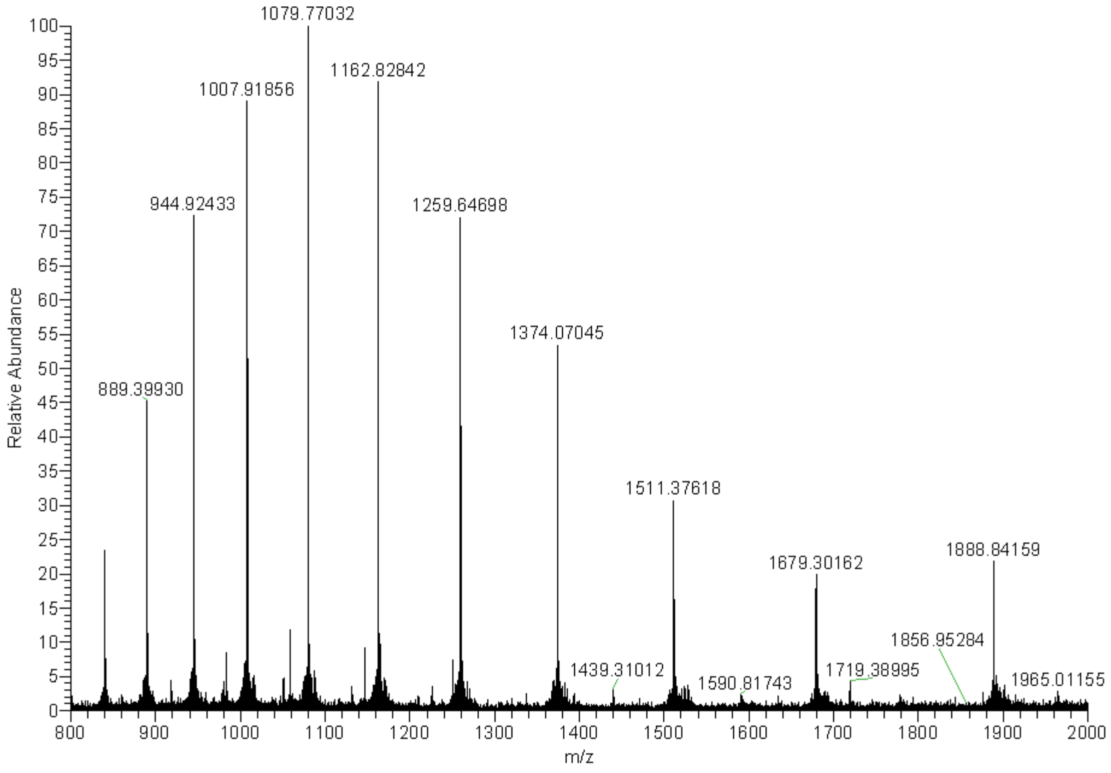

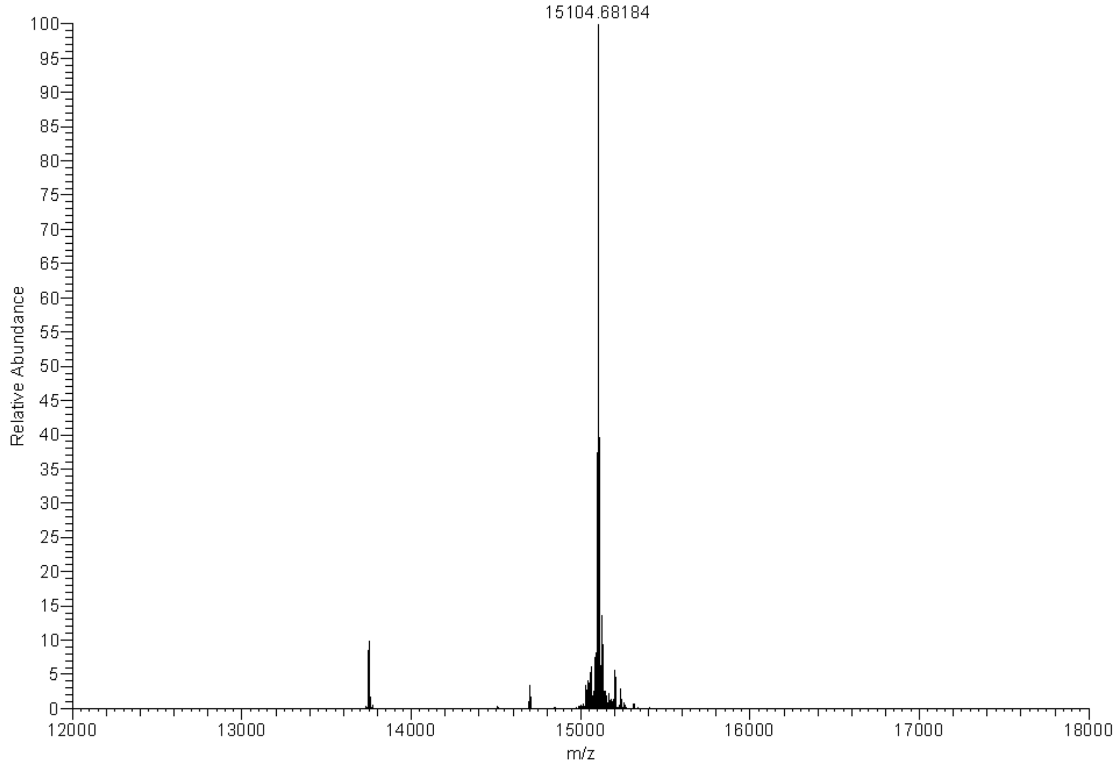

LmrR_LM_M89X_F93D calculated mass (-Met) 15033.61

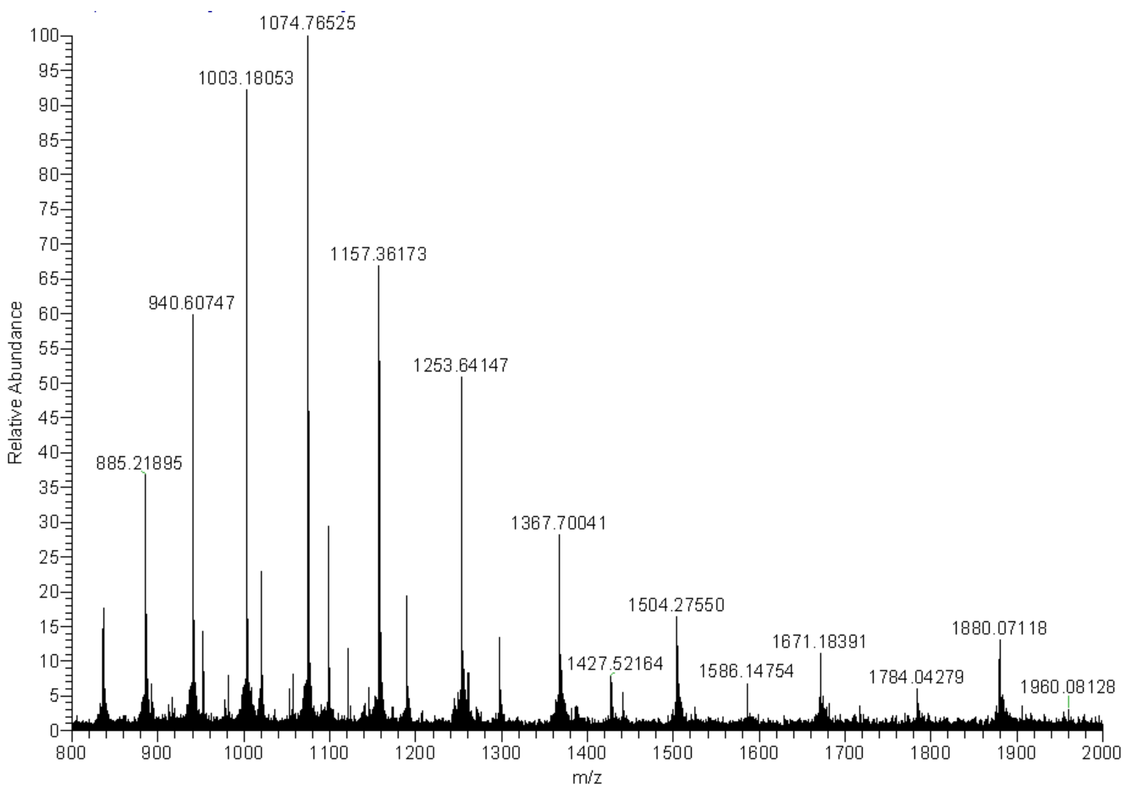

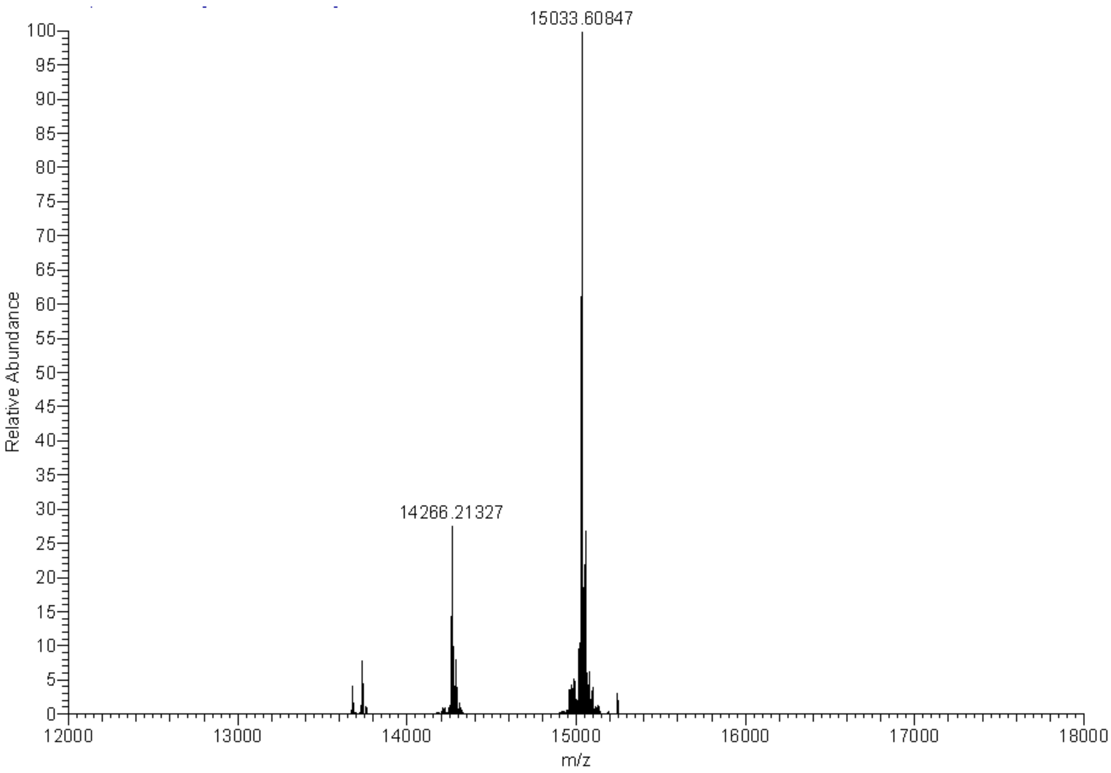

14266.21 – protein without terminal last 6 amino acids (6 out of 8 amino acids of Strep-tag)

LmrR_LM_M89X_N19A_E107A calculated mass (-Met) 14964.55

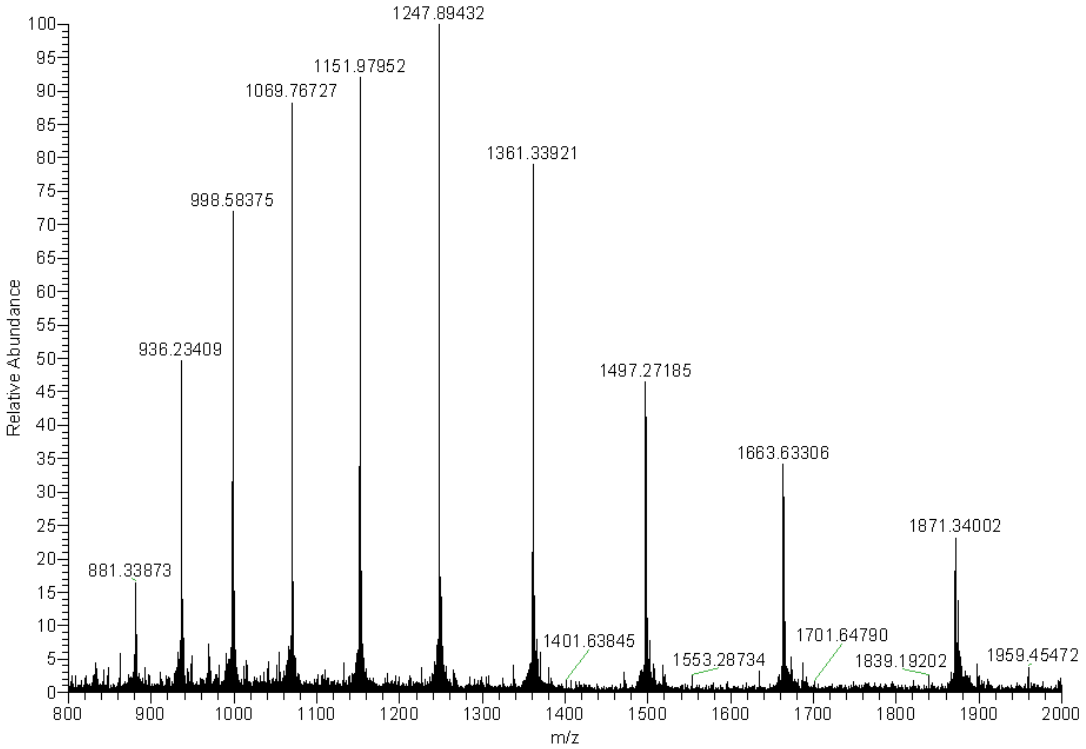

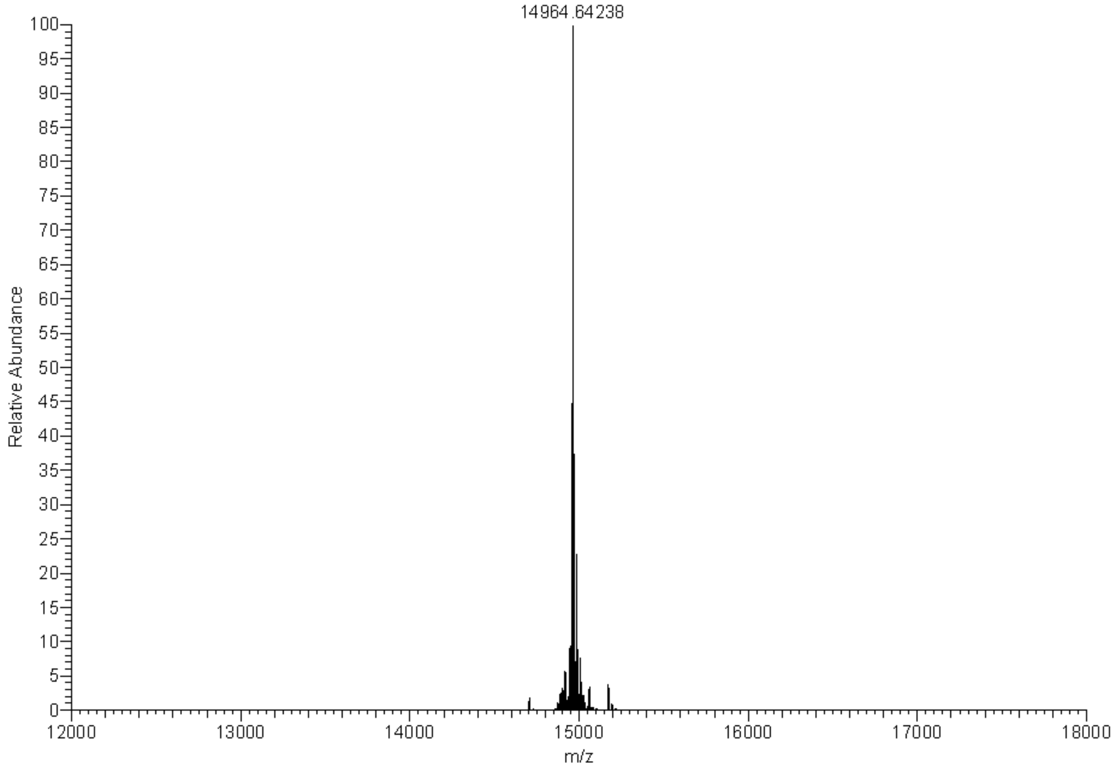

LmrR_LM_M89X_H86A_E107A calculated mass (-Met) 14941.60

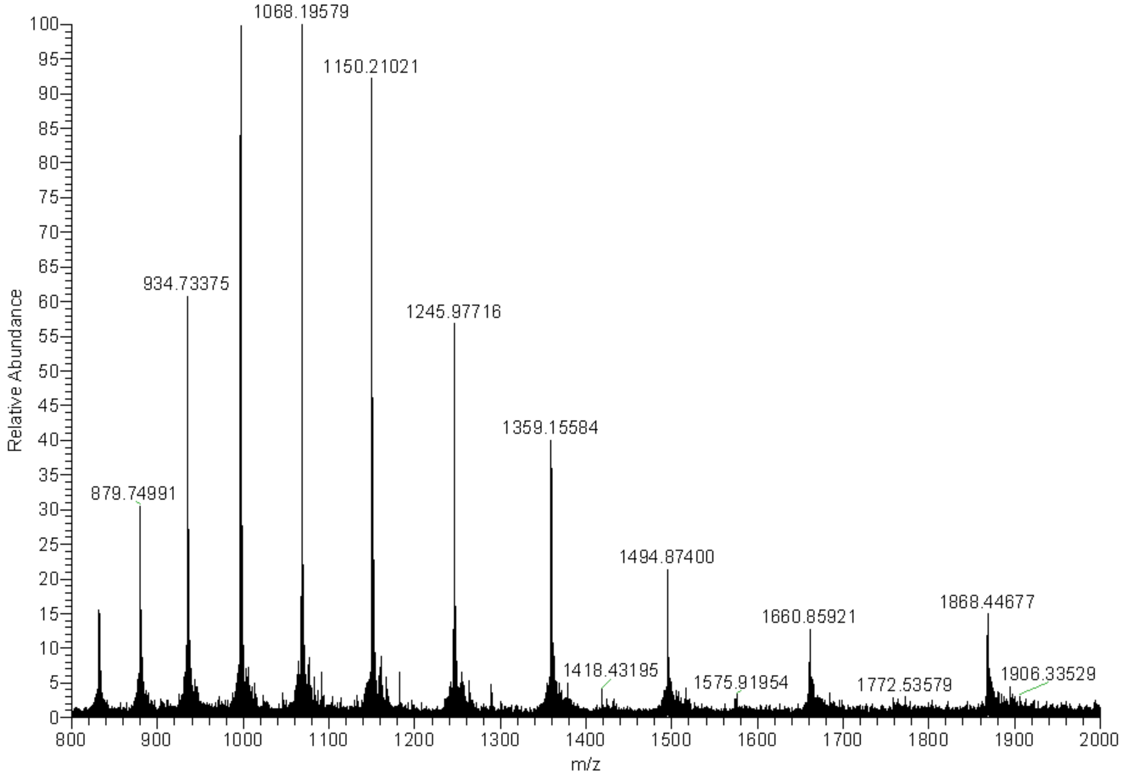

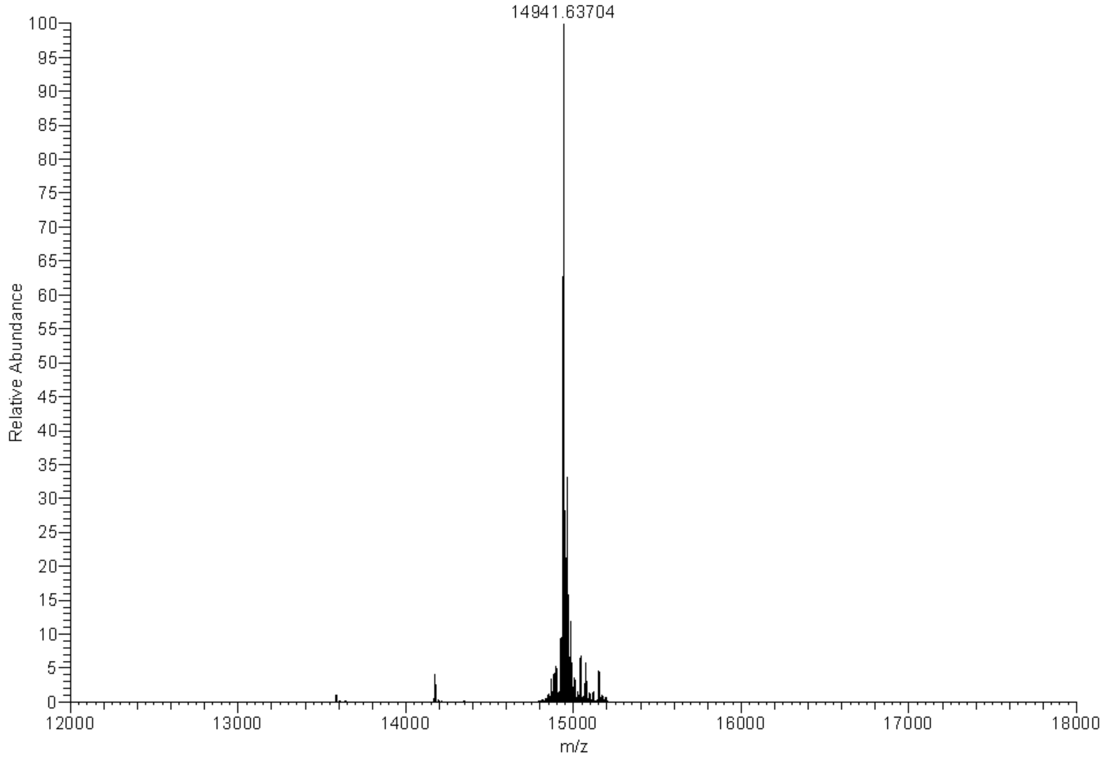

8. Characterization of the binding of Cu(II) to BpyAla

I. UV-VIS spectroscopy

Figure S4. a) Absorption Spectra of BpyAla after addition of different concentrations of Cu(NO$_{3)2}$ of Cu(NO$_{3)2.}$ **b)** Absorption spectra of LmrR (M89) after addition of different concentrations

a)

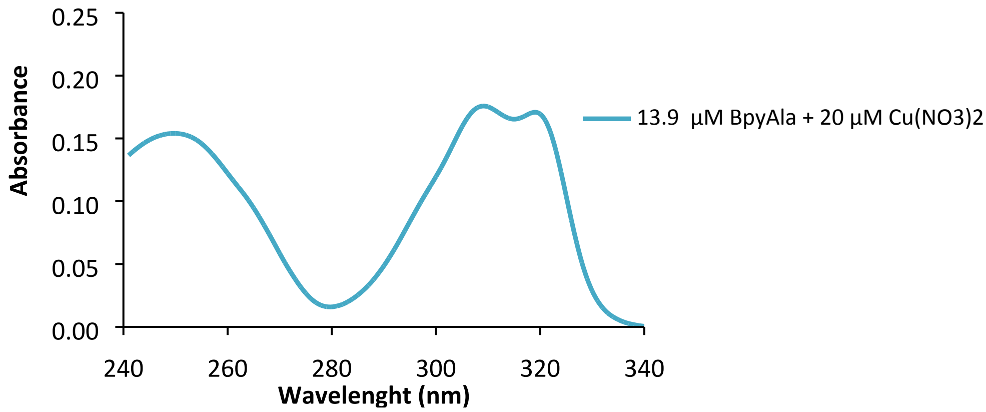

b)

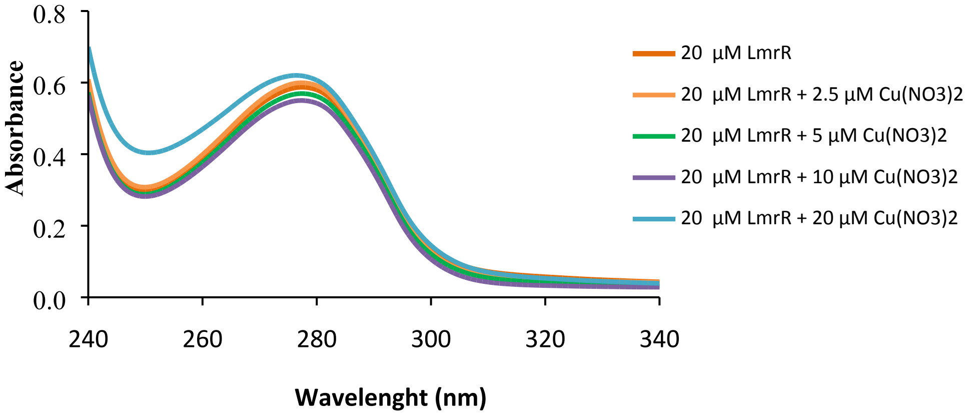

c)

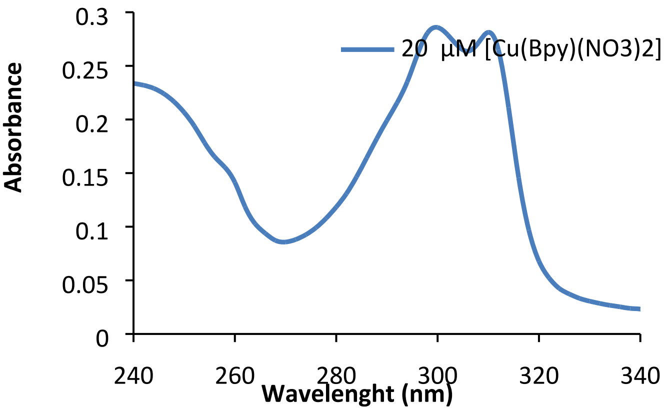

II. Raman spectroscopy

Experimental procedure of Raman spectroscopy measurement

Raman spectra were obtained in a ca. 155° backscattering arrangement with excitation at 355 nm (10 mW, Cobolt Zouk). Raman scattering was collected and collimated (plano-convex, dia. 25 mm, f = 7.5 cm), passed through a long pass filter to reject Rayleigh scattering (Semrock) and subsequently refocused (plano-convex, dia. 25 mm, f = 17.5 cm) into and dispersed by a Shamrock500i spectrograph (Andor Technology) with a 2400 l/mm blazed at 300 nm and acquired with a DV420A-BU2 CCD camera (Andor Technology). Data were recorded and processed using Solis (Andor Technology), Spekwin32[5] and Spectrum (Perkin Elmer) with spectral calibration performed using the Raman spectrum of acetonitrile/toluene 50:50 (v:v).[6] Samples were held in 10 mm path length quartz cuvettes. Solvent subtraction and a multipoint baseline correction were performed for all spectra.

III. EPR

Experimental procedure

EPR spectra (X-band, 9.46 GHz) were recorded on a Bruker ECS106 spectrometer in liquid nitrogen (77 K). Experimental conditions: Microwave frequency = 9.46 GHz; microwave power = 20 mW; 10 G field modulation amplitude; time constant 81.92 ms; scan time 83.89 s; 3 accumulations

Figure S5 EPR spectra of Cu(II)-BpyAla (240 μM) and LmrR-BpyAla-Cu(II) (240 μM of Cu(II)) in 20 mM MOPS buffer, 150 mM NaCl at pH 7 at 77 K.

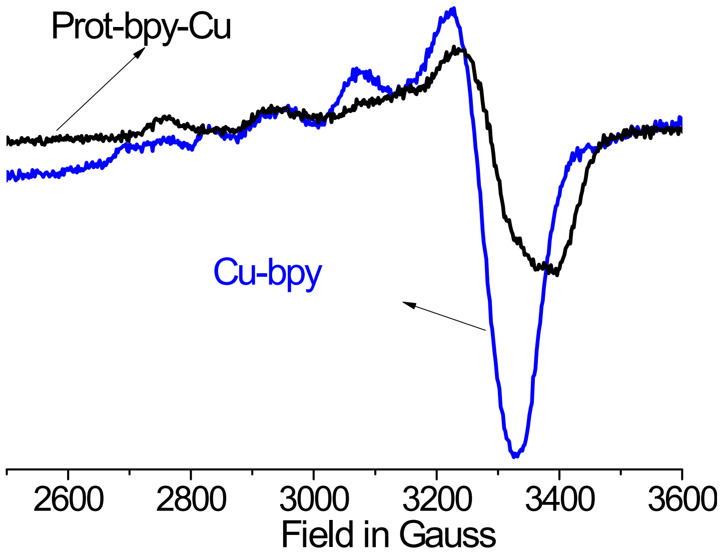

9. Catalysis – Friedel-Crafts reaction

Representative procedure for LmrR_LM_X_Cu(II) catalysed Friedel-Crafts reaction

The catalytic solution was prepared by combining $Cu(H_2O)_6(NO_3)_2$ (90 μM, 9 % catalyst loading) in MOPS buffer (20 mM MOPS, 150mM NaCl, pH 7.0) with 1.25 equivalents of LmrR_LM_X (112.5 μM) to a final volume of 280 μL. To this 10 μL of a fresh stock solution of substrate **1** in CH_3CN (final concentration 2.5 mM) and 10 μL of solution of substrate **2** in MOPS/CH_3CN was added (final concentration 1 mM). The reaction was mixed for 3 days by continuous inversion at 4 ^{0}C. The product was extracted with 3x 1 mL of diethyl ether, the organic layers were dried on Na_2SO_4 and evaporated under reduced pressure. The product was redissolved in 150 μl of a heptane:propan-2-ol mixture (10:1) and the conversion and enantiomeric excess were determined using HPLC (Chiralpak-AD n-heptane:iPrOH 90:10, 1ml/min).

Characterization data of Friedel-Crafts alkylation products[7]

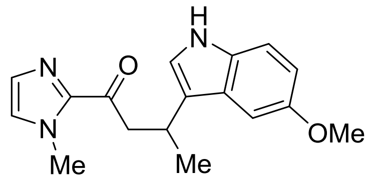

3-(5-methoxy-1H-indol-3-yl)-1-(1-methyl-1H-imidazol-2-yl)butan-1-one (3a). ^{1}H-NMR ($CDCl_3$, 400 MHz) δ 1.41 (d, 3H, J = 6.9 Hz), 3.39 (dd, 1H, J = 8.3 Hz, J = 15.6 Hz), 3.58 (dd, 1H, J = 6.2, J = 15.6 Hz), 3.77 – 3.84 (m, 1H), 3.86 (s, 3H), 3.94 (s, 3H), 6.82 (dd, 1H, J = 2.5 Hz, 8.8 Hz), 7.00 (s, 1H), 7.03 (d, 1H, J = 2.4 Hz), 7.15 (s, 1H), 7.15 – 7. 16 (m, 1H), 7.20 (s, 1H), 7.21 (d, 1H, J = 8.8 Hz), 8.05 (s, 1H). ^{13}C-NMR ($CDCl_3$, 400 MHz) δ 21.5 (q), 27.2 (q), 36.2 (d), 46.7 (q), 55.9 (t), 101.0 (d), 111.7 (d), 112.1 (d), 120.9 (d), 121.2 (s), 126.9 (d), 126.9 (s), 128.9 (d), 131.5 (s), 143.3 (s), 153.7 (s), 192.4 (s). HRMS (ESI) calcd for $C_{17}H_{20}N_3O_2$: 298.1550 ($[M+H]^+$), found 298.1550 ($[M+H]^+$). Ee's were determined by HPLC analysis (Chiralcel-AD, heptane/iPrOH 90:10, 1 mL/min). Retention times: 33.0 (+) and 39.3 (-) mins.

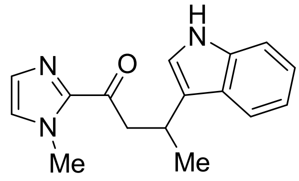

3-(1H-indol-3-yl)-1-(1-methyl-1H-imidazol-2-yl)butan-1-one (3b). ^{1}H-NMR (CD_3OD, 400 MHz) δ 1.40 (d, 3H, J = 6.9 Hz), 3.39 (m, 2H), 3.76 (m, 1H), 3.83 (s, 3H), 6.95 (t, 1H, J = 7.1 Hz), 7.05 (t, 1H, J = 8.2 Hz), 7.05 (s, 1H), 7.10 (s, 1H), 7.24 (s, 1H), 7.29 (d, 1H, J = 8.1 Hz), 7.47 (d, 1H, J = 7.9 Hz). ^{13}C-NMR ($CDCl_3$, 400 MHz) δ 21.7 (q), 27.2 (t), 36.2 (d), 46.7 (q), 111.0 (d), 119.1 (d), 119.3 (d), 120.2 (d), 121.5 (s), 121.8 (d), 126.6 (s), 126.8 (d), 128.9 (d), 136.4 (s), 143.4 (s), 192.3 (s). Ee's were determined by HPLC analysis (Chiralcel-AD, heptane/iPrOH 90:10, 1 mL/min). Retention times: 25.4 (+) and 33.2 (-) mins.

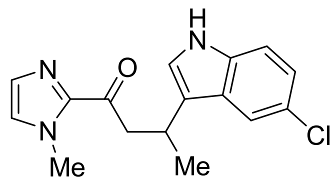

3-(5-chloro-1H-indol-3-yl)-1-(1-methyl-1H-imidazol-2-yl)butan-1-one (3c). ^{1}H-NMR (CD_3OD, 400 MHz) δ 1.40 (d, 3H, J = 6.9), 3.35 (d, 2H, J = 7.2 Hz), 3.71 (m, 1H), 3.38 (s, 3H), 7.01 (dd, 1H, J = 8.6 Hz, J = 2.0 Hz), 7.11 (d, 1H, J = 0.9 Hz), 7.12 (s, 1H), 6.25 (d, 1H, J = 8.6 Hz), 7.27 (s, 1H), 7.28 (d, 1H, J = 2.0 Hz). ^{13}C-NMR ($CDCl_3$, 400 MHz) δ 21.6 (q), 27.1 (d), 36.1 (q), 46.9 (t), 112.0 (d), 118.7 (d), 121.3 (s), 121.6 (d), 122.1 (d), 124.8 (s), 127.0 (d), 127.7 (s), 128.9 (d), 134.7 (s), 143.2 (s), 192.0 (s). HRMS (ESI) calcd for $C_{16}H_{17}N_3O$: 302.1055 ($[M+H]^+$), found 302.1054 ($[M+H]^+$). Ee's were determined by HPLC analysis (Chiralcel-AD, heptane/iPrOH 90:10, 1 mL/min). Retention times: 18.4 (+) and 22.1 (-) mins.

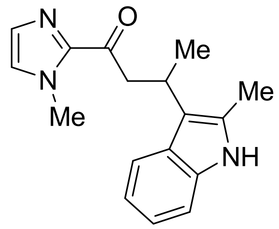

1-(1-methyl-1H-imidazol-2-yl)-3-(2-methyl-1H-indol-3-yl)butan-1-one (3d). ^{1}H-NMR (CD_3OD, 400 MHz) δ 1.45 (d, 3H, J = 7.1 Hz), 2.31 (s, 3H), 3.41 – 3.53 (m, 2H), 3.73 (s, 3H), 3.70 – 3.77 (m, 1H), 6.88 (t, 1H, J = 7.0 Hz), 6.94 (t, 1H, J = 7.8 Hz), 7.05 (s, 1H), 7.14 (d, 1H, J = 9.2 Hz), 7.19 (s, 1H), 7.49 (d, 1H, J = 7.6 Hz). ^{13}C-NMR ($CDCl_3$, 400 MHz) δ 12.3 (q), 21.1 (d), 27.0 (q), 36.0 (q), 46.2 (t), 110.1 (d), 115.4 (s), 118.9 (d), 119.2 (d), 120.5 (d), 126.5 (d), 127.4 (s), 128.8 (d), 130.3 (s), 135.3 (s), 143.3 (s), 192.3 (s). HRMS (ESI) calcd for $C_{17}H_{20}N_3O$: 282.1601 ($[M+H]^+$), found 282.1600 ($[M+H]^+$). Ee's were determined by HPLC analysis (Chiralcel-AD, heptane/iPrOH 90:10, 1 mL/min). Retention times: 16.0 (-) and 21.9 (+) mins.

Table S2 Results of the control experiments of vinylogous Friedel-Crafts reaction of **1a/b/c/d** and **2** resulting in **3a/b/c/d**.[a]

Entry	Catalyst	Substrate	Product	Conversion (%)	*ee* (%)
1	LmrR_LM_**N19X**	**1a**	**3a**	<1	-
2	LmrR_LM_**M89X**	**1a**	**3a**	<1	-
3	LmrR_LM_**F93X**	**1a**	**3a**	<1	-
4	LmrR_LM_**M89X_K22A**	**1a**	**3a**	<1	-
5	LmrR_LM_M89X_**H86A**	**1a**	**3a**	<1	-
6	*L*-BpyAla_Cu^{II}	**1a**	**3a**	74 ± 6	<5
7	*L*-BpyAla_Cu^{II}	**1b**	**3b**	84 ± 10	<5
8	*L*-BpyAla_Cu^{II}	**1c**	**3c**	72 ± 9	<5
9	*L*-BpyAla_Cu^{II}	**1d**	**3d**	89 ± 5	<5

[a] Typical conditions: Entry 1-5 112.5 µM LmrR_LM_X in 20 mM MOPS buffer (pH 7.0), 150 mM NaCl, for 3 days at 4 °C. Entry 6-9 9 mol% $Cu(H_2O)_6(NO_3)_2$ (90 µM) loading with 1.25 eq of ligand *L*-BpyAla (92% *ee*, see above, chapter 2) in 20 mM MOPS buffer (pH 7.0), 150 mM NaCl, for 3 days at 4 °C.

Table S3. Effect of catalyst loading on the results of the vinylogous Friedel-Crafts alkylation reaction of **1a** and **2** resulting in **3a**.[a]

$Cu(H_2O)_6(NO_3)_2$	Catalyst	conversion (%)	ee (%)
30 µM	LmrR_LM_**N19X**_Cu^{II}	15 ± 4	19 ± 3 (+)
90 µM	LmrR_LM_**N19X**_Cu^{II}	18 ± 2	29 ± 2 (+)
30 µM	LmrR_LM_**M89X**_Cu^{II}	29 ± 3	36 ± 4 (+)
90 µM	LmrR_LM_**M89X**_Cu^{II}	27 ± 6	49 ± 4 (+)
30 µM	LmrR_LM_**F93X**_Cu^{II}	37 ± 4	8 ± 2 (-)
90 µM	LmrR_LM_**F93X**_Cu^{II}	36 ± 3	22 ± 1 (-)

[a] Typical conditions: 1.25 eq LmrR_LM_X compared to $Cu(H_2O)_6(NO_3)_2$, 20 mM MOPS buffer (pH 7.0), 150 mM NaCl, for 3 days at 4 °C

Figure S6 Chiral HPLC traces of products of the Friedel-Crafts reaction catalysed by **LmrR_LM_X_Cu^{II}.**

3a Chiralpak-AD n-heptane:iPrOH 90:10, 1ml/min

SHIMADZU LabSolutions

Analysis Report 3A

<Chromatogram>

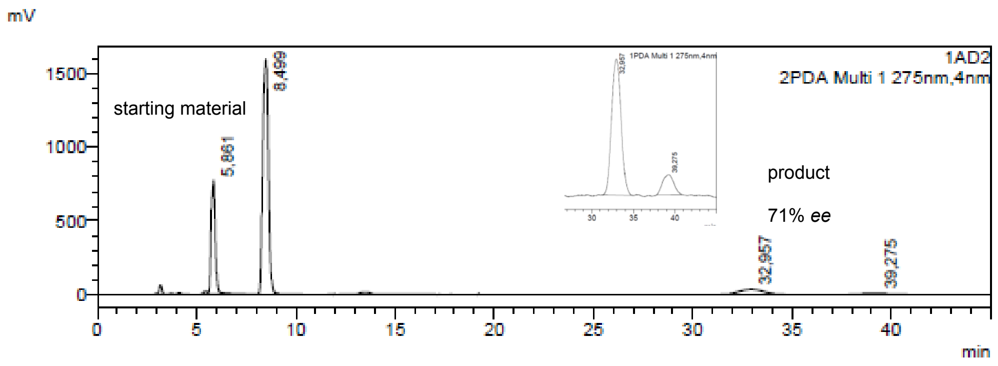

<Peak Table>

PDA Ch1 275nm

Peak#	Ret. Time	Height	Area	Area%
1	5,861	773753	11470081	24,553
2	8,499	1591244	32021235	68,544
3	32,957	34634	2763510	5,916
4	39,275	5073	461434	0,988
Total		2404705	46716261	100,000

SHIMADZU LabSolutions

Analysis report 3A-Racemate

<Chromatogram>

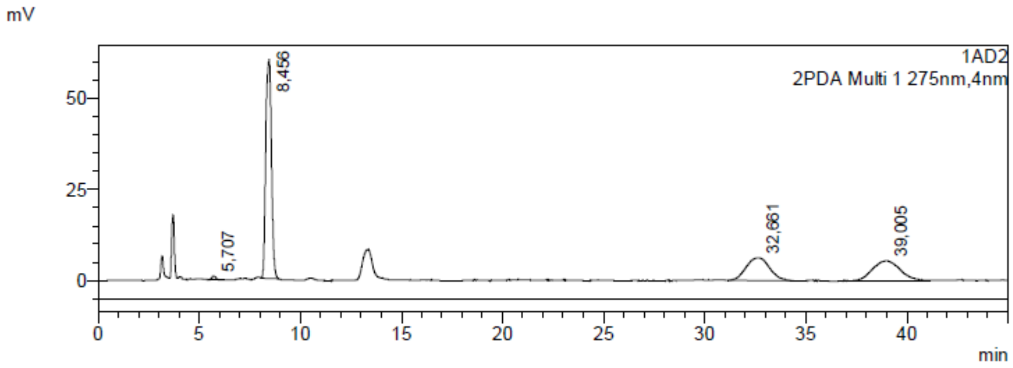

<Peak Table>

PDA Ch1 275nm

Peak#	Ret. Time	Height	Area	Area%
1	5,707	928	12529	0,576
2	8,456	60016	1173161	53,966
3	32,661	6209	496136	22,823
4	39,005	5335	492062	22,635
Total		72489	2173889	100,000

3b Chiralpak-AD n-heptane:iPrOH 90:10, 1ml/min

SHIMADZU LabSolutions

Analysis report 3B

<Chromatogram>

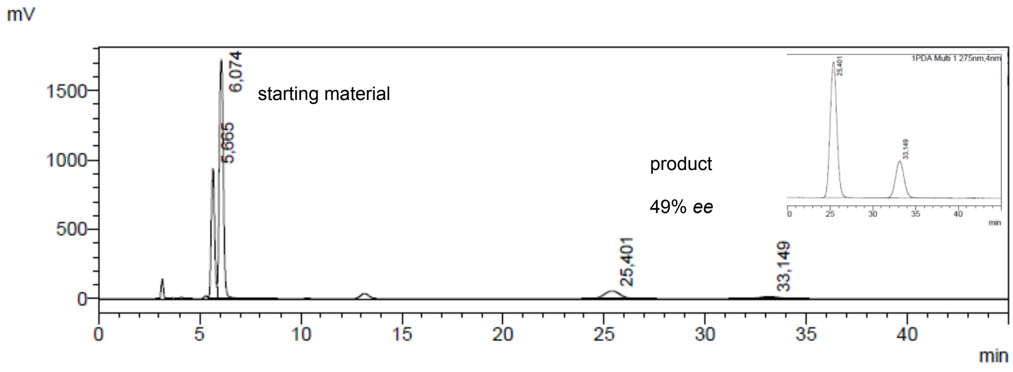

<Peak Table>

PDA Ch1 275nm

Peak#	Ret. Time	Height	Area	Area%
1	5,665	935025	10822279	27,385
2	6,074	1717401	24524137	62,057
3	25,401	56967	3103921	7,854
4	33,149	15453	1068089	2,703
Total		2724846	39518426	100,000

SHIMADZU LabSolutions

Analysis report 3B-Racemate

<Chromatogram>

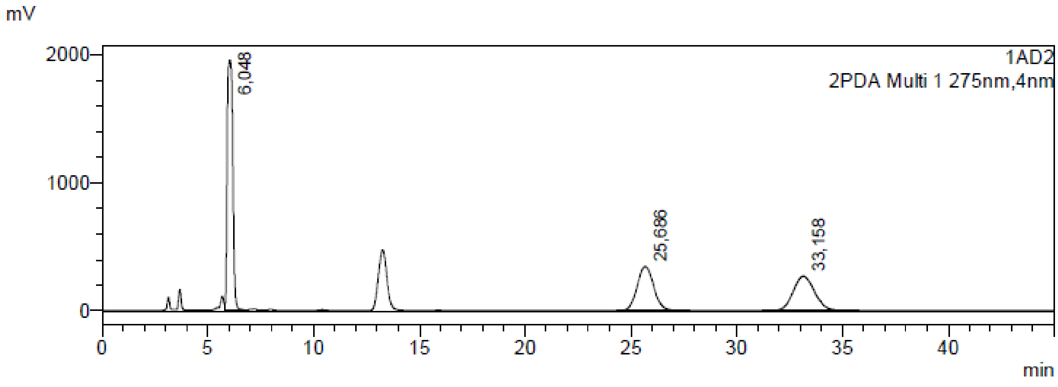

<Peak Table>

PDA Ch1 275nm

Peak#	Ret. Time	Height	Area	Area%
1	6,048	1953598	35192368	48,430
2	25,686	342539	18630056	25,638
3	33,158	270802	18843622	25,932
Total		2566939	72666046	100,000

3c Chiralpak-AD n-heptane:iPrOH 90:10, 1ml/min

SHIMADZU LabSolutions

Analysis report 3C

<Chromatogram>

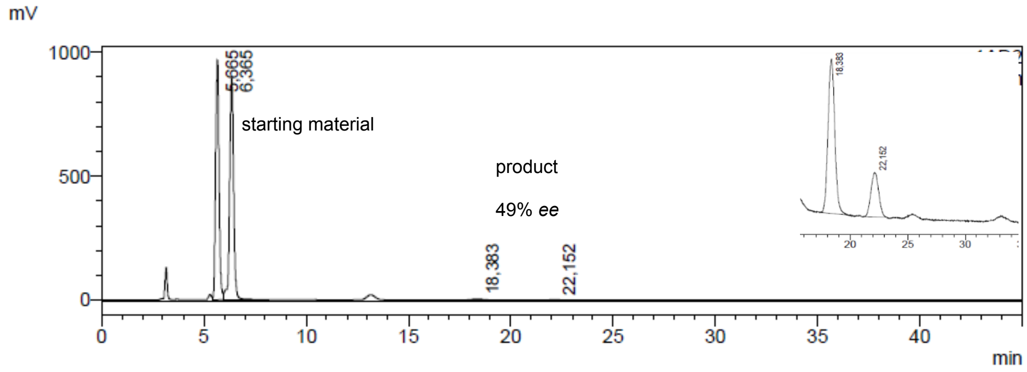

<Peak Table>

PDA Ch1 275nm

Peak#	Ret. Time	Height	Area	Area%
1	5,665	968032	11386171	47,487
2	6,365	892397	12370318	51,592
3	18,383	4157	164200	0,685
4	22,152	1216	56745	0,237
Total		1865802	23977434	100,000

SHIMADZU LabSolutions

Analysis report 3C-Racemate

<Chromatogram>

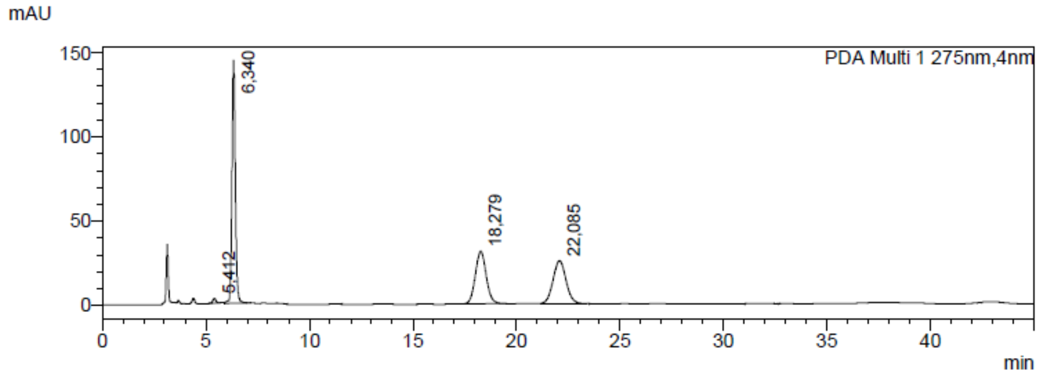

<Peak Table>

PDA Ch1 275nm

Peak#	Ret. Time	Height	Area	Area%
1	5,412	2883	31925	0,810
2	6,340	144297	1659875	42,138
3	18,279	31257	1144238	29,048
4	22,085	25450	1103093	28,003
Total		203887	3939131	100,000

3d Chiralpak-AD n-heptane:iPrOH 90:10, 1ml/min

SHIMADZU LabSolutions

Analysis report 3D

<Chromatogram>

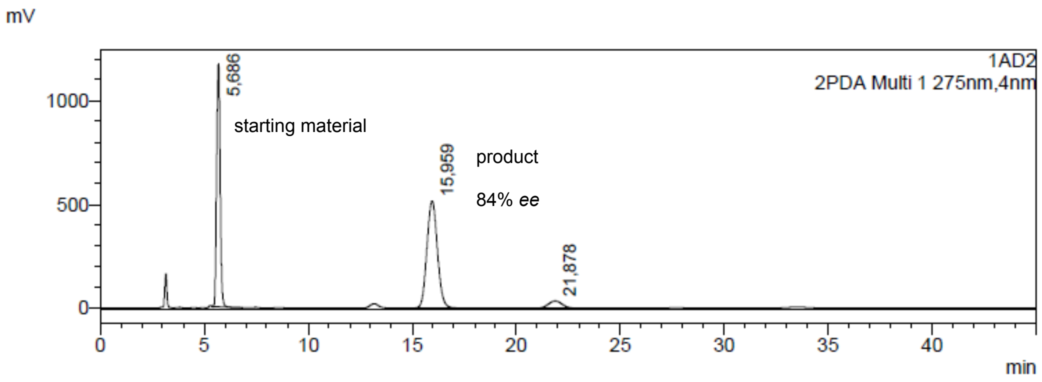

<Peak Table>

PDA Ch1 275nm

Peak#	Ret. Time	Height	Area	Area%
1	5,686	1171769	13819003	41,060
2	15,959	516652	18204096	54,090
3	21,878	35177	1632369	4,850
Total		1723598	33655468	100,000

SHIMADZU LabSolutions

Analysis report 3D-Racemate

<Chromatogram>

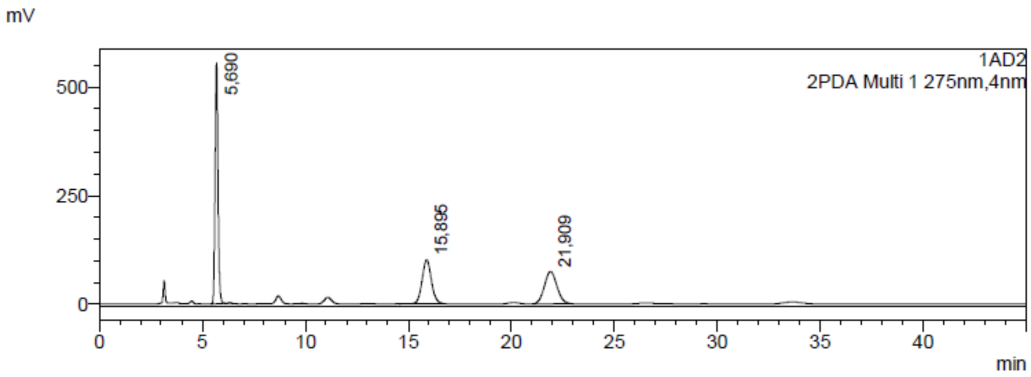

<Peak Table>

PDA Ch1 275nm

Peak#	Ret. Time	Height	Area	Area%
1	5,690	554288	5454685	46,404
2	15,895	101102	3165296	26,928
3	21,909	74091	3134885	26,669
Total		729481	11754866	100,000

9. References

1. N. C. Fletcher, M. Nieuwenhuyzen, and S. Rainey, *J. Chem. Soc. Dalt. Trans.*, 2001, 2641.
2. B. Imperiali, T. J. Prins, and S. L. Fisher, *J. Org. Chem.*, 1993, **58**, 1613.
3. J. Xie, W. Liu, and P. G. Schultz, *Angew. Chem. Int. Ed. Engl.*, 2007, **46**, 9239.
4. J. Bos, A. García-Herraiz, and G. Roelfes, *Chem. Sci.*, 2013, **4**, 3578.
5. F. Menges, "Spekwin32 - optical spectroscopy software", Version 1.71.6.1, 2012, http://www.effemm2.de/spekwin/
6. ASTM E1840-96(2007) Standard Guide for Raman Shift Standards for Spectrometer Calibration, ASTM International, **DOI:** 10.1520/E1840-96R07
7. A.J. Boersma, B.L. Feringa and G. Roelfes *Angew. Chem. Int. Ed. Engl.*2009, 48, 3346
